# Supplementary material for: Multiyne chains chelating osmium via three metal-carbon σ bonds
Source: Nat Commun. 2017 Dec 4;8:1912. doi: 10.1038/s41467-017-02120-z (PMC5714968; doi:10.1038/s41467-017-02120-z)
Supplement: Supplementary file 1 — Supplementary Information [file 41467_2017_2120_MOESM1_ESM.pdf]

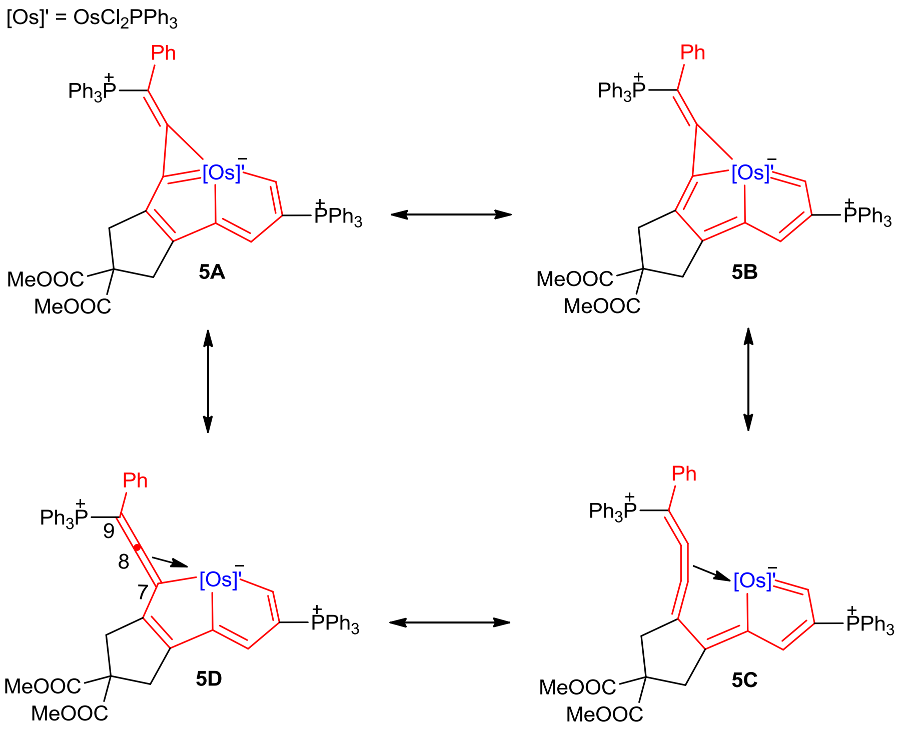

Supplementary Figure 1. Major resonance structures of the cation moiety of complex **5**. Both the single-crystal (Fig. 3b) and NMR data (see Supplementary Fig. 80 to 82) indicate that **5** possesses fused metallapentalene structures with a metallacyclopropenylidene moiety, as shown in resonance structures **5A** and **5B**. Alternatively, one might consider species **5C** and **5D** containing a carbon–carbon double bond coordinated to the metal centers as two other possible resonance forms. However, as shown in Supplementary Fig. 2, substantial bonding between the metal center and the C8 atom was revealed by the natural bond orbital (NBO) analysis. The Wiberg bond indices of Os–C1, Os–C4, Os–C7, and Os–C8 quantify the distinct interaction between the metal centers and the C8 atoms. In addition, the component of the p-orbital on the C8 atom in the Os–C8 bond is 74.08%, suggesting it is closer to that (66.67%) of an sp^2 center rather than to that (50.00%) of an *sp* center. Therefore, we consider that the molecular structure of **5** is better described by a resonance hybrid of the four resonance forms and resonance structures **5A** and **5B** should be dominant.

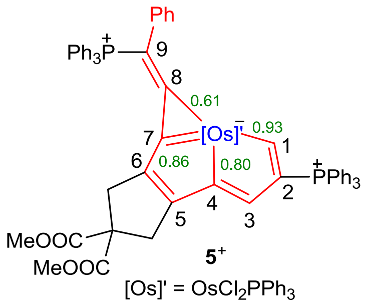

5^{+}

[Os]' = $OsCl_2PPh_3$

Supplementary Figure 2. Calculated Wiberg bond indices for the cation moiety of complex **5**.

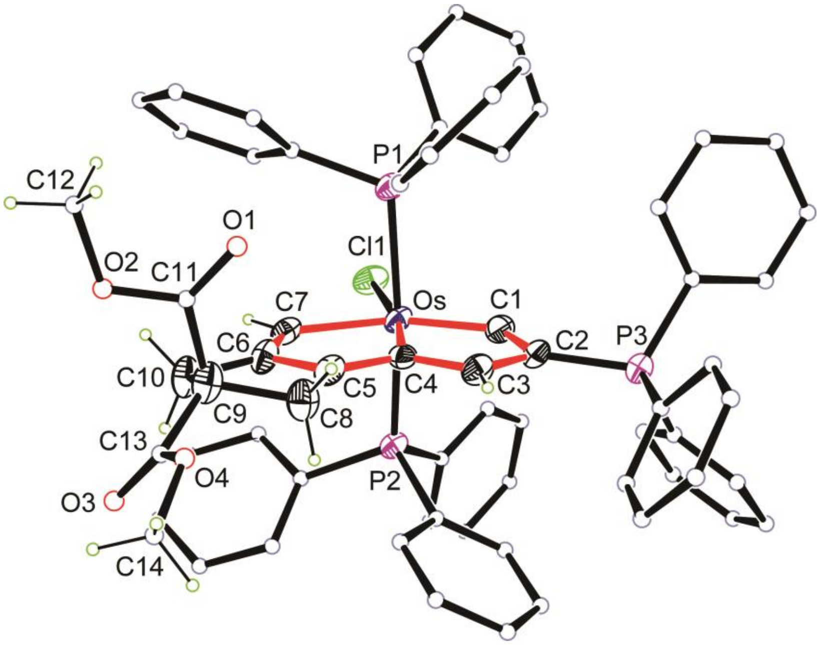

Supplementary Figure 3. X-ray molecular structure for the cation of complex **1** (ellipsoids are at the 50% probability level). Hydrogen atoms of PPh_3 are omitted for clarity.

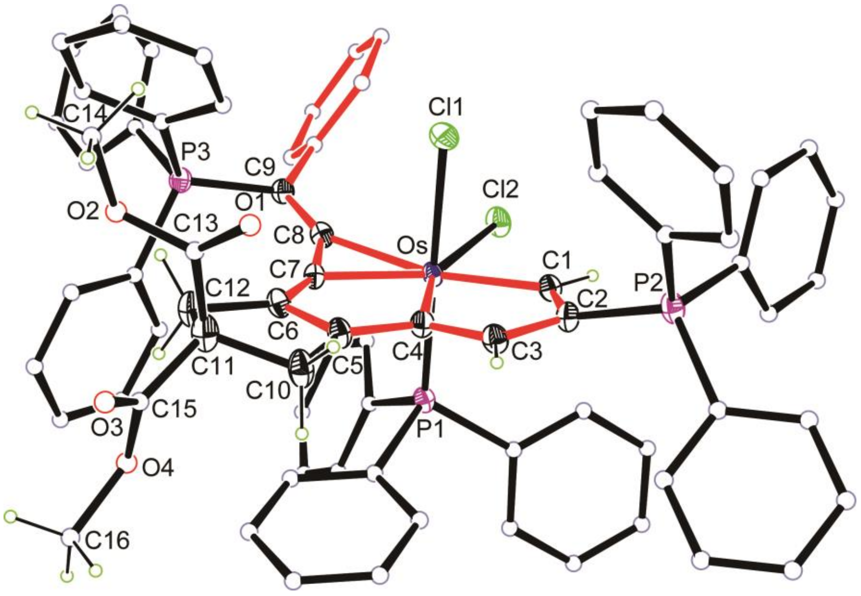

Supplementary Figure 4. X-ray molecular structure for the cation of complex **5-PF$_6$** (ellipsoids are at the 50% probability level). Hydrogen atoms of phenyl rings are omitted for clarity.

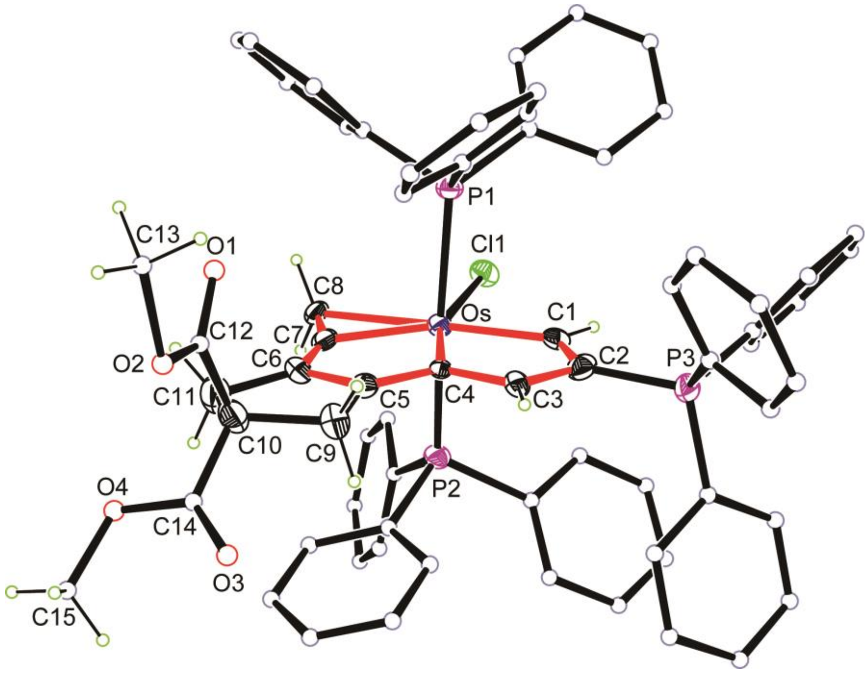

Supplementary Figure 5. X-ray molecular structure for the cation of complex **6** (ellipsoids are at the 50% probability level). Hydrogen atoms of phenyl rings are omitted for clarity.

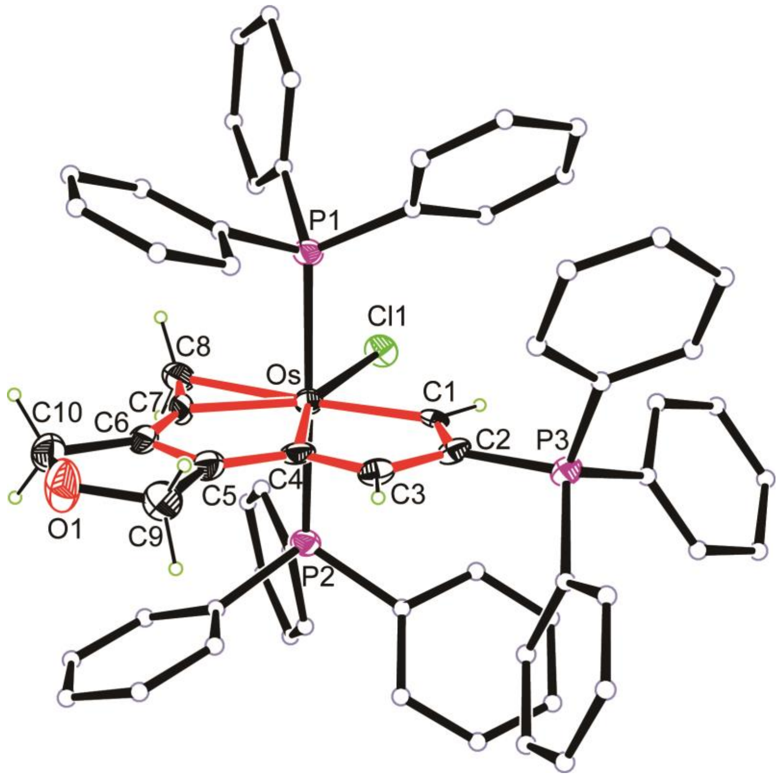

Supplementary Figure 6. X-ray molecular structure for the cation of complex **7** (ellipsoids are at the 50% probability level). Hydrogen atoms of phenyl rings are omitted for clarity. Selected bond distances (Å) and angles (deg): Os–C1 2.027(5), Os–C4 2.094(5), Os–C7 1.994(5), Os–C8 2.280(6), C1–C2 1.389(7), C2–C3 1.410(8), C3–C4 1.360(7), C4–C5 1.413(8), C5–C6 1.373(8), C6–C7 1.368(8), C7–C8 1.391(8), Os–C1–C2 120.5(4), C1–C2–C3 112.5(5), C2–C3–C4 114.0(5), C3–C4–Os 118.4(4), C1–Os–C4 74.5(2), Os–C4–C5 116.4(4), C4–C5–C6 114.9(5), C5–C6–C7 110.8(5), C6–C7–Os 124.4(4), C7–Os–C4 73.6(2), Os–C7–C8 82.6(4), C7–C8–Os 60.1(3), C8–Os–C7 37.2(2).

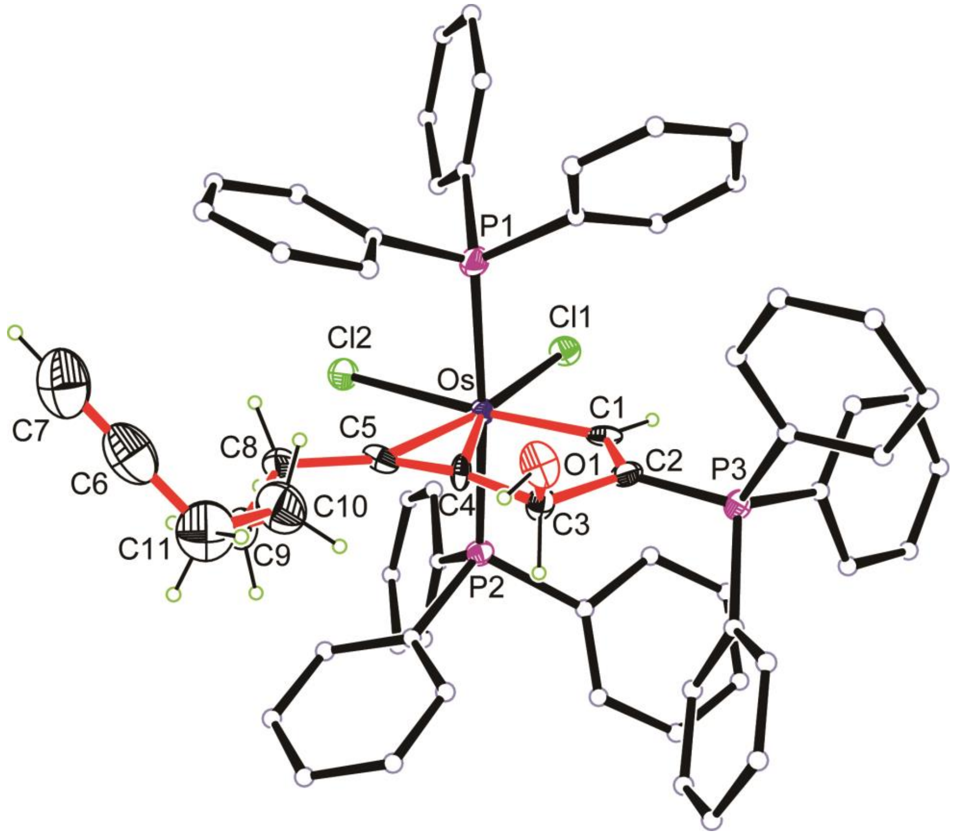

Supplementary Figure 7. X-ray molecular structure for complex **9** (ellipsoids are at the 50% probability level). Hydrogen atoms of phenyl rings are omitted for clarity.

[Os] = OsCl(PPh$_3$)$_2$, [Os]' = OsCl$_2$PPh$_3$

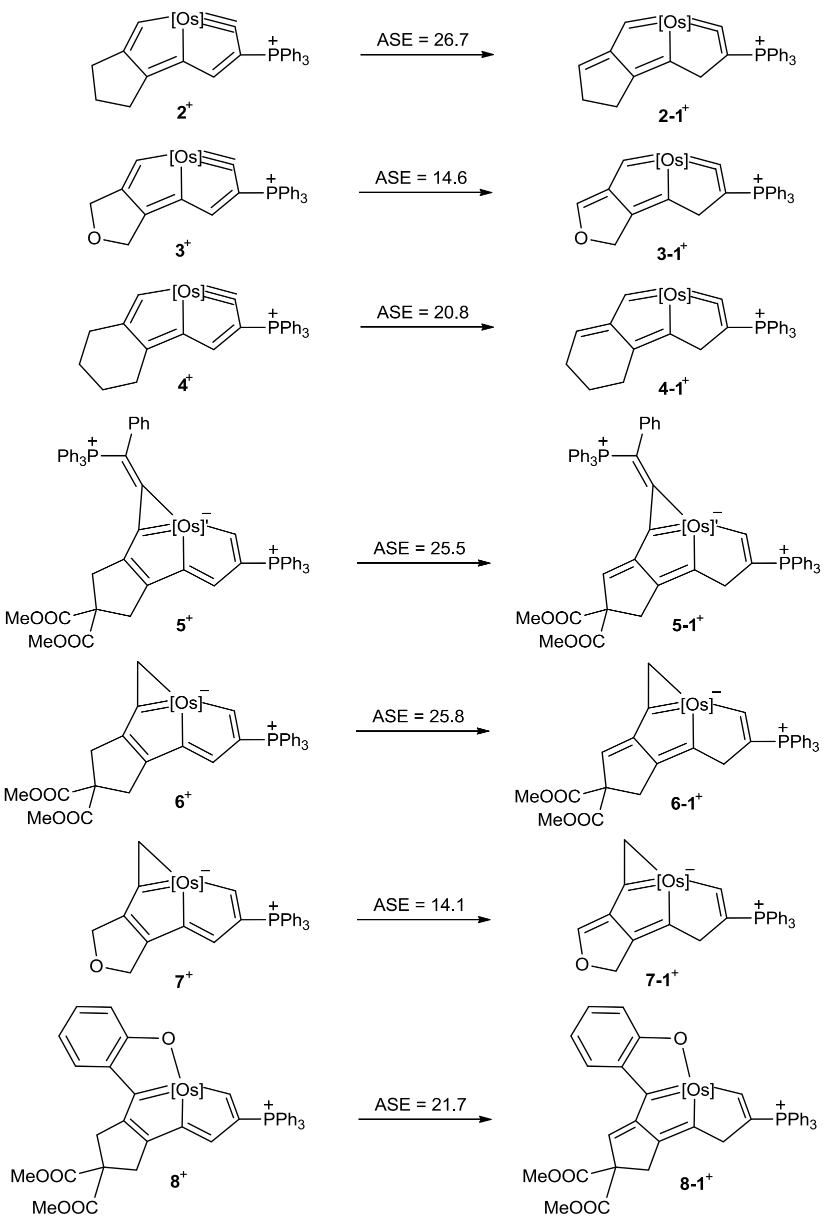

Supplementary Figure 8. ASE evaluations of the cation moieties of complexes **2**–**8**. The energies include the zero-point energy corrections are given in kcal/mol.

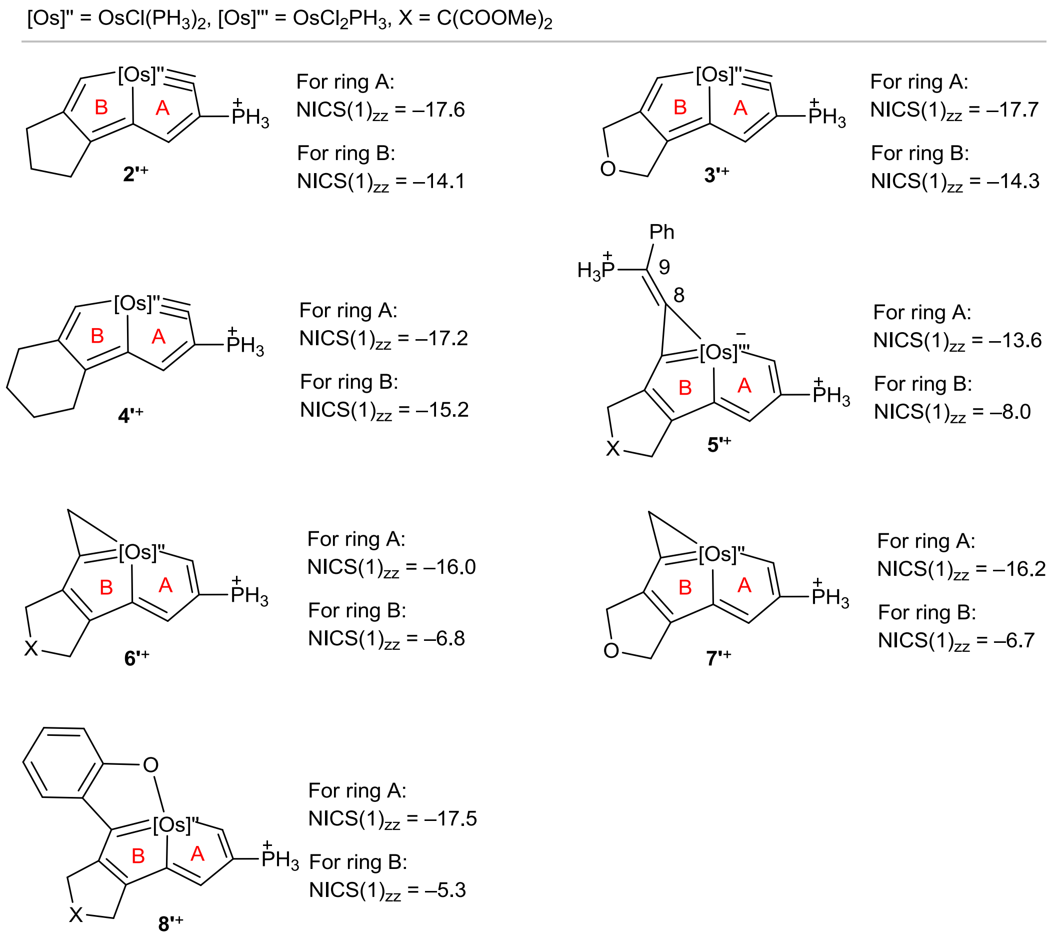

Supplementary Figure 9. NICS (in ppm) evaluations of the cation moieties of model complexes **2′**–**8′**.

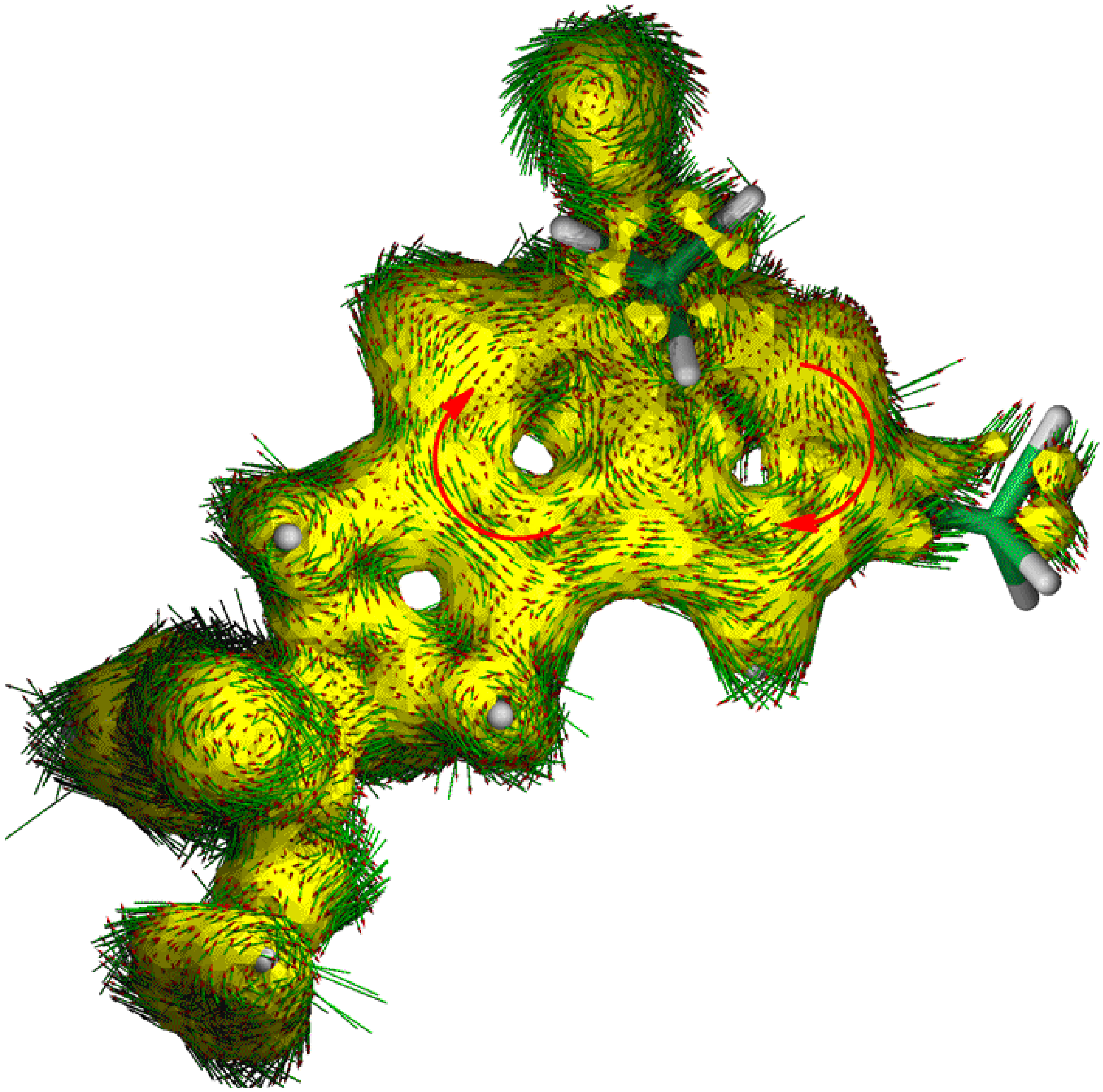

Supplementary Figure 10. AICD plot of the cation moiety of model complex **1′** with an isosurface value of 0.025. The magnetic field vector is orthogonal to the ring plane and points upward (clockwise currents are diatropic).

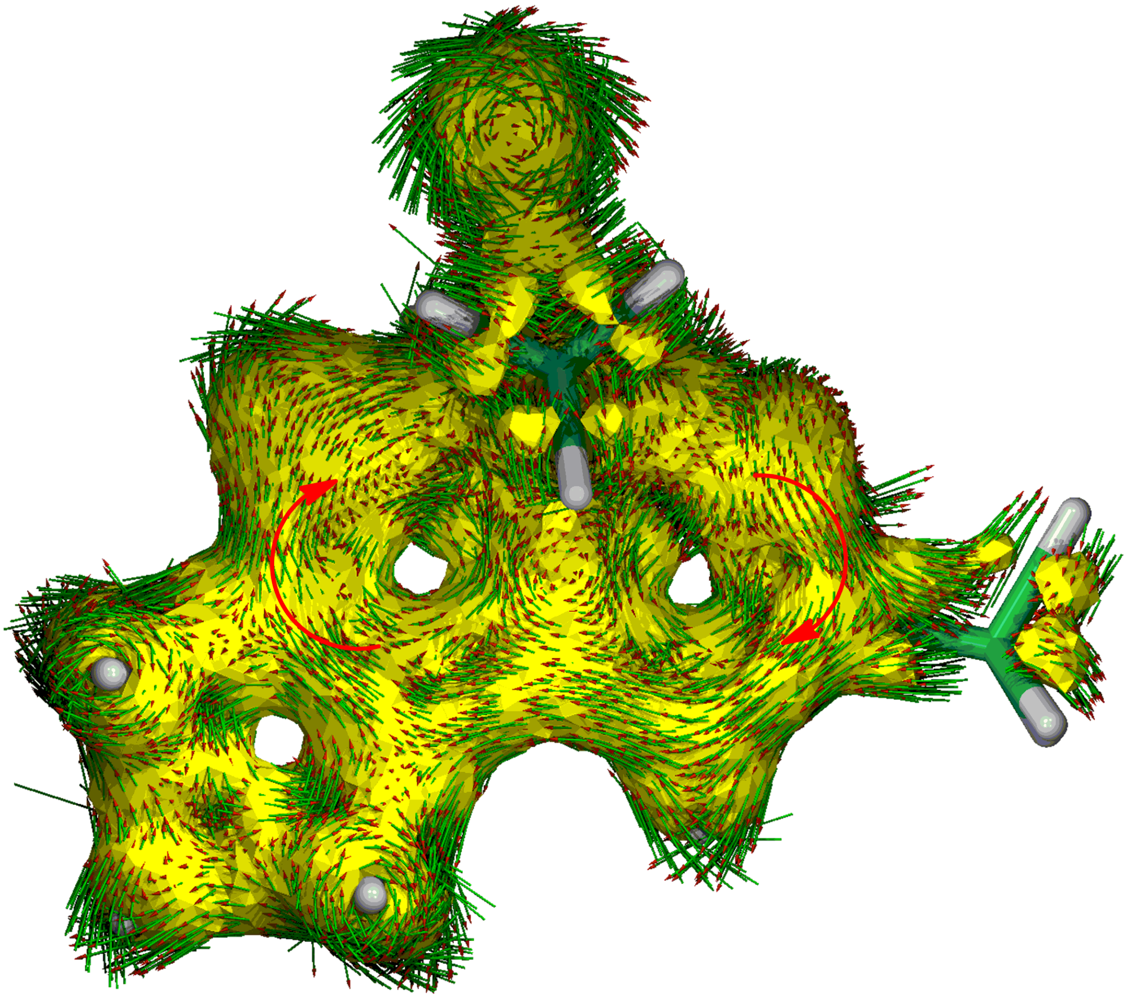

Supplementary Figure 11. AICD plot of the cation moiety of model complex **2′** with an isosurface value of 0.025. The magnetic field vector is orthogonal to the ring plane and points upward (clockwise currents are diatropic).

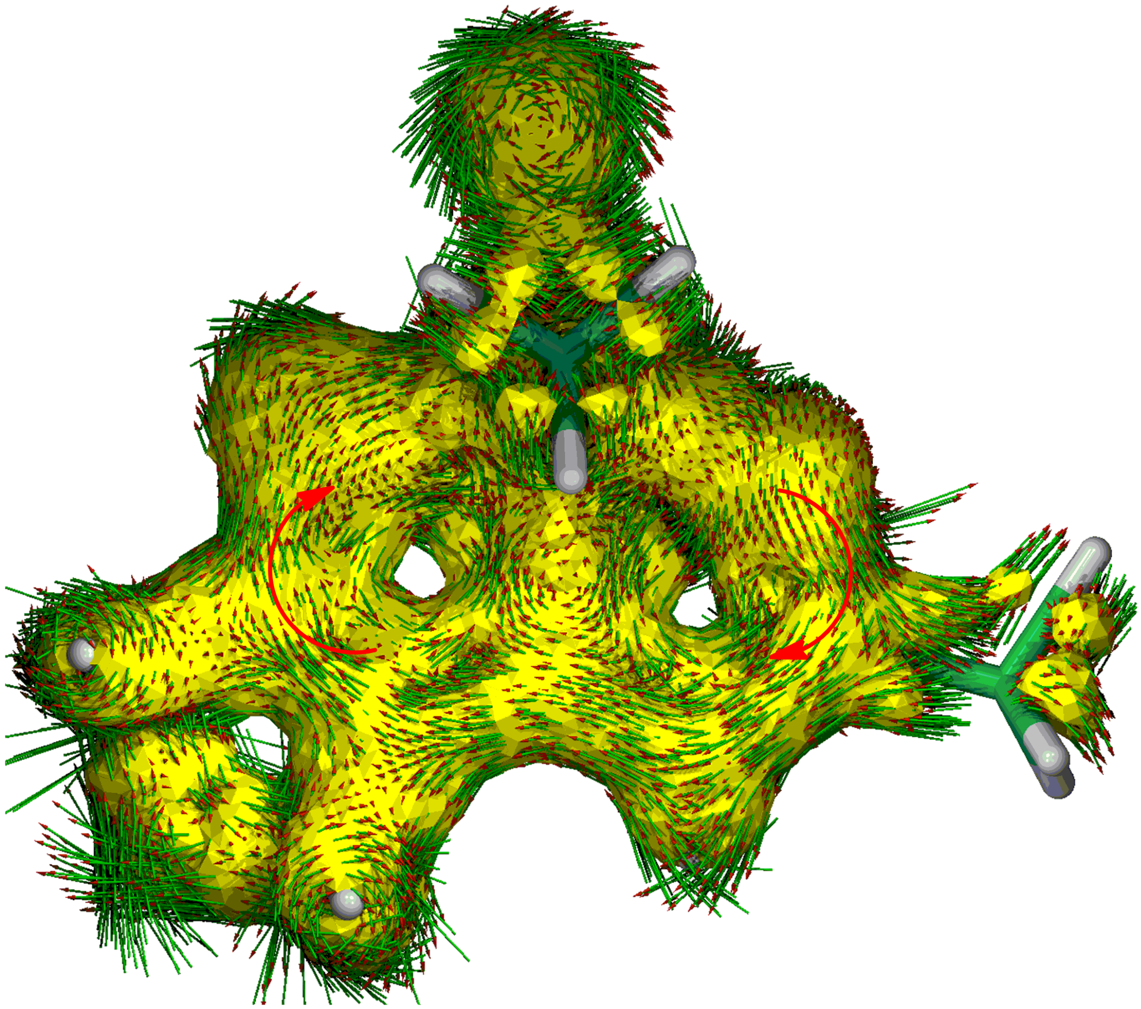

Supplementary Figure 12. AICD plot of the cation moiety of model complex **3′** with an isosurface value of 0.025. The magnetic field vector is orthogonal to the ring plane and points upward (clockwise currents are diatropic).

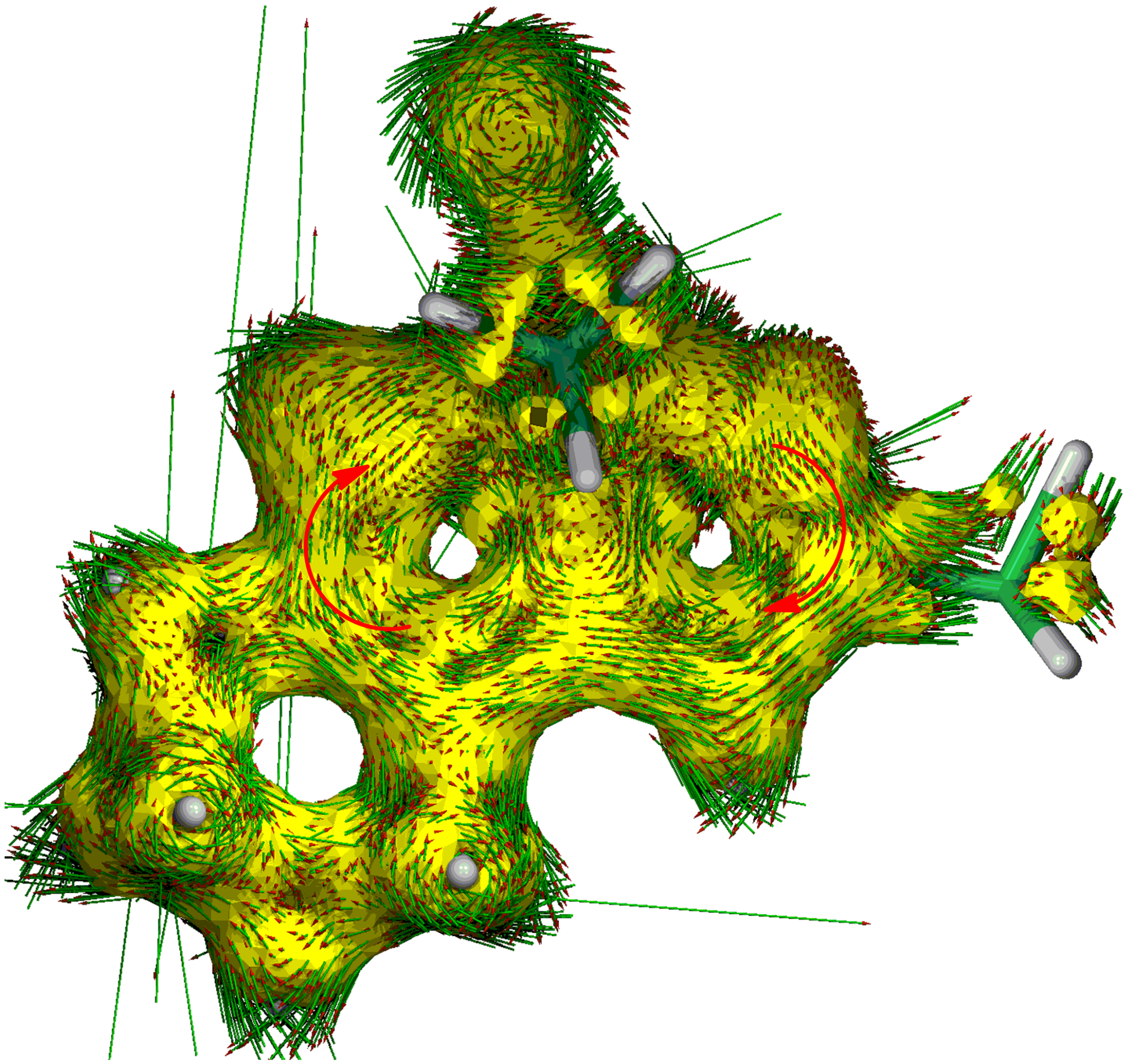

Supplementary Figure 13. AICD plot of the cation moiety of model complex **4′** with an isosurface value of 0.025. The magnetic field vector is orthogonal to the ring plane and points upward (clockwise currents are diatropic).

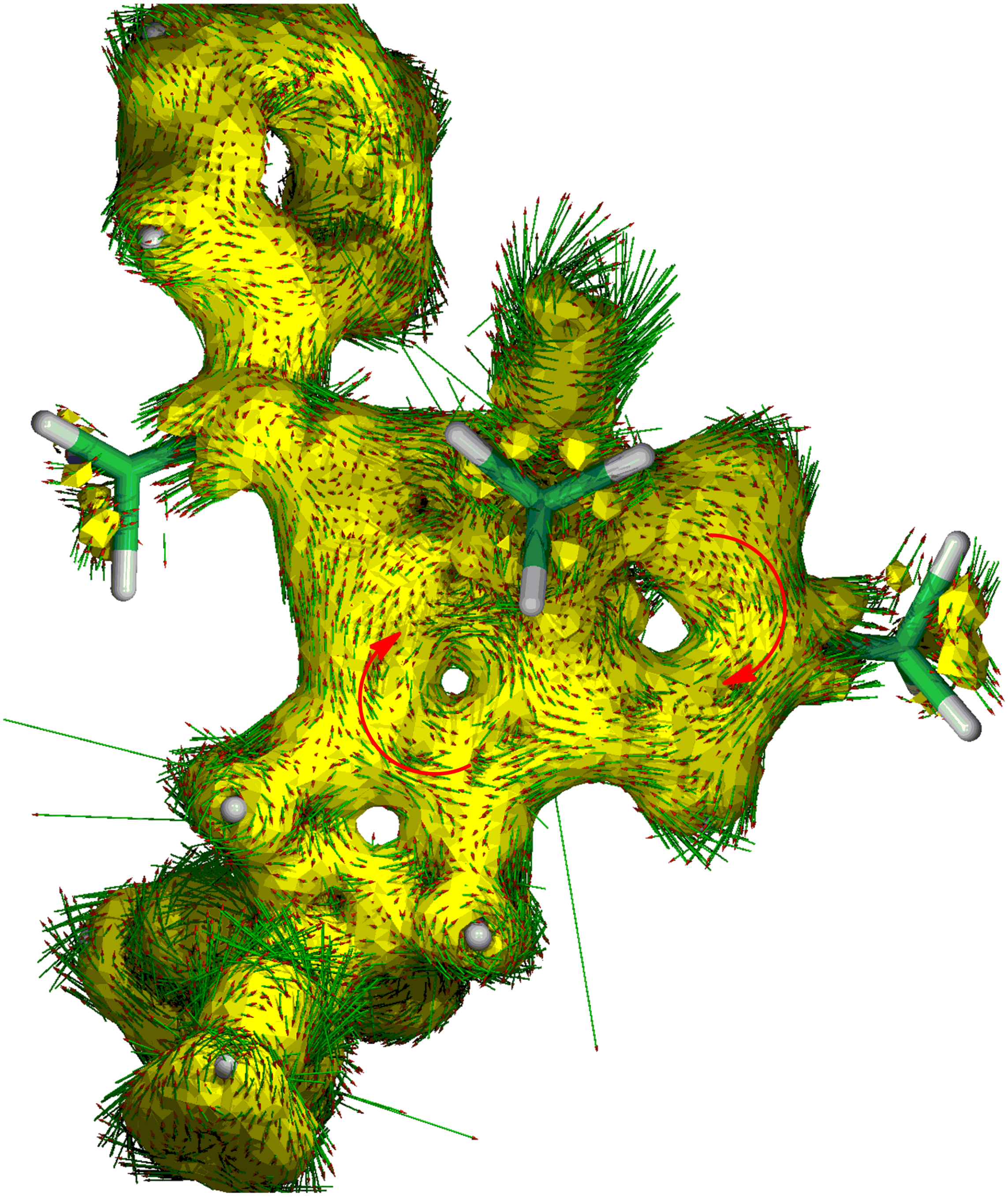

Supplementary Figure 14. AICD plot of the cation moiety of model complex **5′** with an isosurface value of 0.025. The magnetic field vector is orthogonal to the ring plane and points upward (clockwise currents are diatropic).

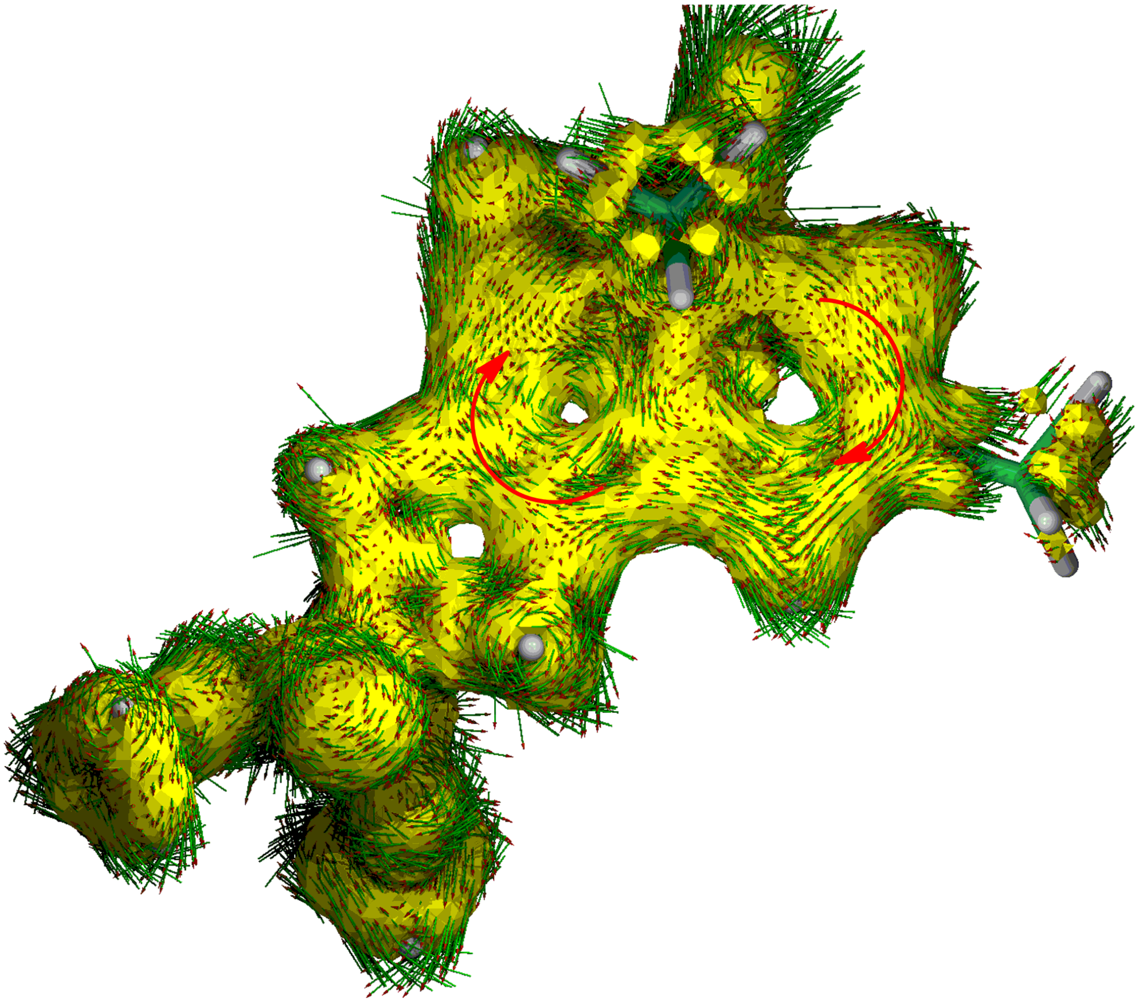

Supplementary Figure 15. AICD plot of the cation moiety of model complex **6′** with an isosurface value of 0.025. The magnetic field vector is orthogonal to the ring plane and points upward (clockwise currents are diatropic).

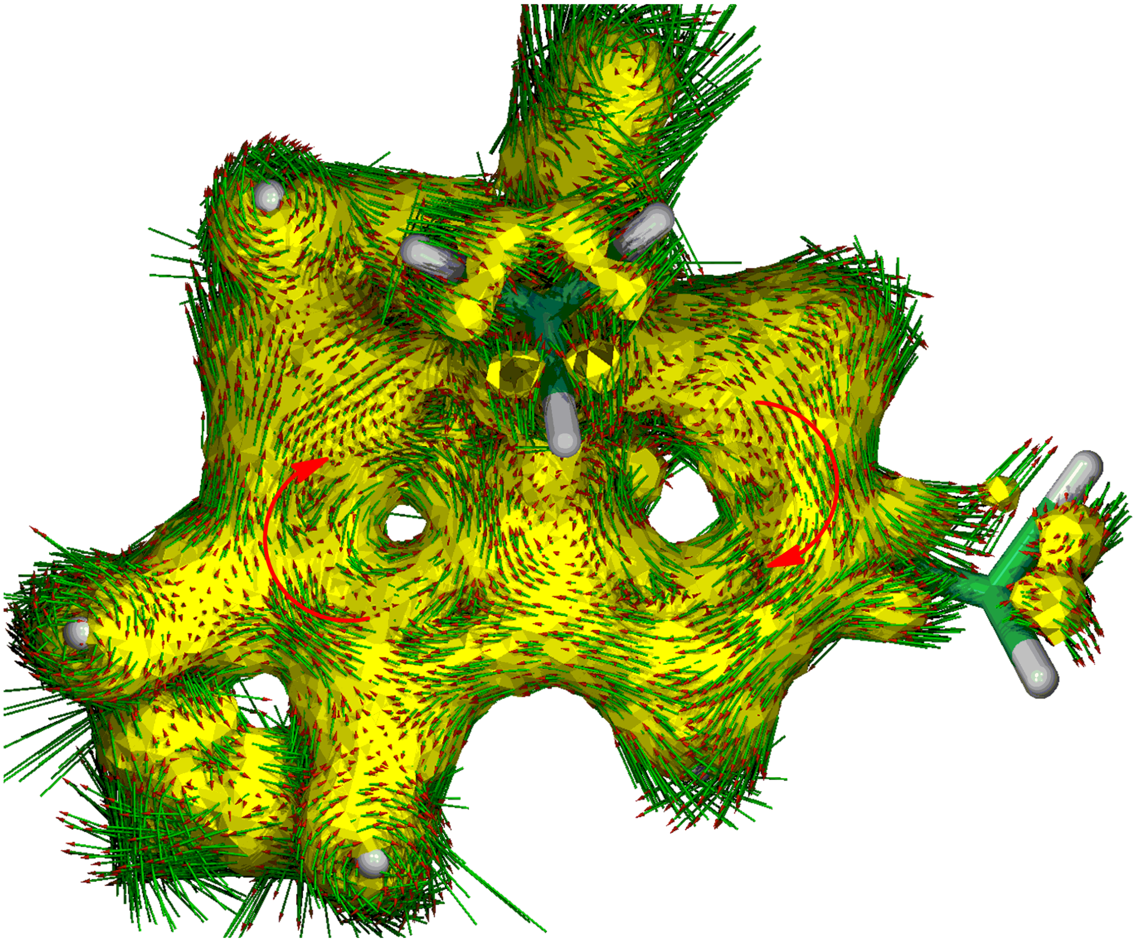

Supplementary Figure 16. AICD plot of the cation moiety of model complex **7′** with an isosurface value of 0.025. The magnetic field vector is orthogonal to the ring plane and points upward (clockwise currents are diatropic).

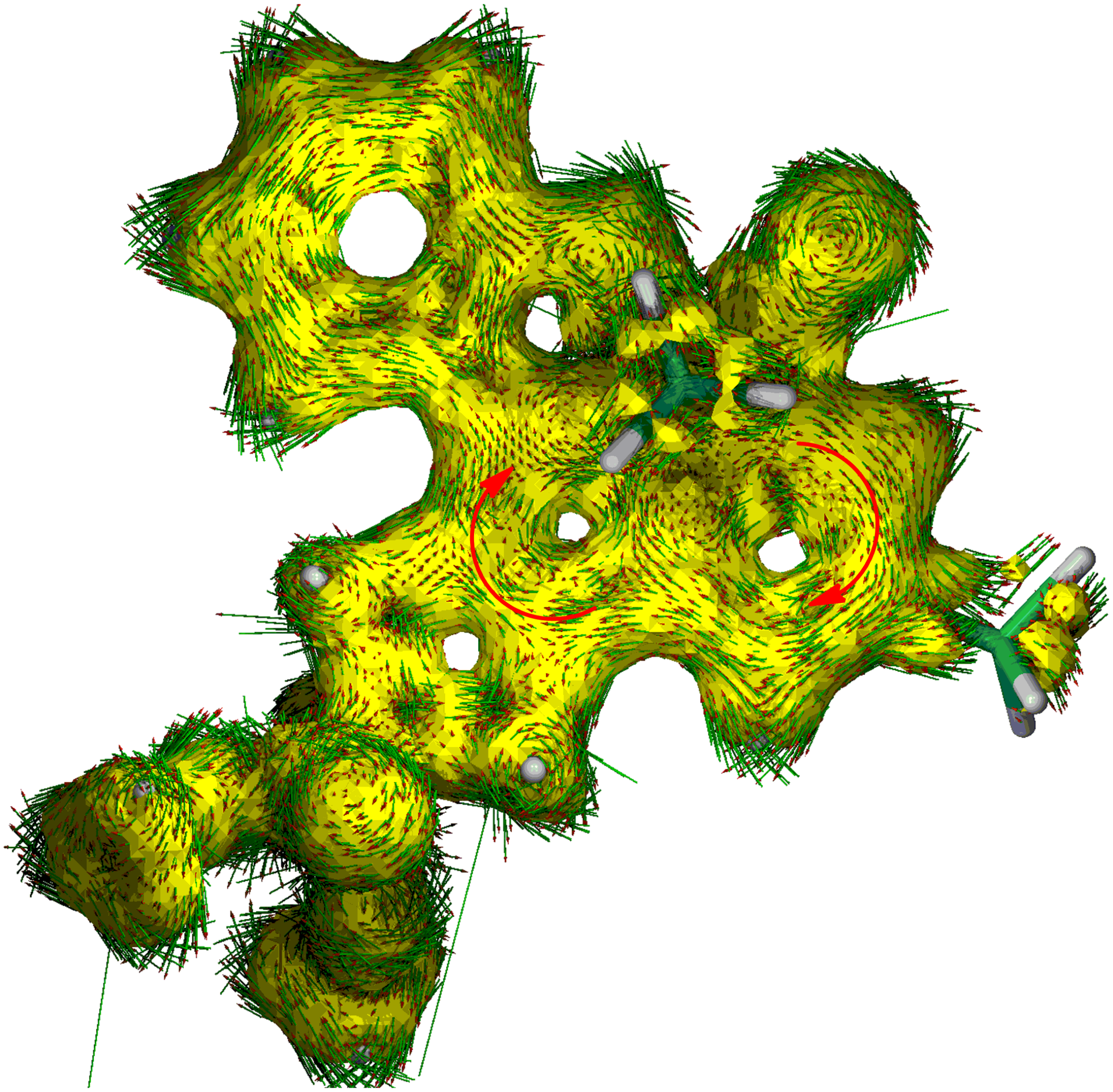

Supplementary Figure 17. AICD plot of the cation moiety of model complex **8′** with an isosurface value of 0.025. The magnetic field vector is orthogonal to the ring plane and points upward (clockwise currents are diatropic).

Proposed Mechanisms for the Formation of Complexes 5–8

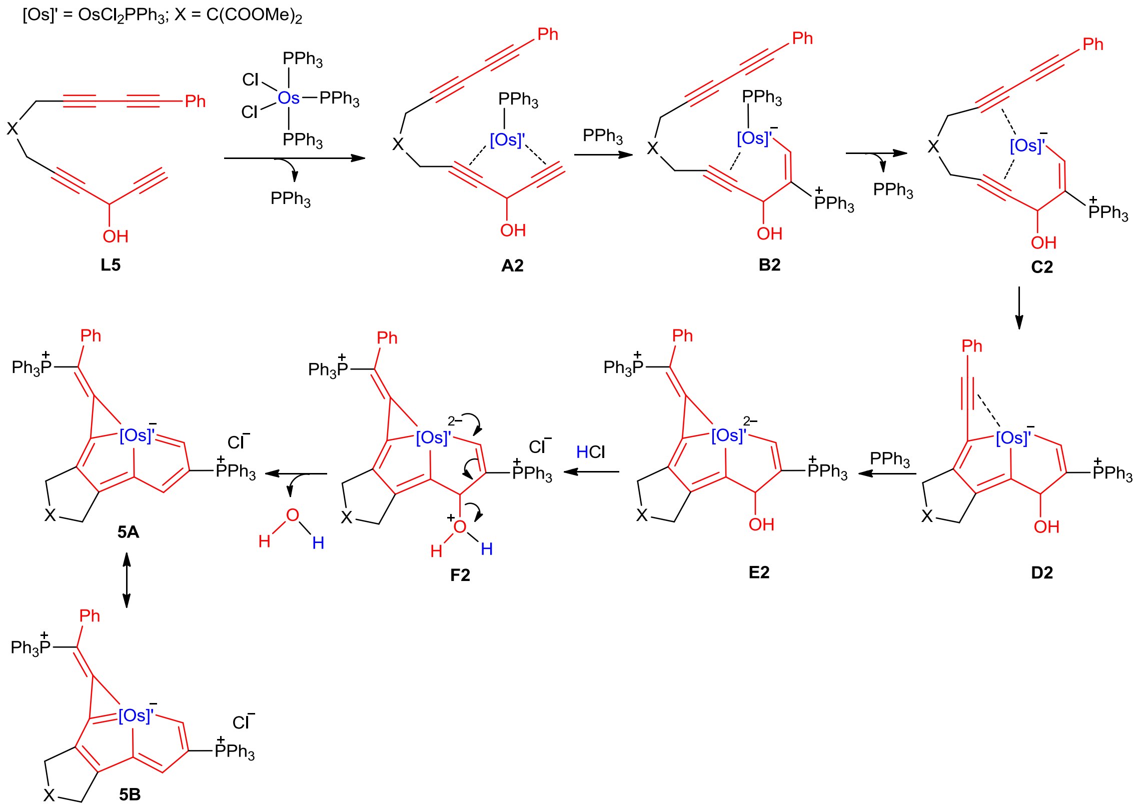

Supplementary Figure 18. Plausible mechanism for the formation of complex **5**. The initial coordination of multiyne **L5** to the metal center and simultaneous dissociation of a PPh_3 ligand leads to the formation of intermediate **A2**. Then, the dissociated PPh_3 undergoes nucleophilic addition to the terminal coordinated C≡C triple bond to form osmium vinyl intermediate **B2**. The subsequent intramolecular ligand substitution of PPh_3 with the other C≡C triple bond may afford **C2**, which could facilitate the [2+2+1] cyclization reaction to furnish bicyclic intermediate **D2**. Another nucleophilic addition of PPh_3 to the remaining coordinated C≡C triple bond would result in the formation of tricyclic intermediate **E2**. Finally, the aromatization of **E2** would produce the aromatic product **5**.

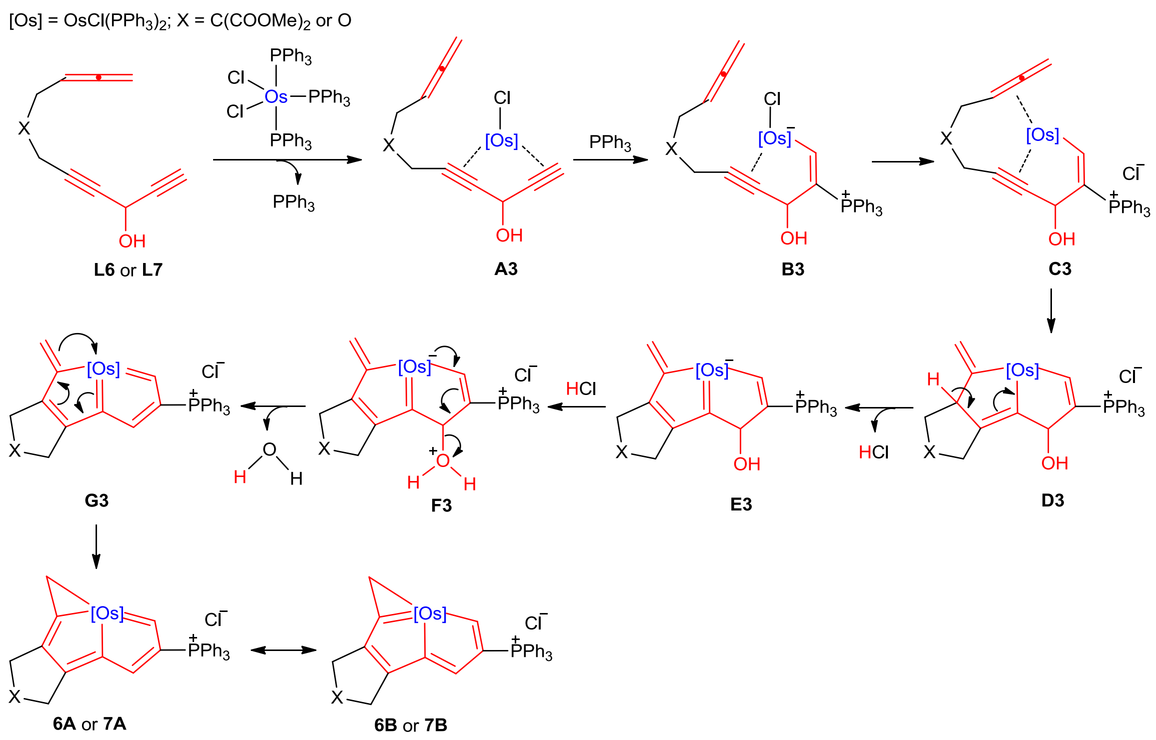

Supplementary Figure 19. Plausible mechanism for the formation of complexes **6** and **7**. The initial coordination of multiyne **L6** or **L7** to the metal center and simultaneous dissociation of a PPh_3 ligand leads to the formation of intermediate **A3**. Then, the dissociated PPh_3 undergoes nucleophilic addition to the terminal coordinated C≡C triple bond to form osmium vinyl intermediate **B3**. The subsequent intramolecular ligand substitution of Cl with the internal C=C double bond may afford **C3**, which could facilitate the [2+2+1] cyclization reaction to furnish bicyclic intermediate **D3**. Elimination of HCl from 16-valence-electron osmium species **D3** can generate 18-valence-electron osmium intermediate **E3**, followed by an acid-promoted aromatization to yield the final aromatic product **6** or **7**.

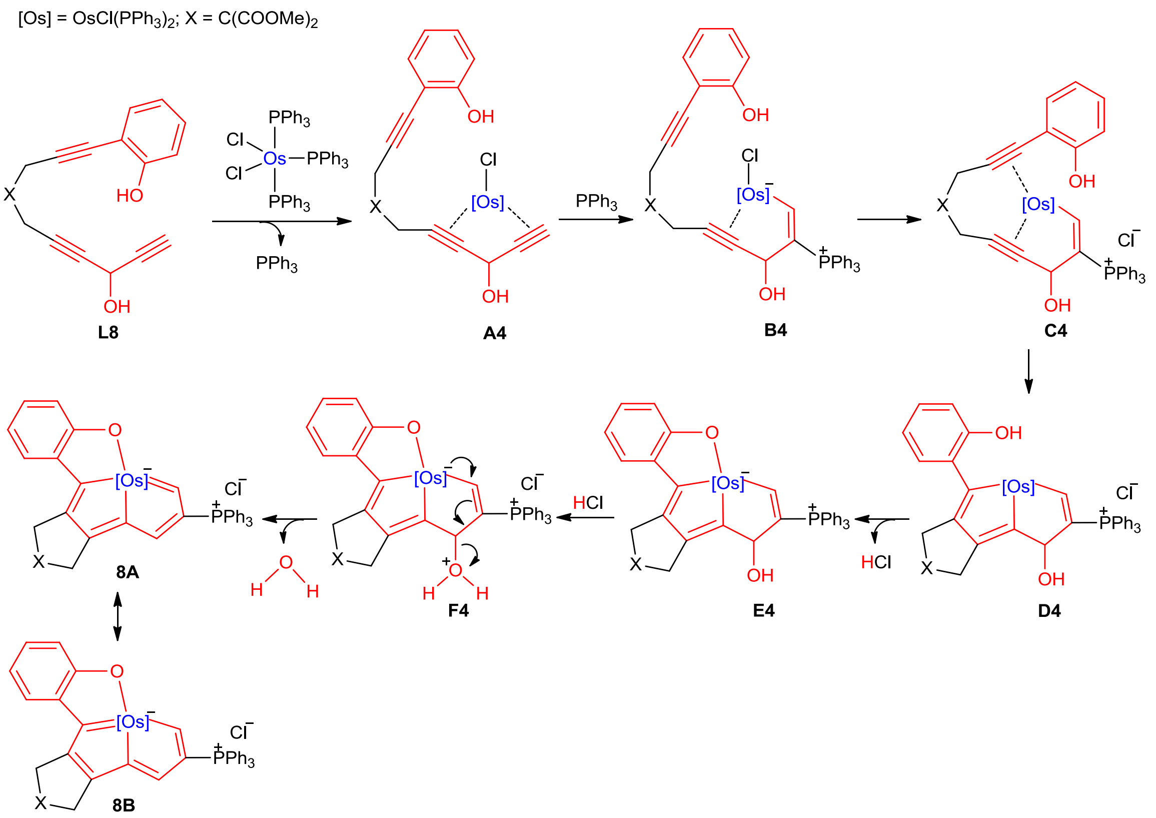

Supplementary Figure 20. Plausible mechanism for the formation of complex **8**. The initial coordination of multiyne **L8** to the metal center and simultaneous dissociation of a PPh_3 ligand leads to the formation of intermediate **A4**. Then, the dissociated PPh_3 undergoes nucleophilic addition to the terminal coordinated C≡C triple bond to form osmium vinyl intermediate **B4**. The subsequent intramolecular ligand substitution of Cl with the other C≡C triple double bond may afford **C4**, which could facilitate the [2+2+1] cyclization reaction to furnish bicyclic intermediate **D4**. Coordination of the phenolic hydroxyl to the metal center and simultaneous elimination of HCl form tricyclic intermediate **E4**, followed by an acid-promoted aromatization to yield the final aromatic product **8**.

In-situ Monitoring Spectra for the Reaction of L4 with $OsCl_2(PPh_3)_3$

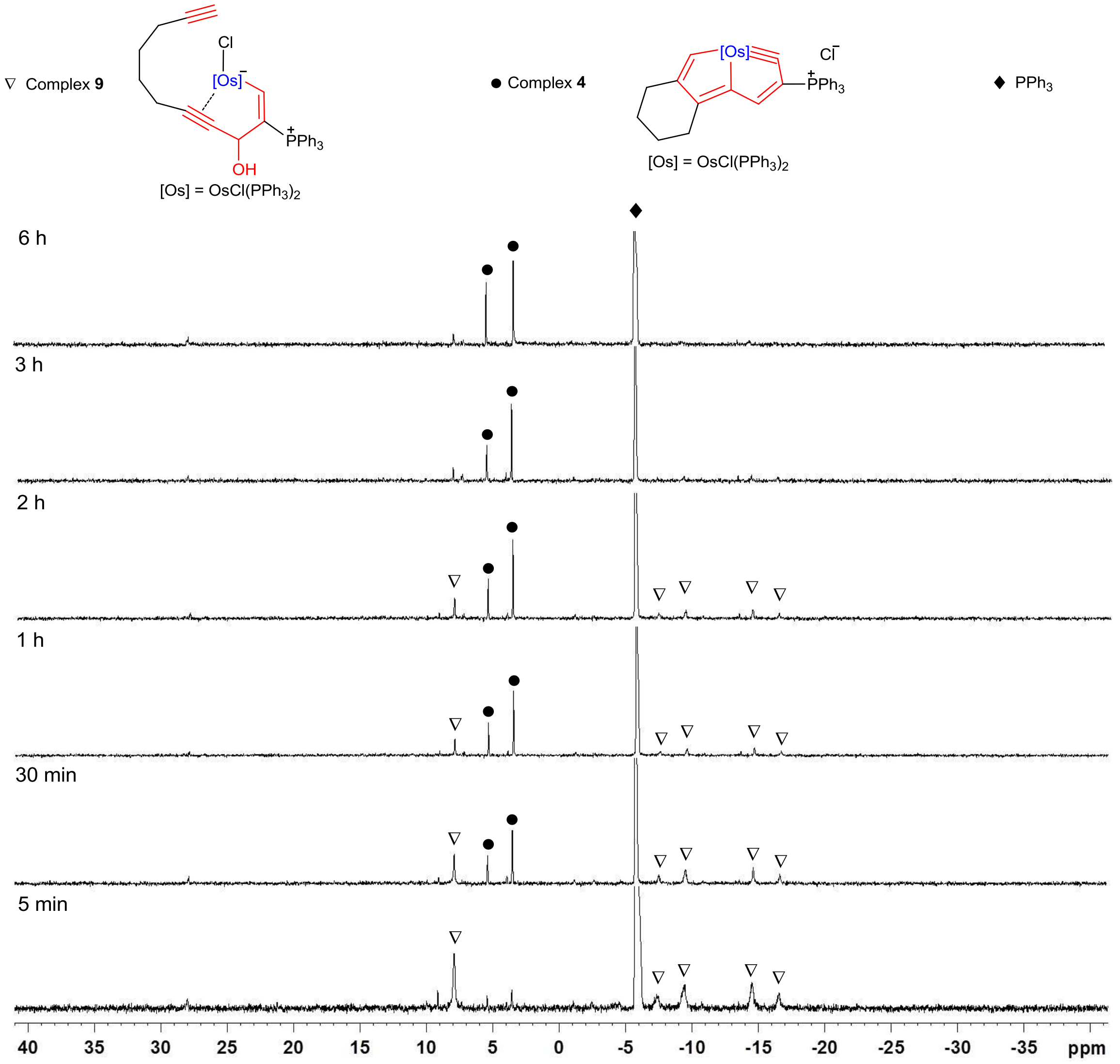

Supplementary Figure 21. In-situ ^{31}P NMR spectra for the reaction of **L4** with $OsCl_2(PPh_3)_3$ in a mixture of dichloromethane and tert-butanol (3:1 v/v) at RT.

HRMS Spectra

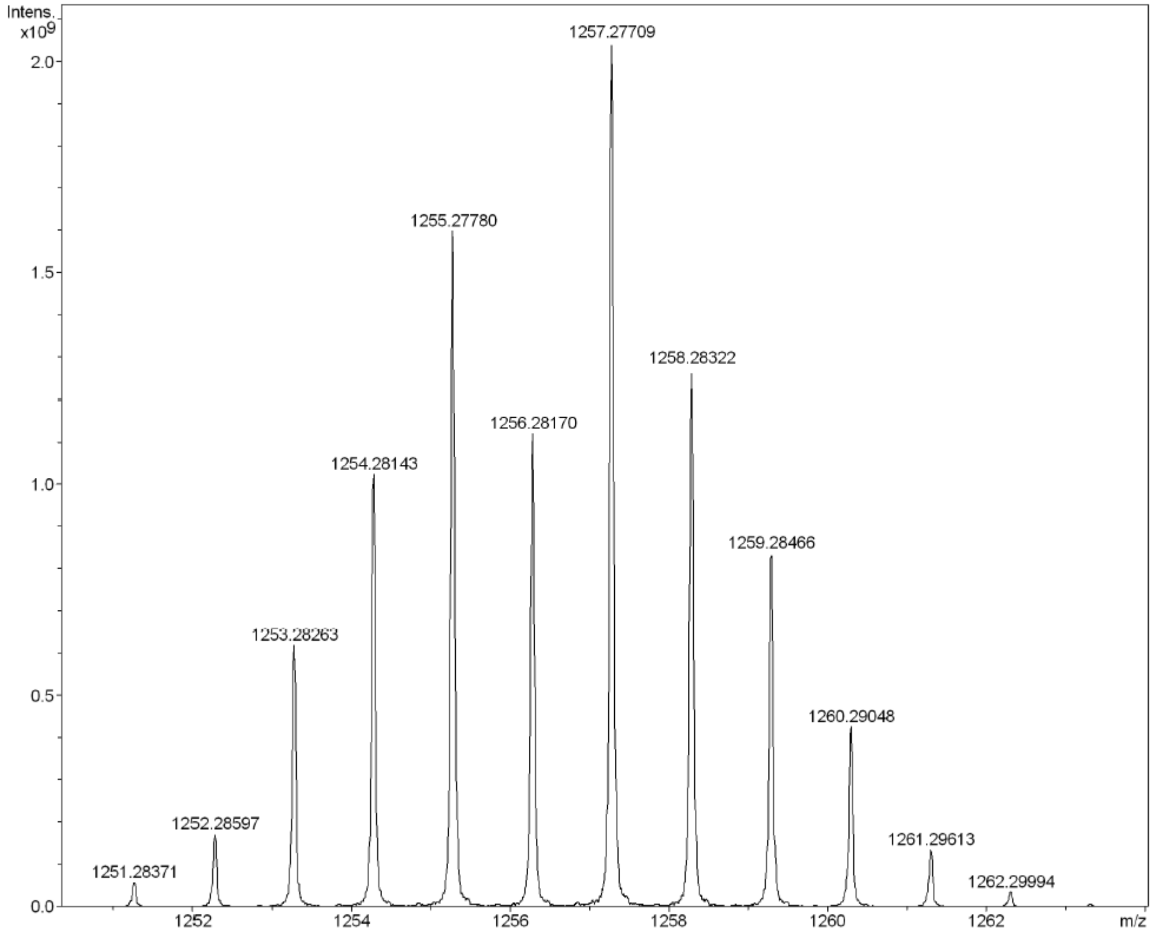

Supplementary Figure 22. Positive-ion ESI-MS spectrum of [**1**]$^+$ [$C_{68}H_{57}ClO_4P_3Os$]$^+$ measured in methanol.

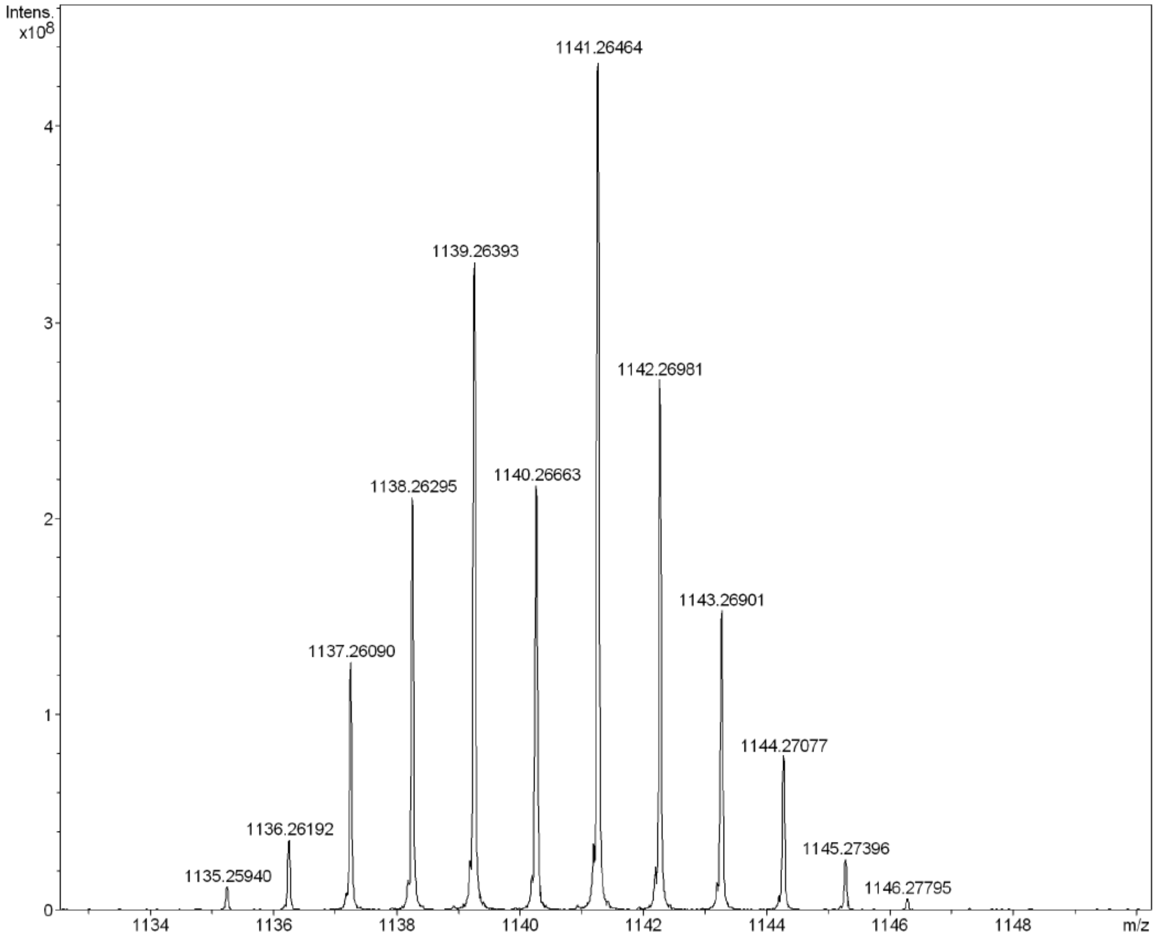

Supplementary Figure 23. Positive-ion ESI-MS spectrum of [**2**]$^+$ [$C_{64}H_{53}ClP_3Os$]$^+$ measured in methanol.

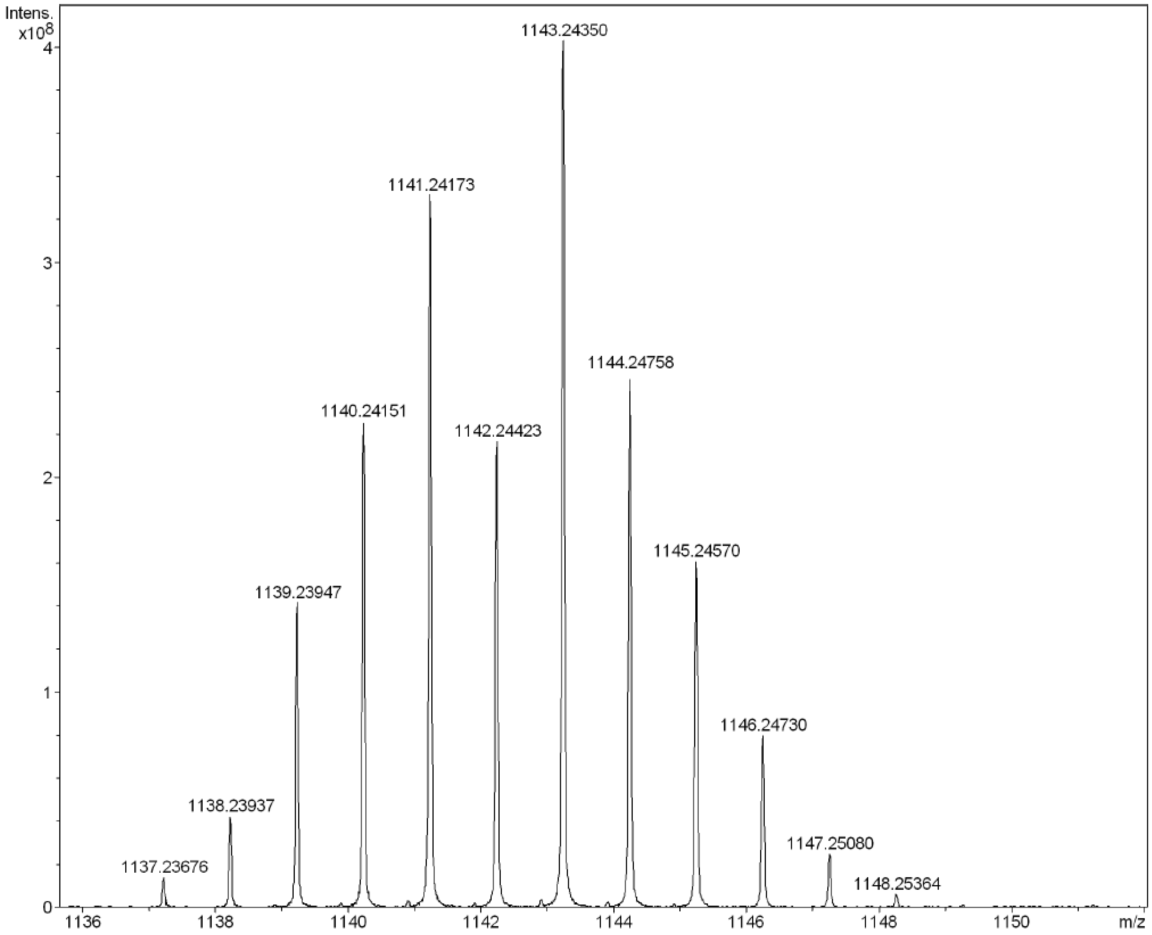

Supplementary Figure 24. Positive-ion ESI-MS spectrum of [**3**]$^+$ [$C_{63}H_{51}OClP_3Os$]$^+$ measured in methanol.

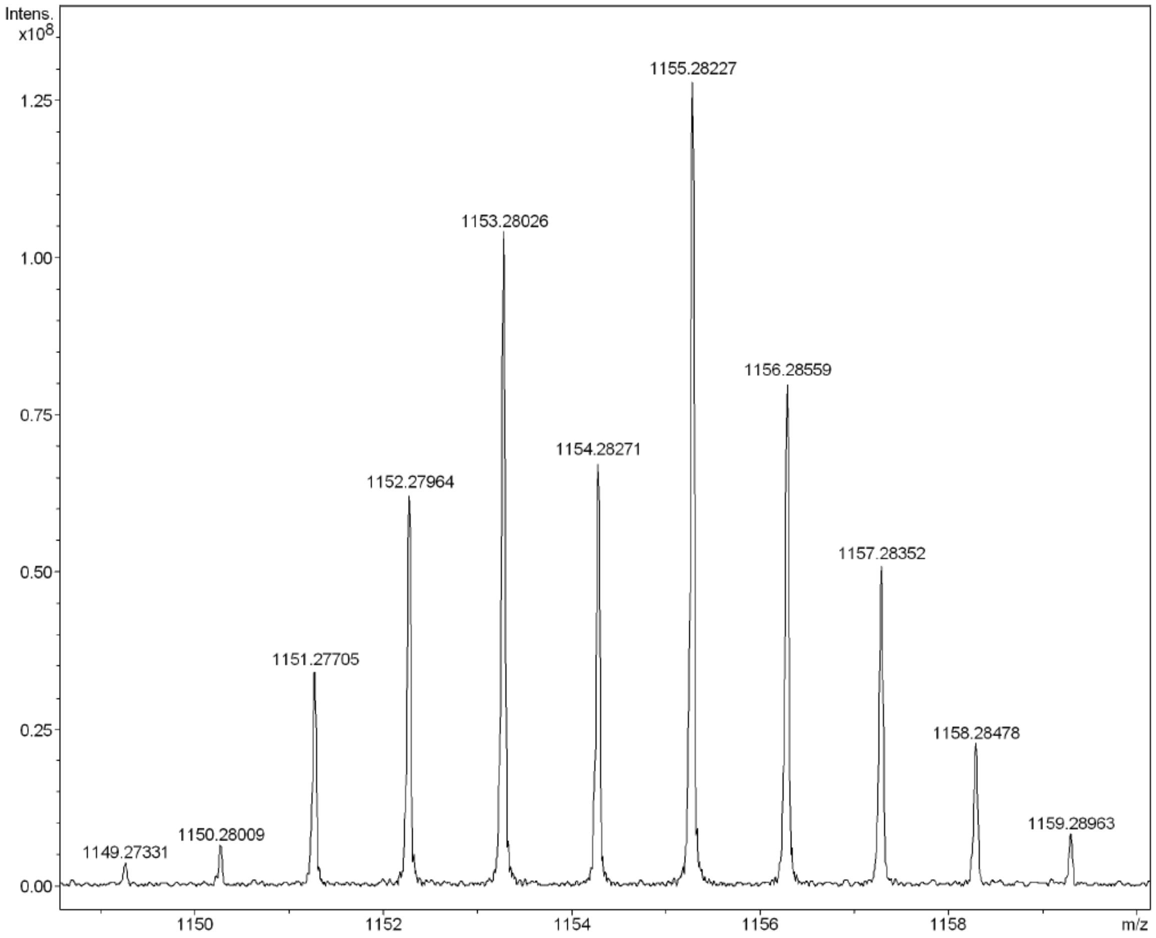

Supplementary Figure 25. Positive-ion ESI-MS spectrum of [**4**]$^+$ [$C_{65}H_{55}ClP_3Os$]$^+$ measured in methanol.

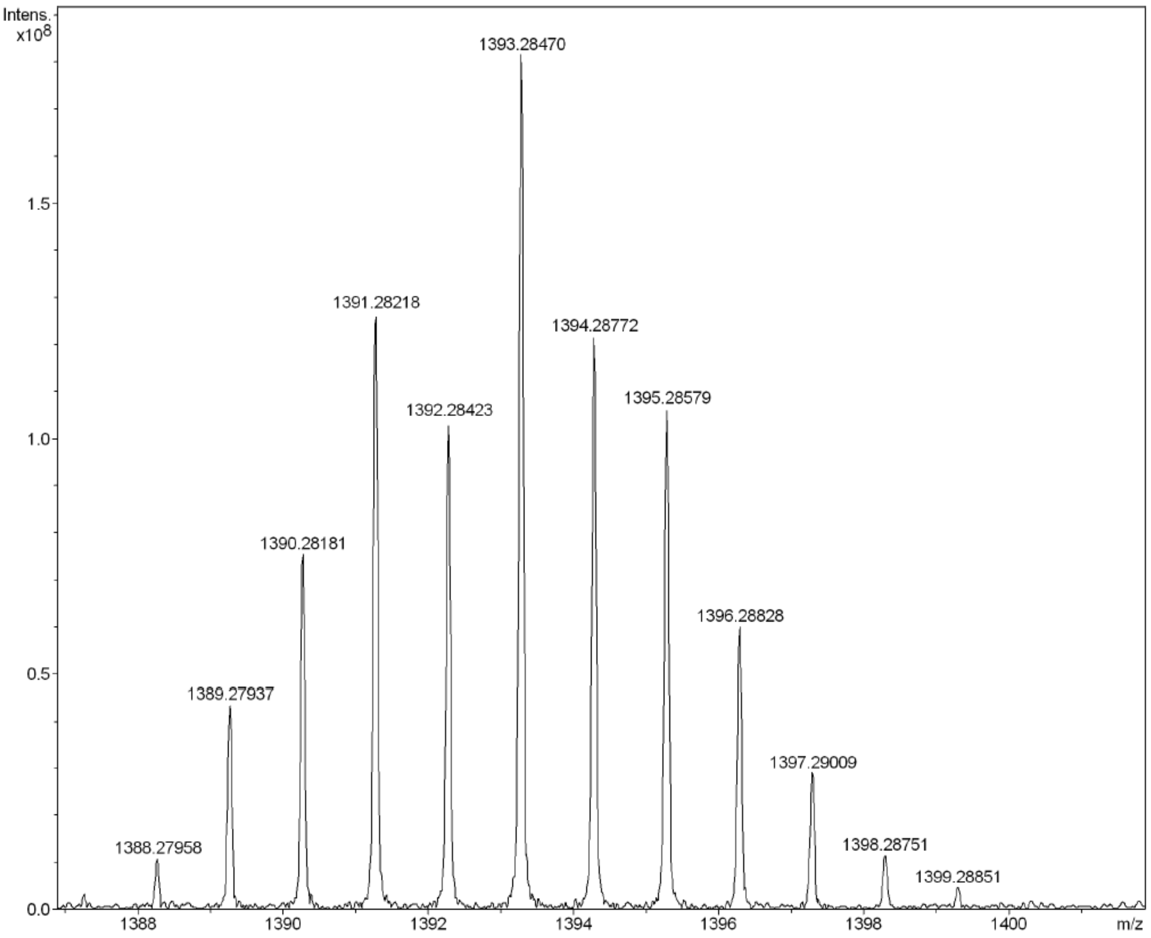

Supplementary Figure 26. Positive-ion ESI-MS spectrum of [**5**]$^+$ [$C_{76}H_{62}Cl_2O_4P_3Os$]$^+$ measured in methanol.

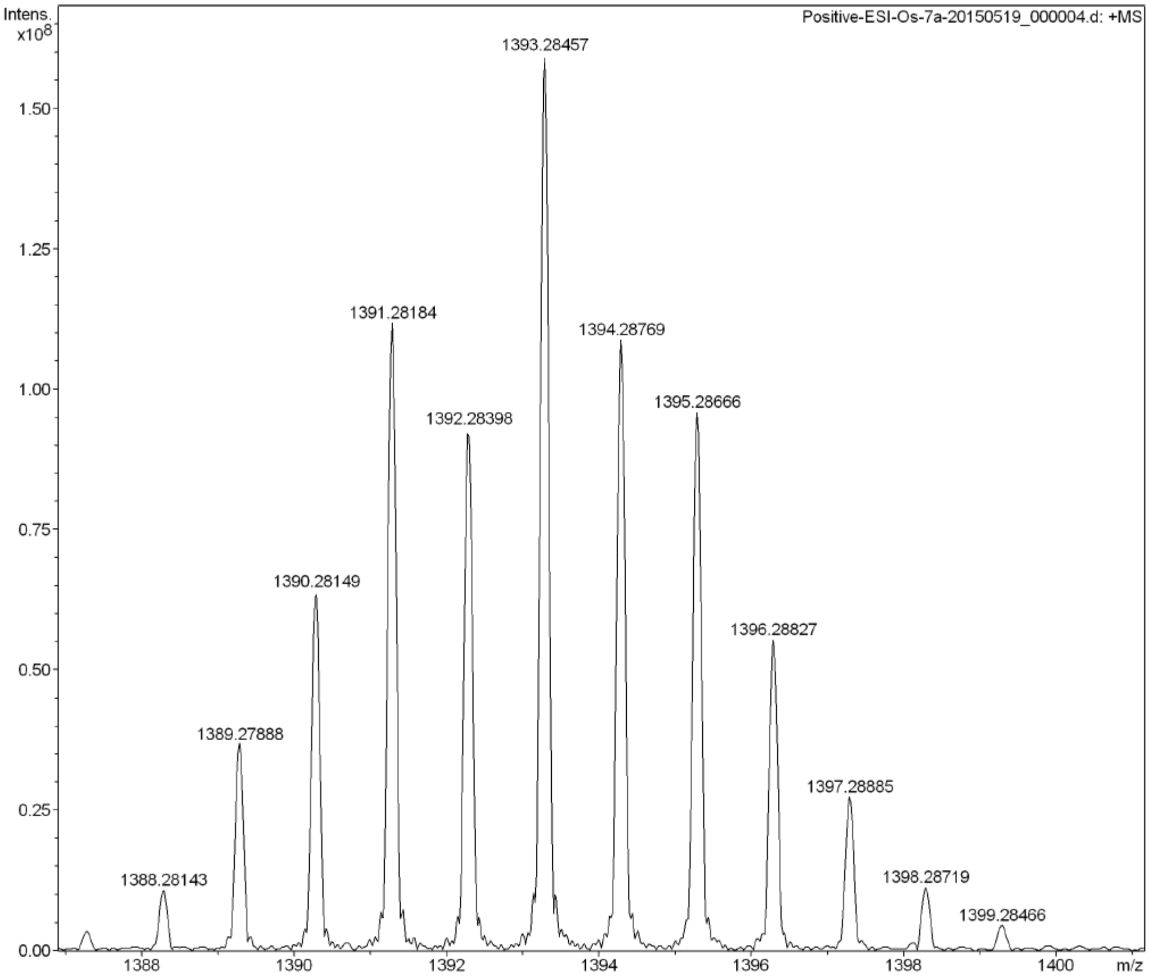

Supplementary Figure 27. Positive-ion ESI-MS spectrum of [**5-PF$_6$**]$^+$ $[C_{76}H_{62}Cl_2O_4P_3Os]^+$ measured in methanol.

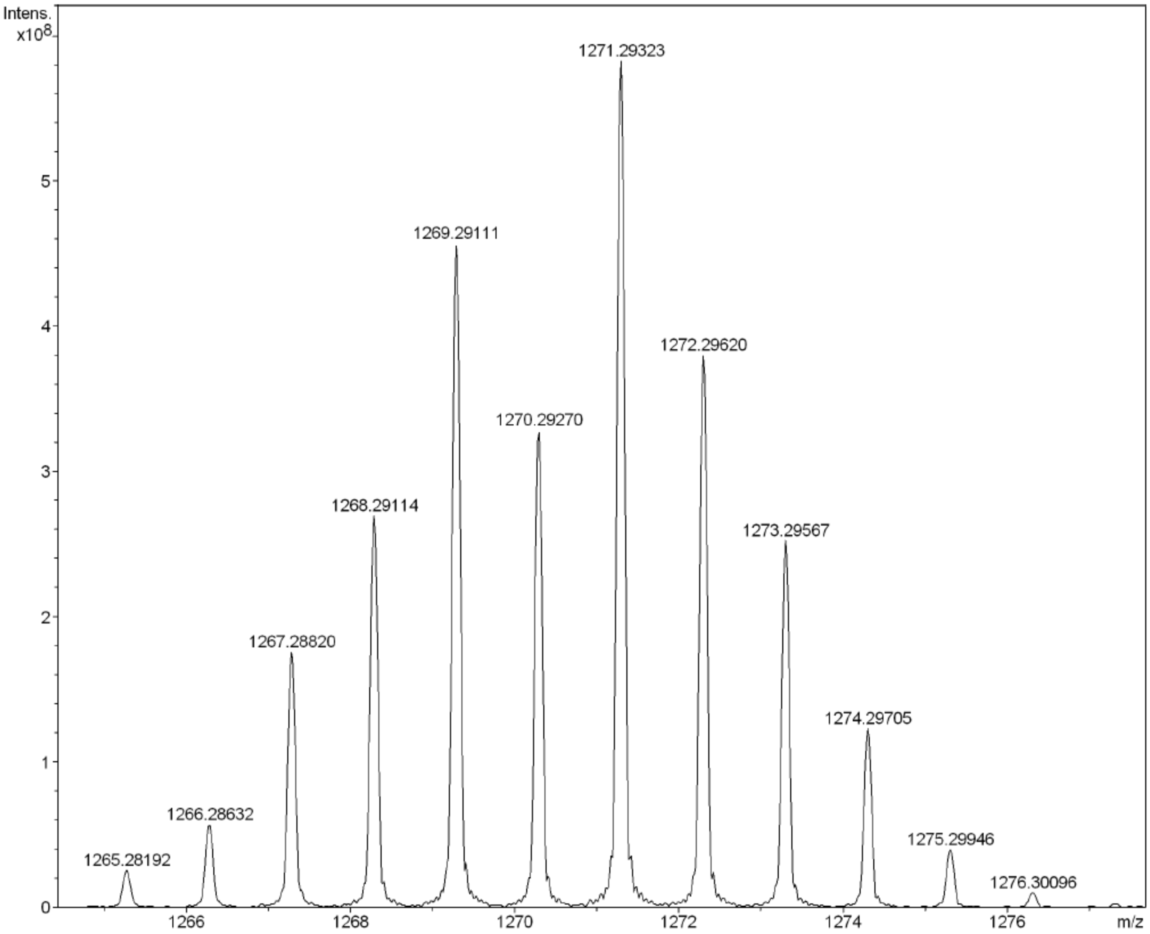

Supplementary Figure 28. Positive-ion ESI-MS spectrum of [**6**]$^+$ [$C_{69}H_{59}ClO_4P_3Os$]$^+$ measured in methanol.

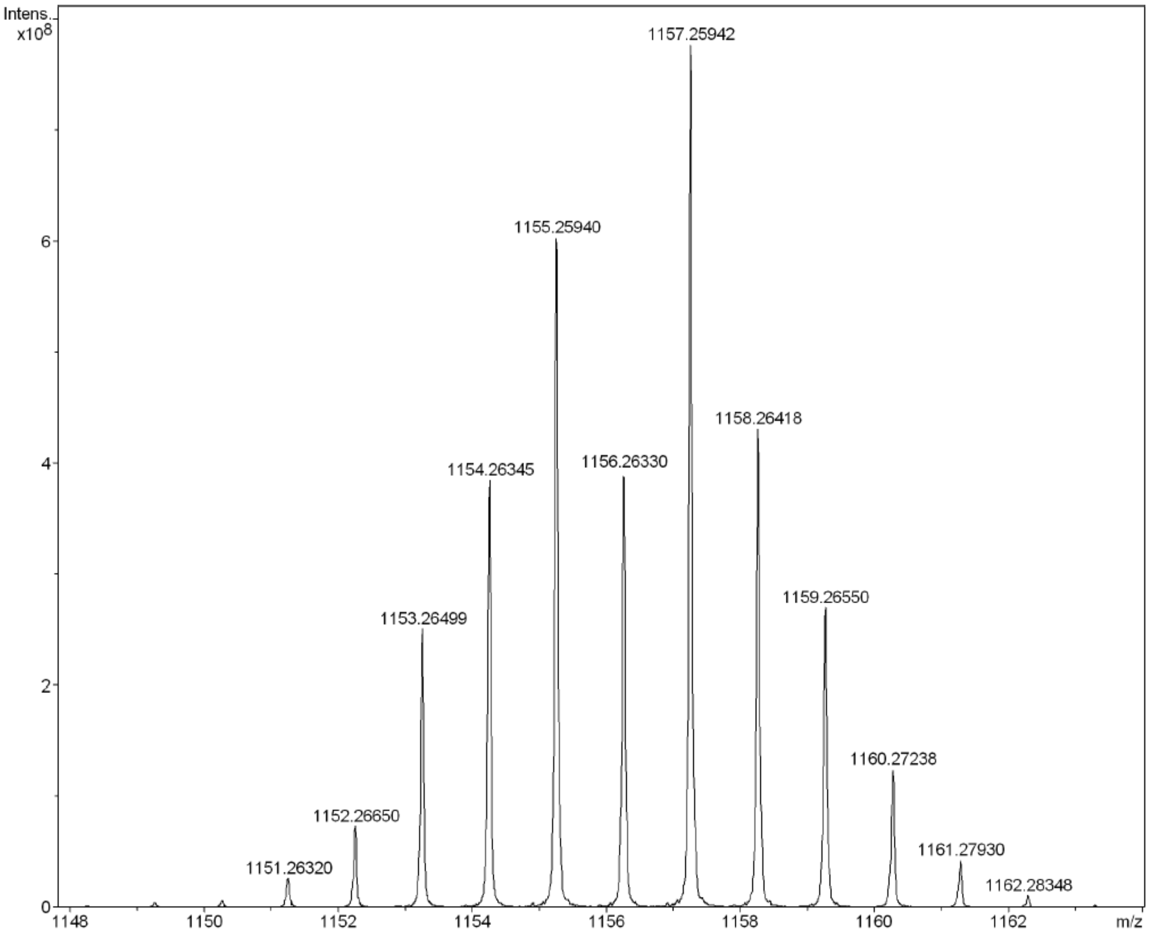

Supplementary Figure 29. Positive-ion ESI-MS spectrum of [**7**]$^+$ [$C_{64}H_{53}ClOP_3Os$]$^+$ measured in methanol.

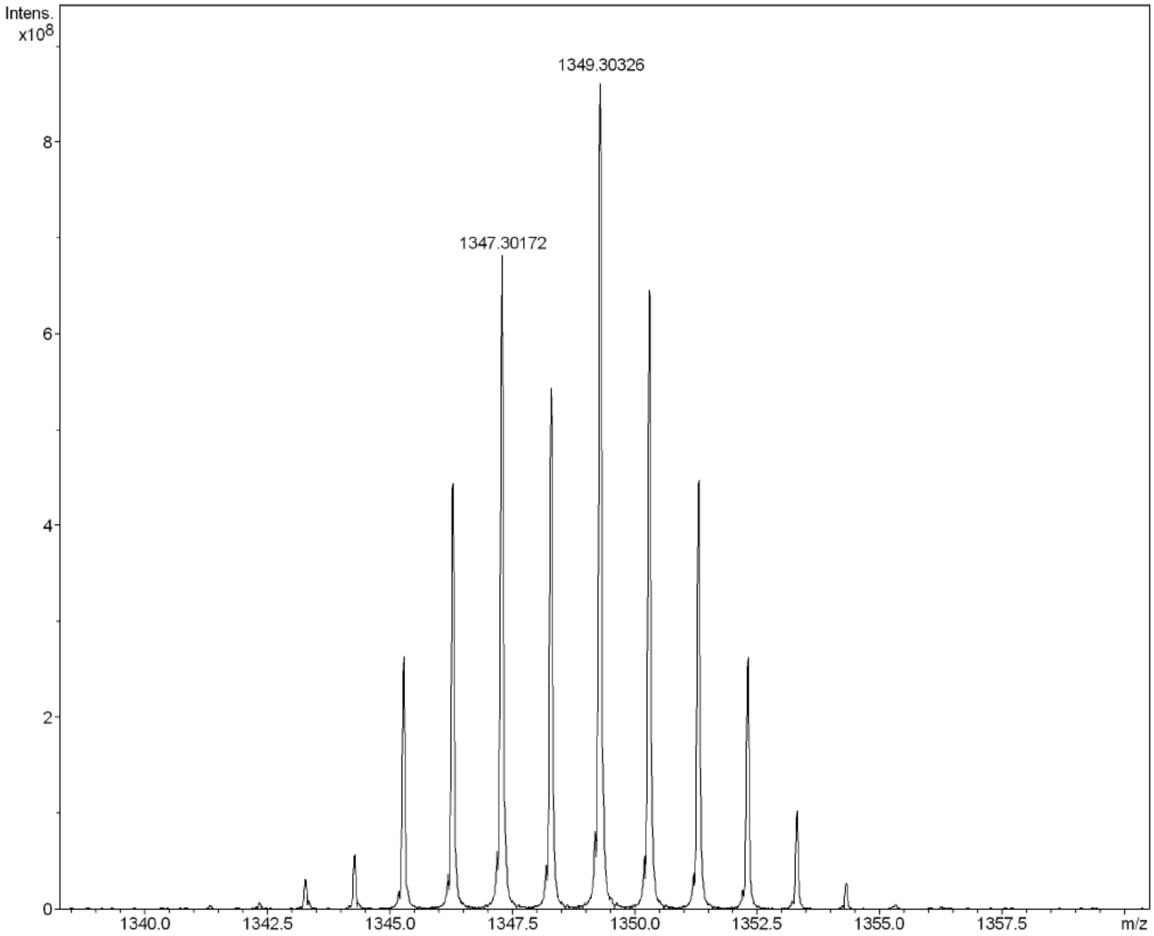

Supplementary Figure 30. Positive-ion ESI-MS spectrum of [**8**]$^+$ [$C_{74}H_{61}ClO_5P_3Os$]$^+$ measured in methanol.

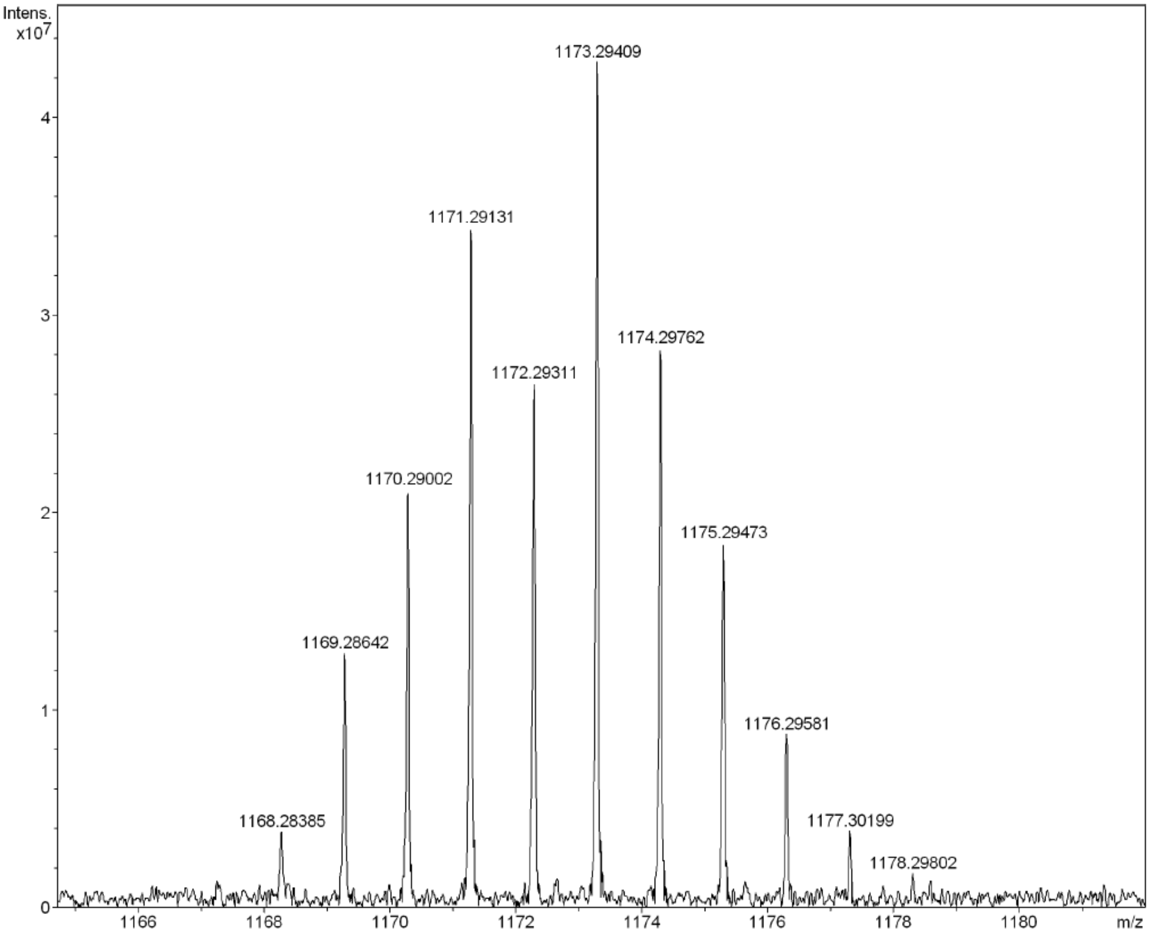

Supplementary Figure 31. Positive-ion ESI-MS spectrum of [**9**–Cl]$^{+}$ [$C_{65}H_{57}ClOP_3Os$]$^{+}$ measured in dichloromethane.

NMR Spectra

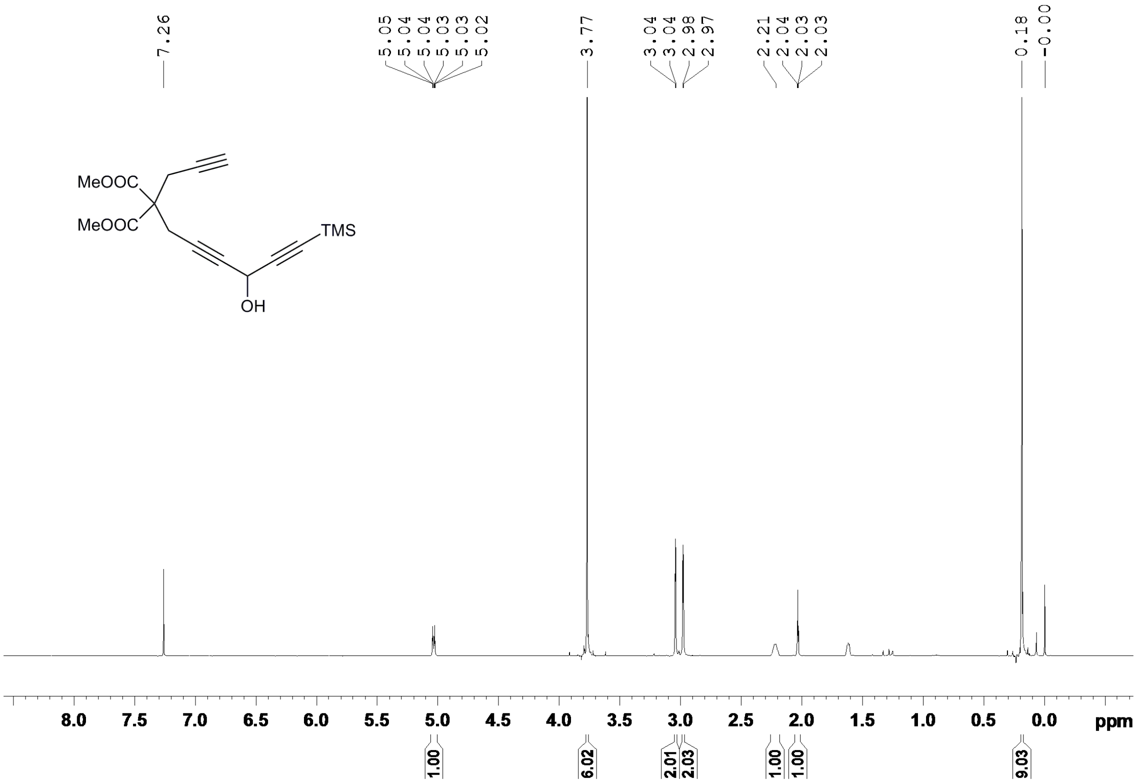

Supplementary Figure 32. ^{1}H NMR spectrum (500.2 MHz, $CDCl_3$) of compound **S1** at room temperature.

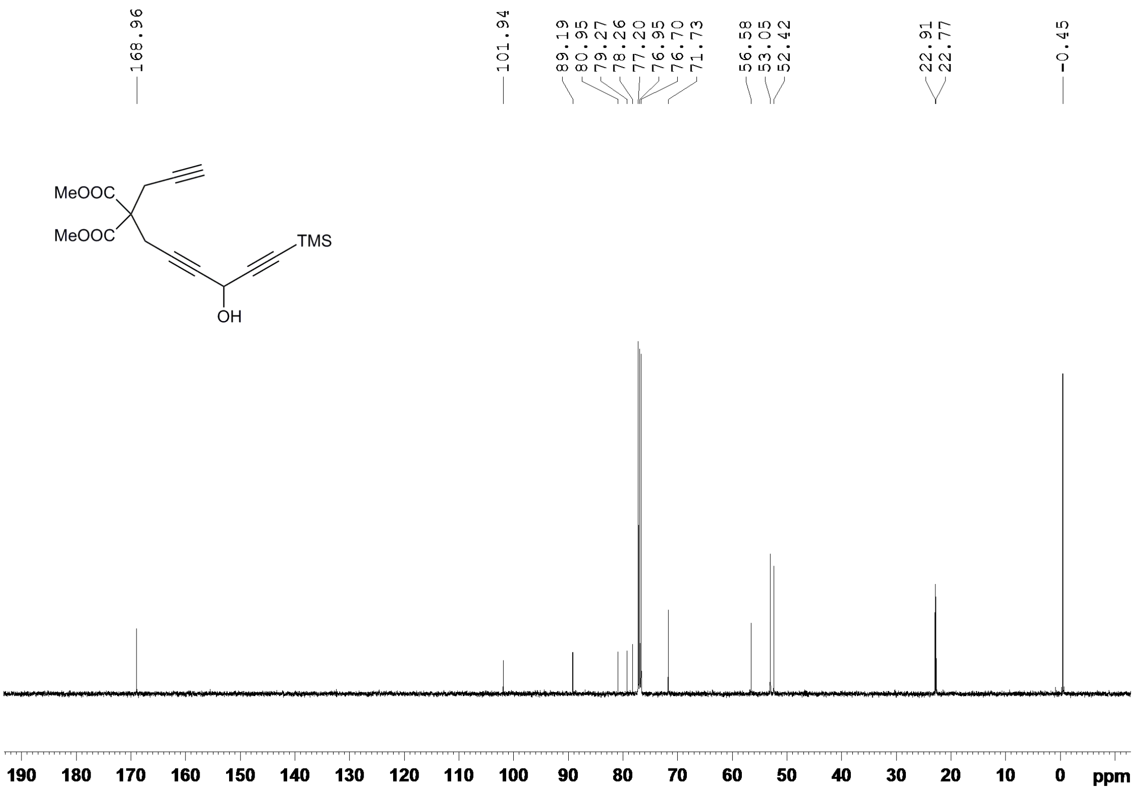

Supplementary Figure 33. ^{13}C NMR spectrum (125.8 MHz, $CDCl_3$) of compound **S1** at room temperature.

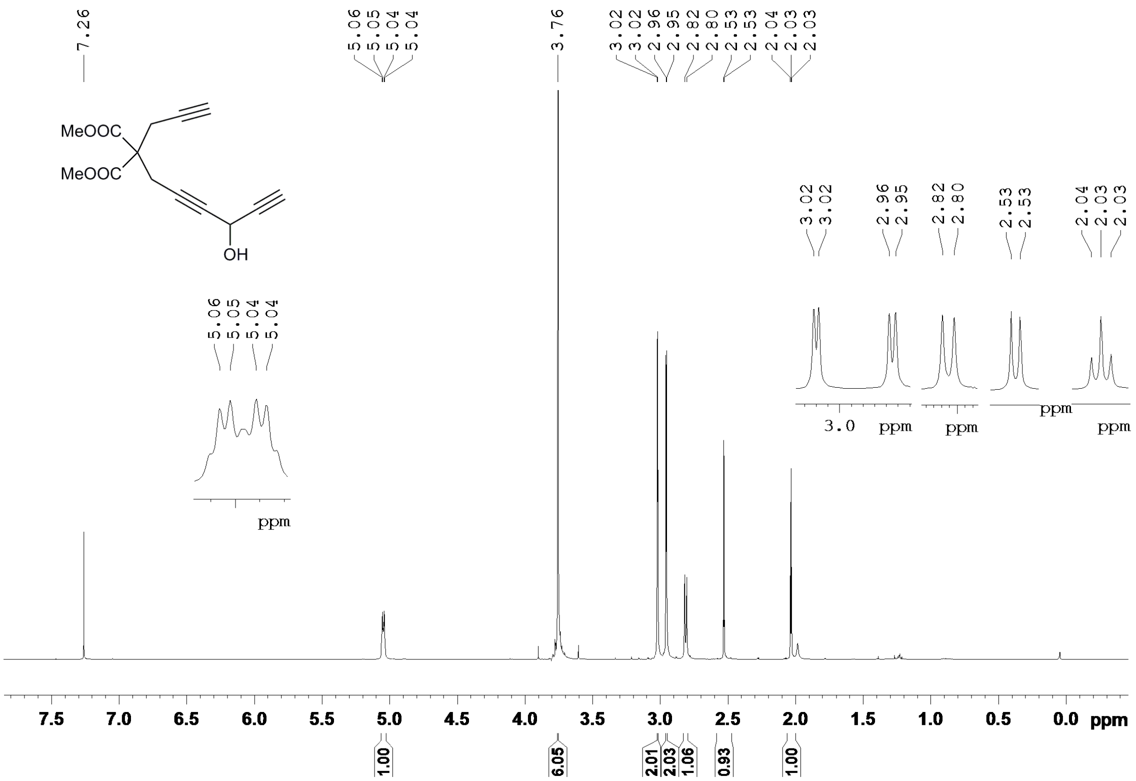

Supplementary Figure 34. ^{1}H NMR spectrum (500.2 MHz, $CDCl_3$) of compound **L1** at room temperature.

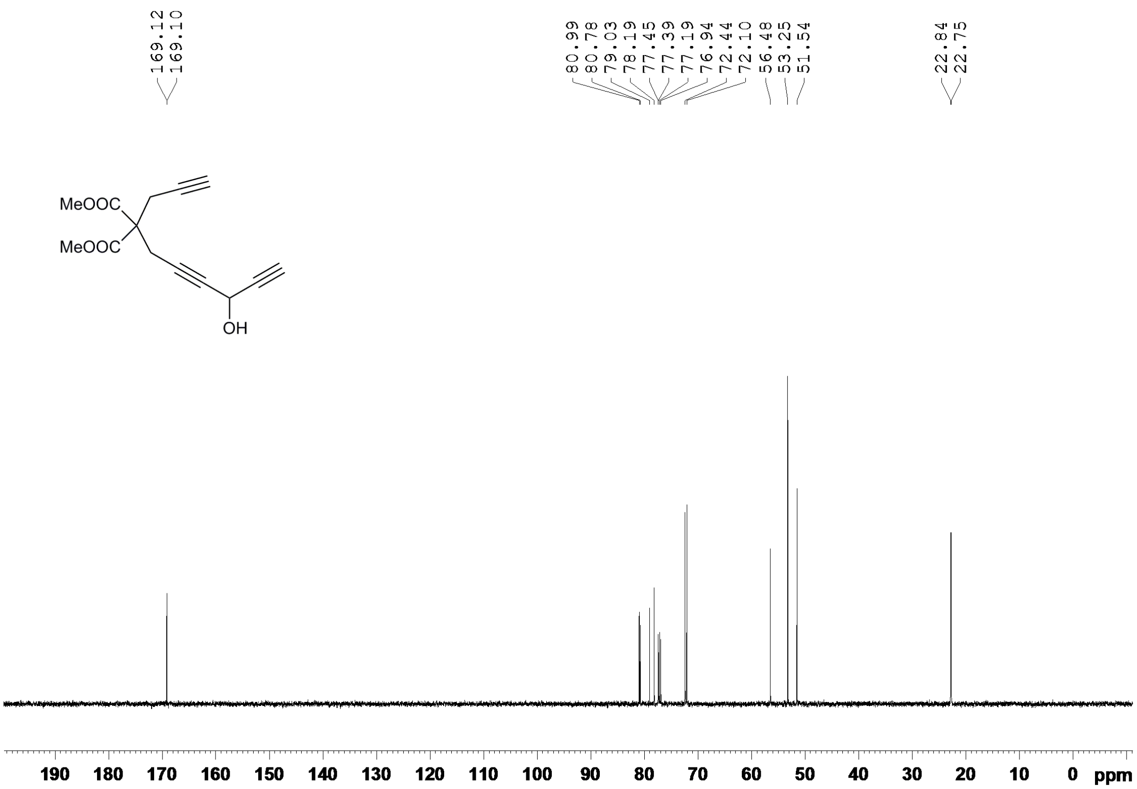

Supplementary Figure 35. ^{13}C NMR spectrum (125.8 MHz, $CDCl_3$) of compound **L1** at room temperature.

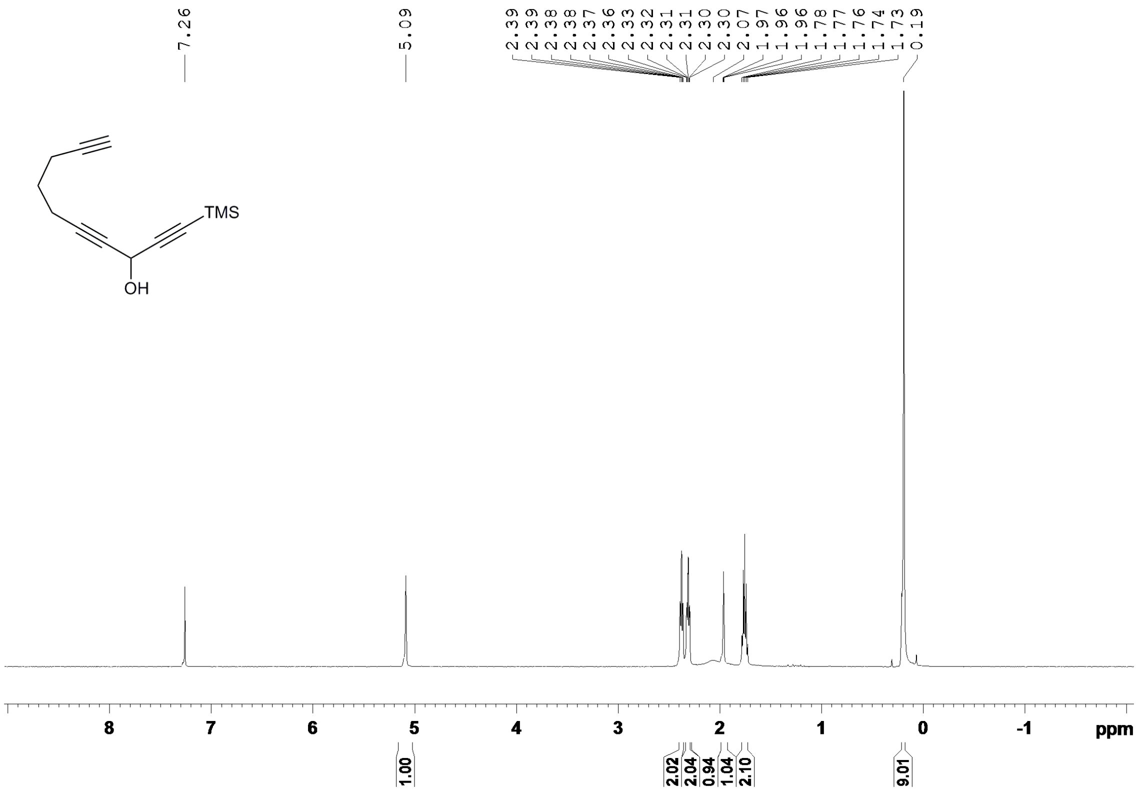

Supplementary Figure 36. ^{1}H NMR spectrum (500.2 MHz, $CDCl_3$) of compound **S2** at room temperature.

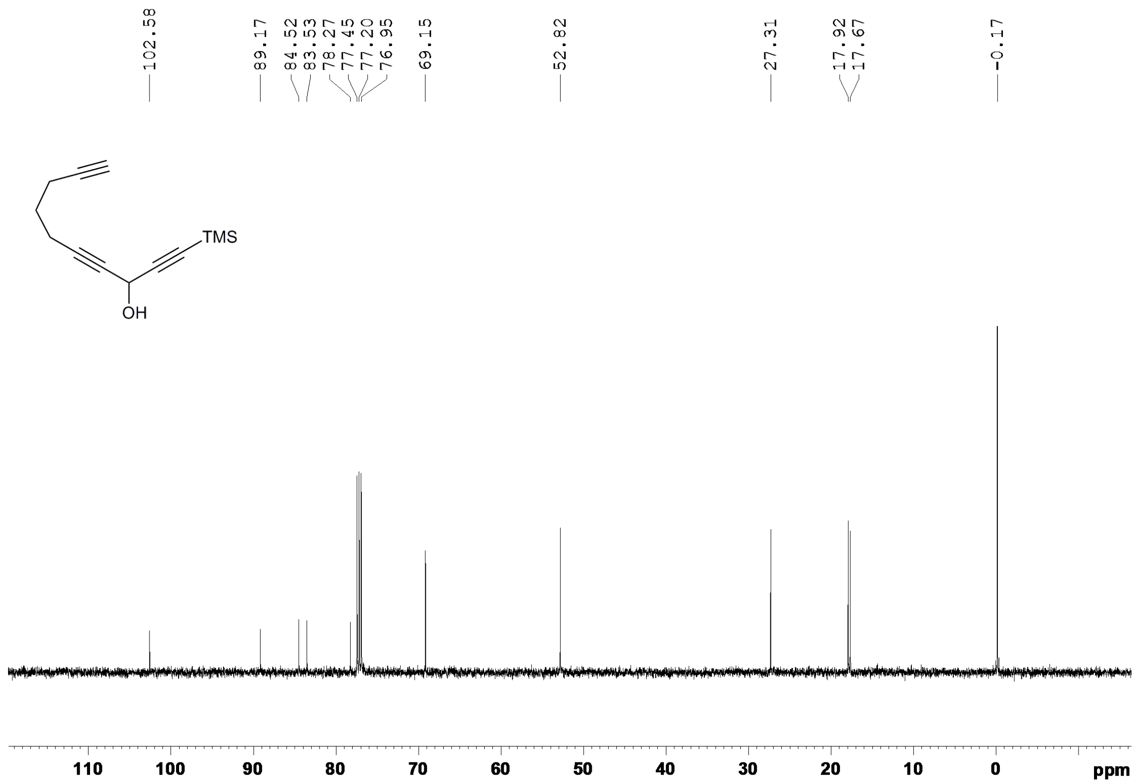

Supplementary Figure 37. ^{13}C NMR spectrum (125.8 MHz, $CDCl_3$) of compound **S2** at room temperature.

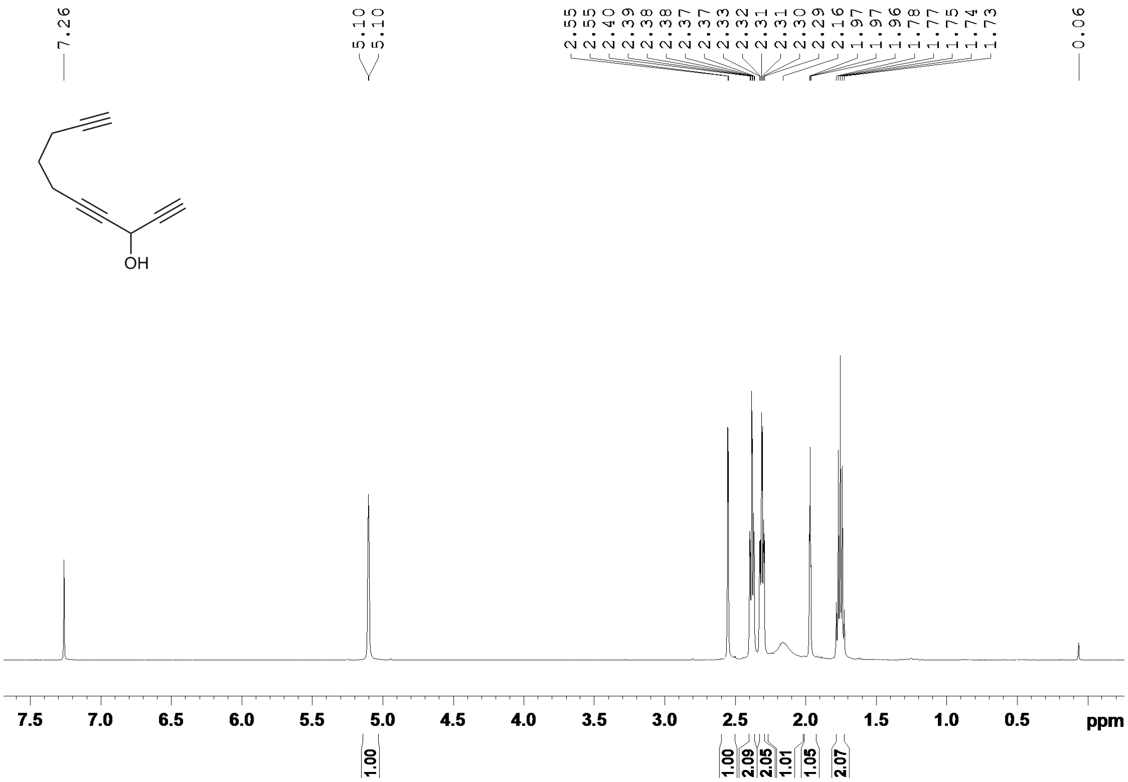

Supplementary Figure 38. ^{1}H NMR spectrum (500.2 MHz, $CDCl_3$) of compound **L2** at room temperature.

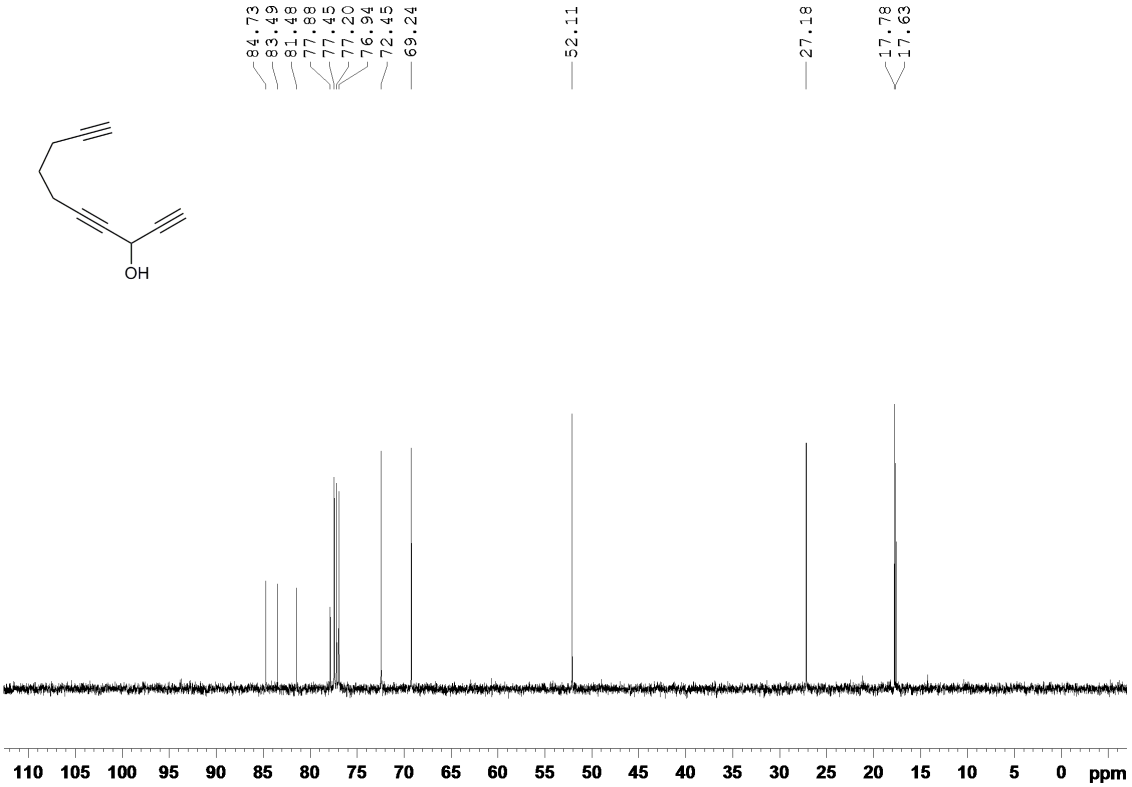

Supplementary Figure 39. ^{13}C NMR spectrum (125.8 MHz, $CDCl_3$) of compound **L2** at room temperature.

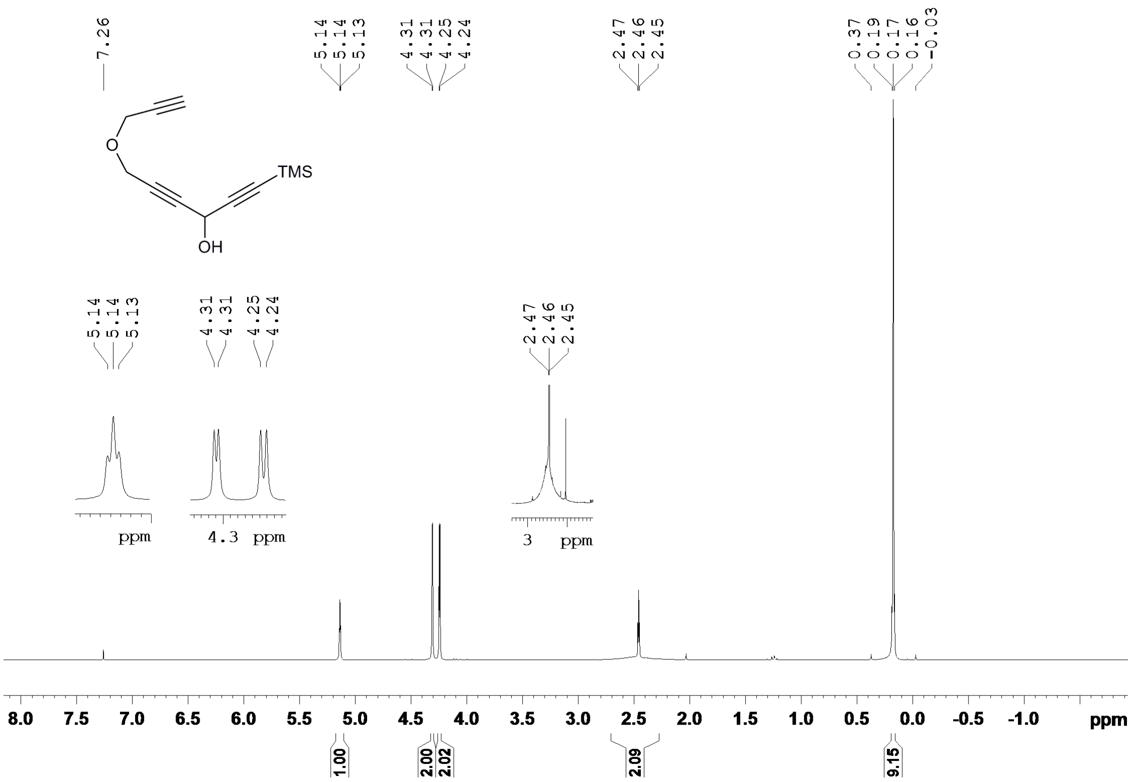

Supplementary Figure 40. ^{1}H NMR spectrum (300.1 MHz, $CDCl_3$) of compound **S3** at room temperature.

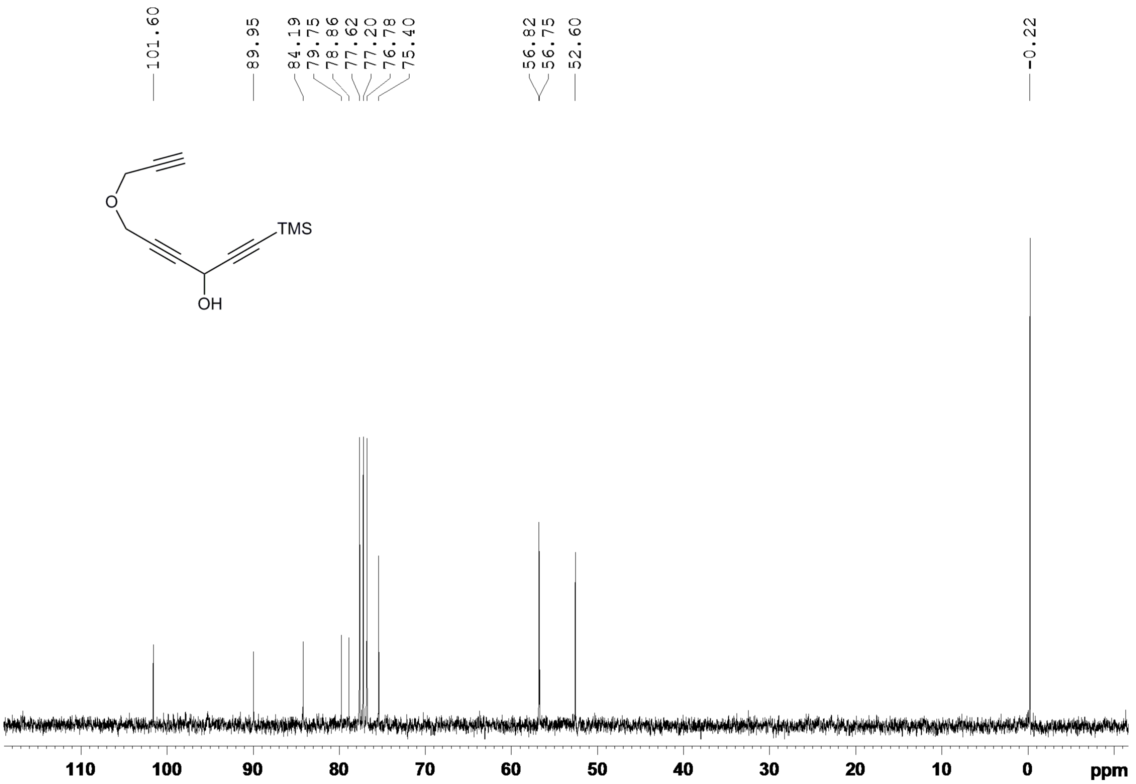

Supplementary Figure 41. ^{13}C NMR spectrum (75.5 MHz, $CDCl_3$) of compound **S3** at room temperature.

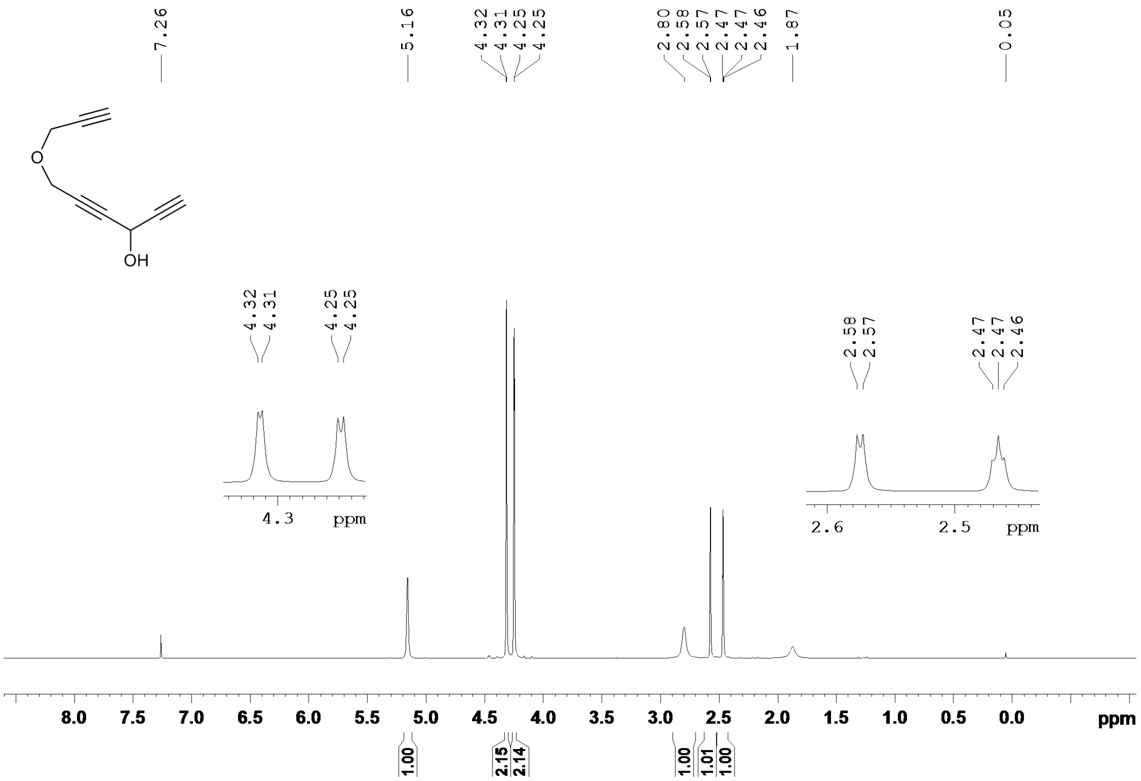

Supplementary Figure 42. ^{1}H NMR spectrum (500.2 MHz, $CDCl_3$) of compound **L3** at room temperature.

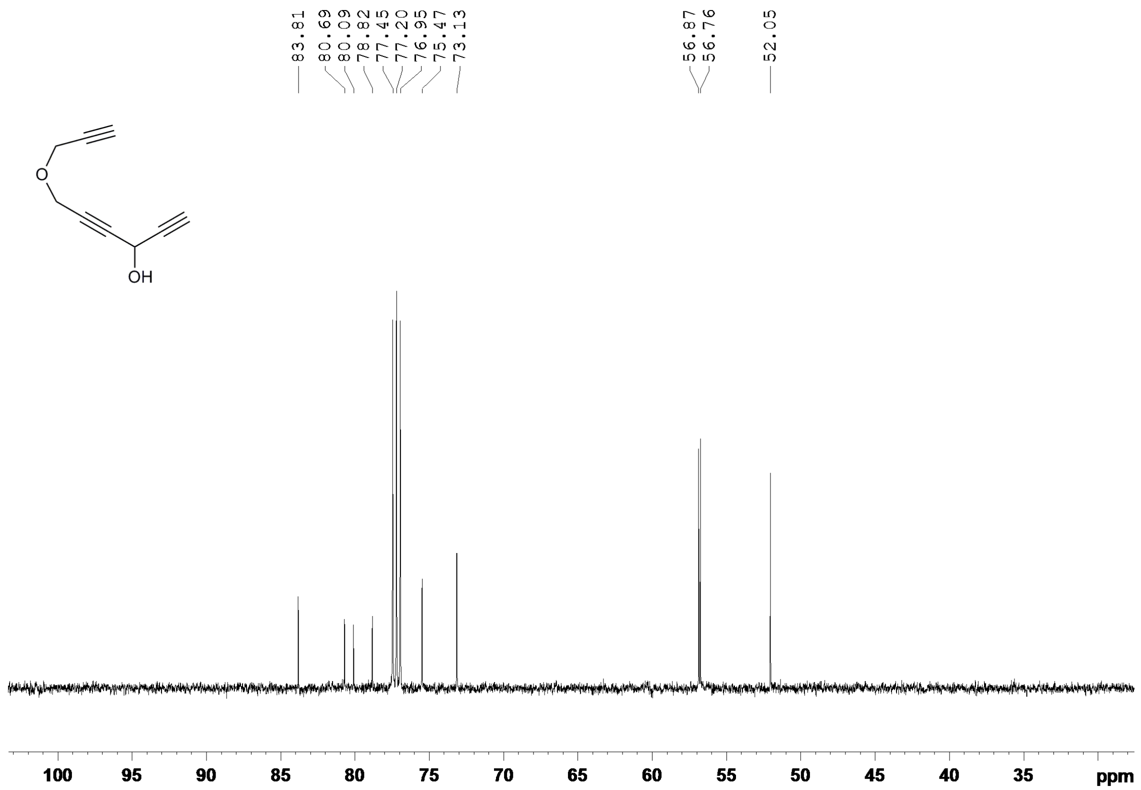

Supplementary Figure 43. ^{13}C NMR spectrum (125.8 MHz, $CDCl_3$) of compound **L3** at room temperature.

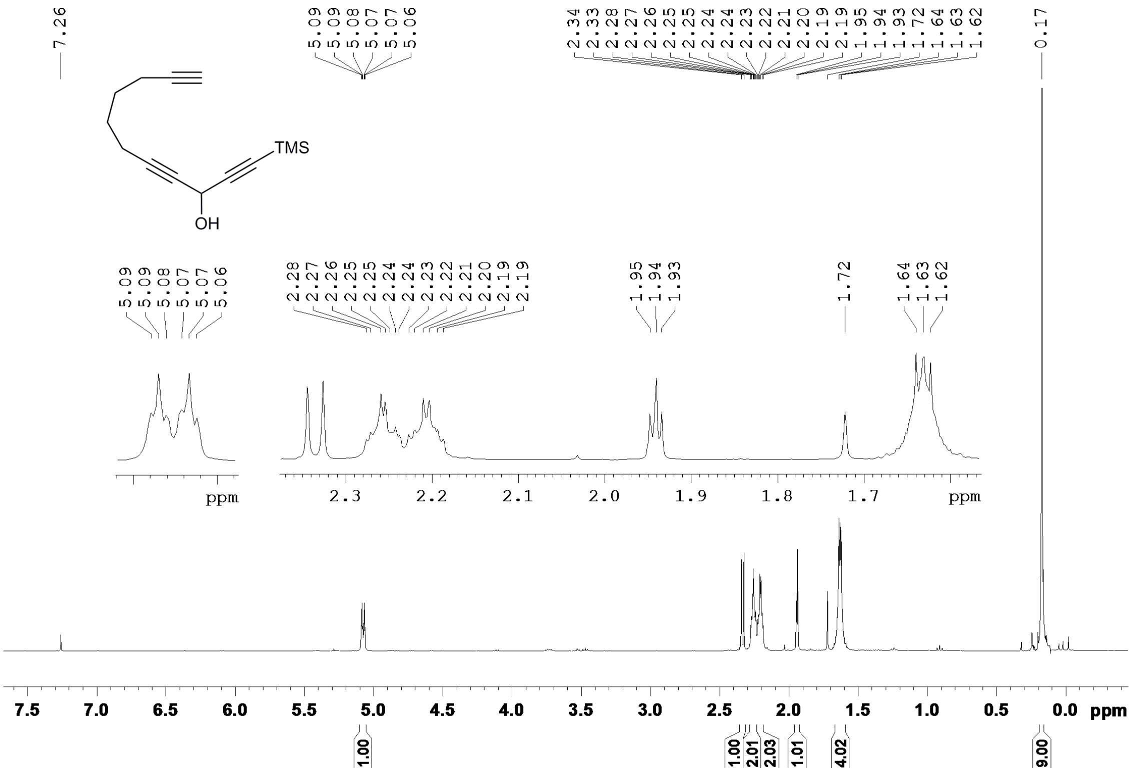

Supplementary Figure 44. ^{1}H NMR spectrum (400.1 MHz, $CDCl_3$) of compound **S4** at room temperature.

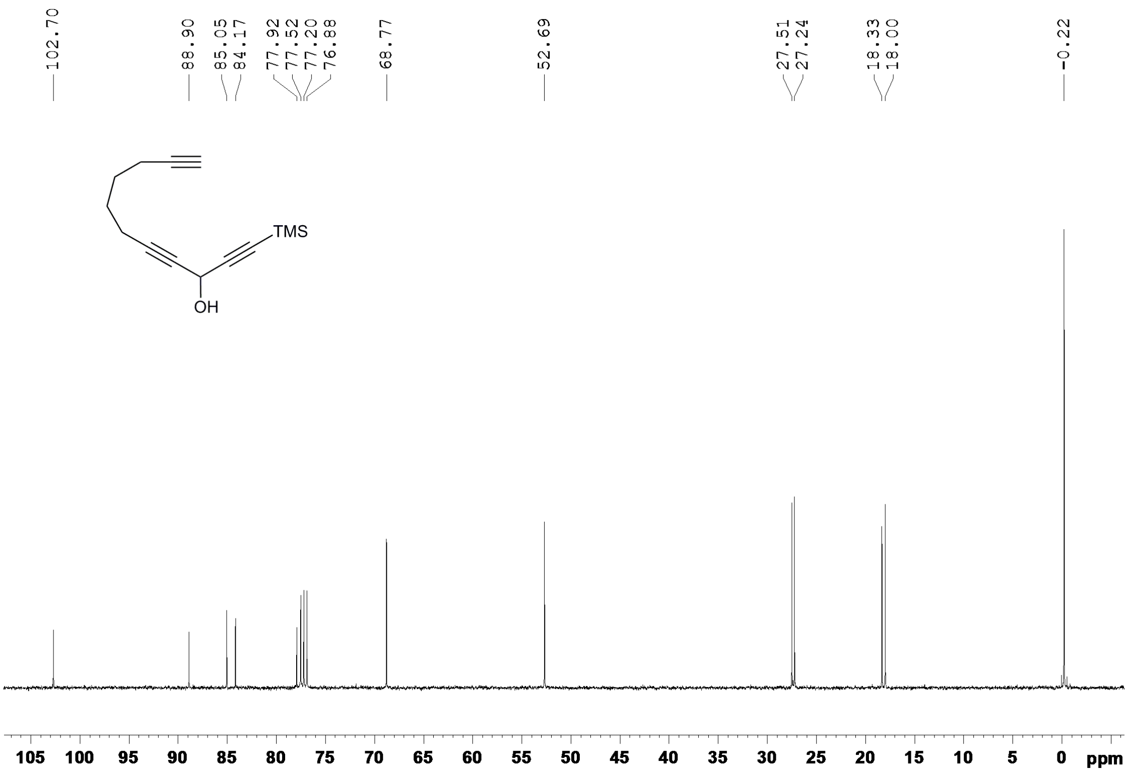

Supplementary Figure 45. ^{13}C NMR spectrum (100.6 MHz, $CDCl_3$) of compound **S4** at room temperature.

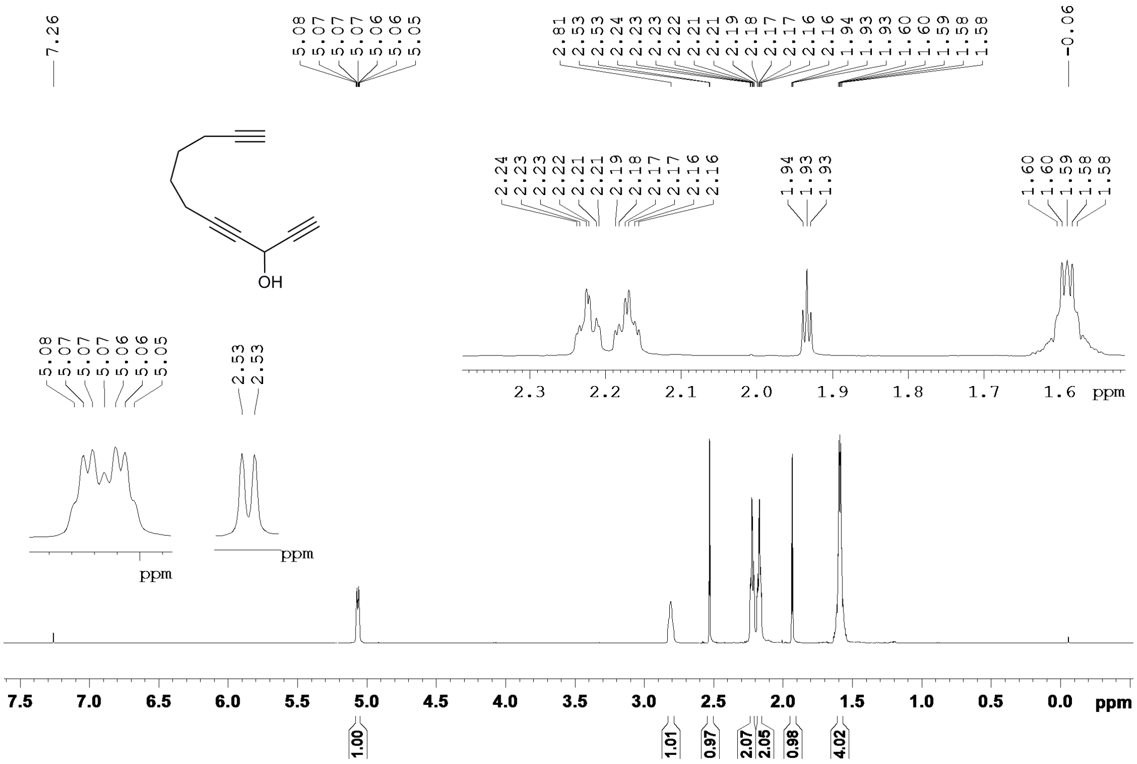

Supplementary Figure 46. ^{1}H NMR spectrum (500.2 MHz, $CDCl_3$) of compound **L4** at room temperature.

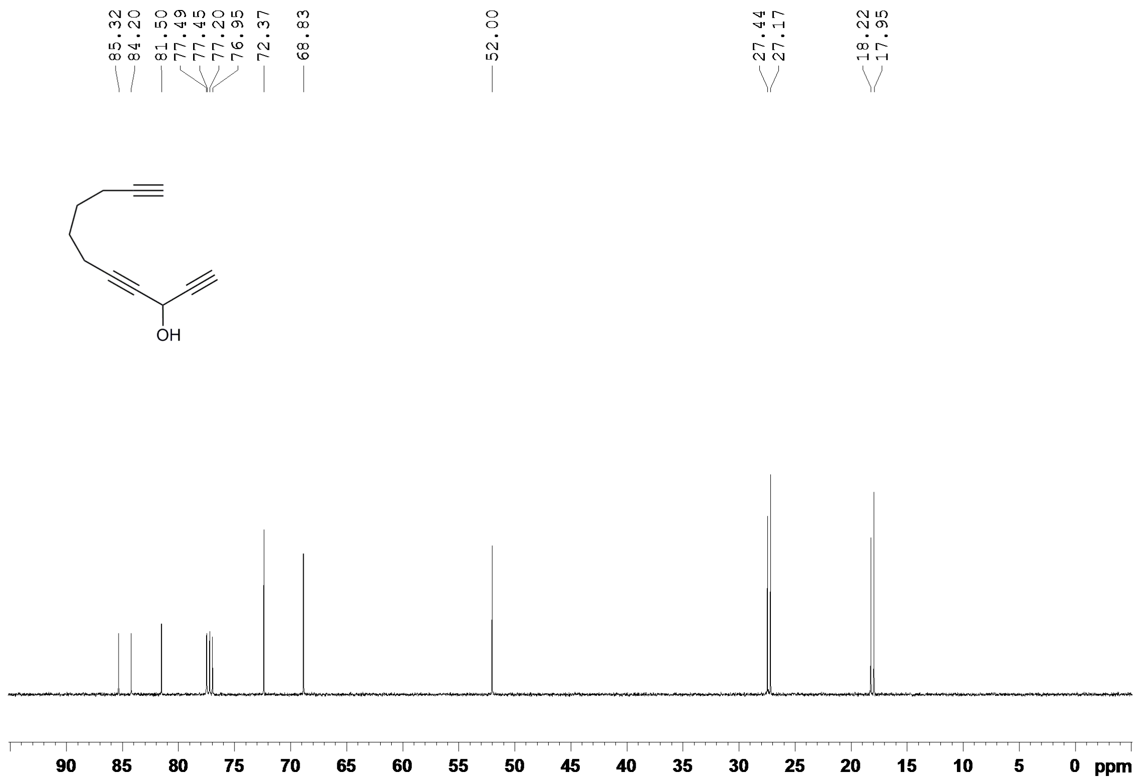

Supplementary Figure 47. ^{13}C NMR spectrum (125.8 MHz, $CDCl_3$) of compound **L4** at room temperature.

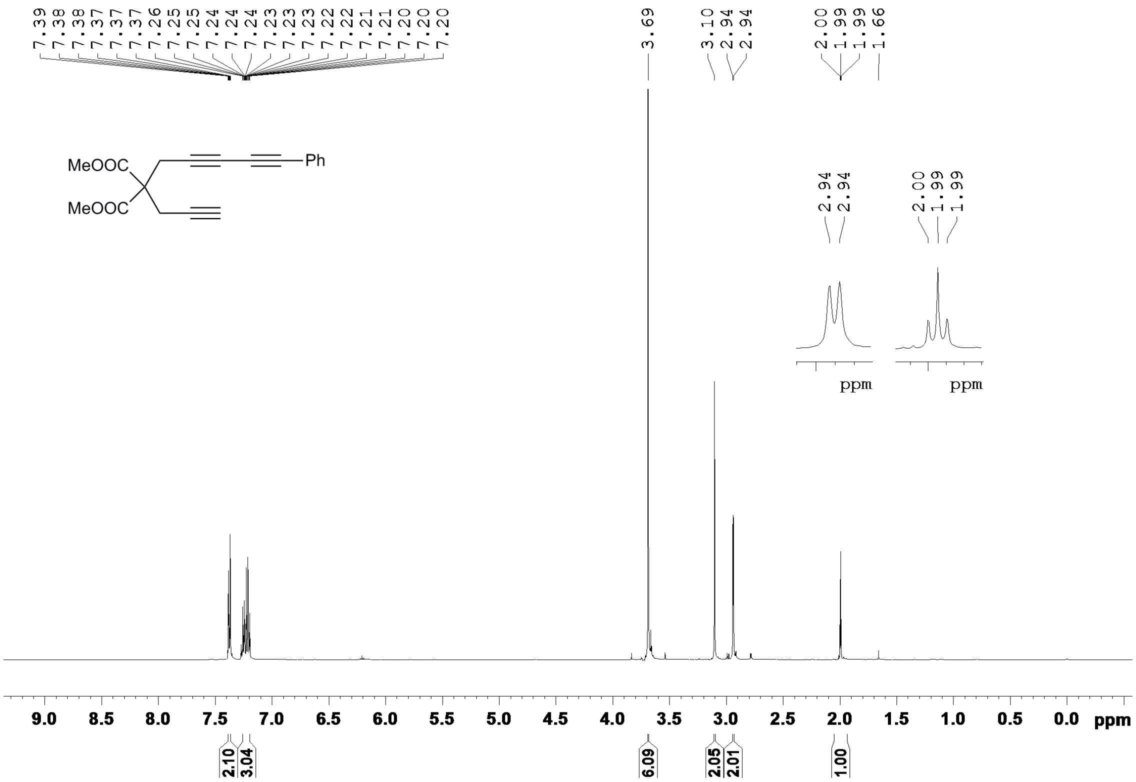

Supplementary Figure 48. ^{1}H NMR spectrum (500.2 MHz, $CDCl_3$) of compound **S5** at room temperature.

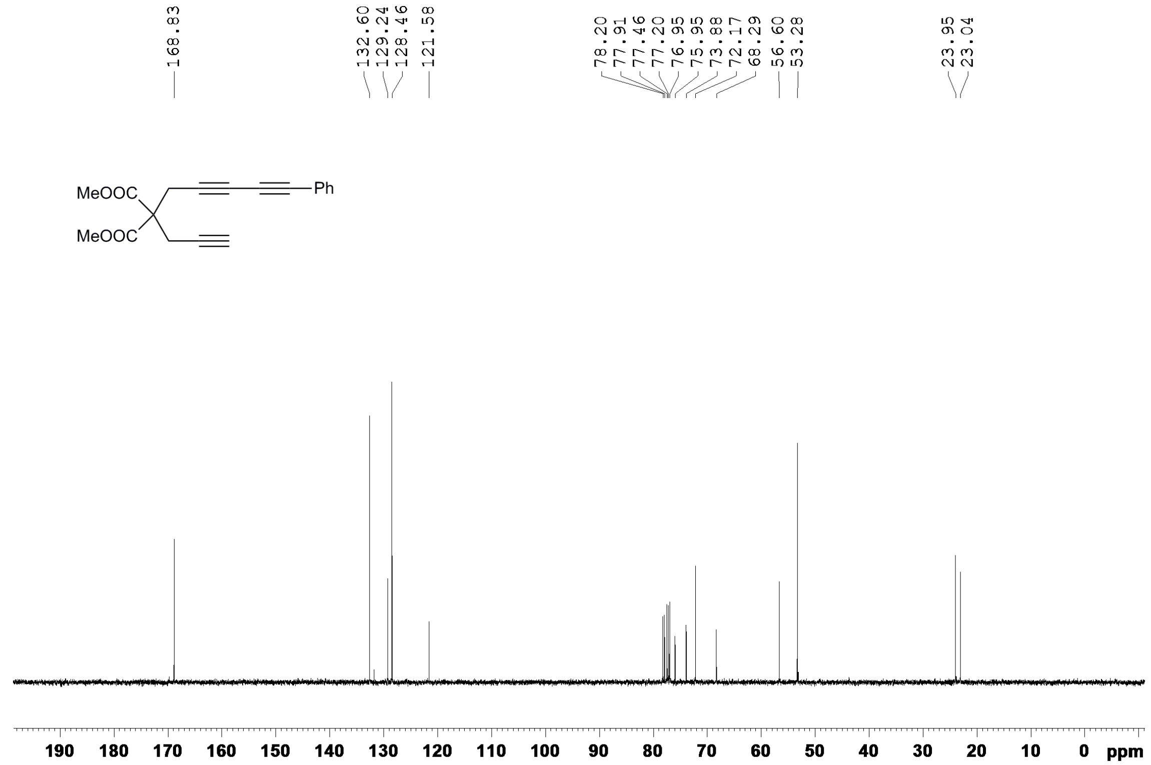

Supplementary Figure 49. ^{13}C NMR spectrum (125.8 MHz, $CDCl_3$) of compound **S5** at room temperature.

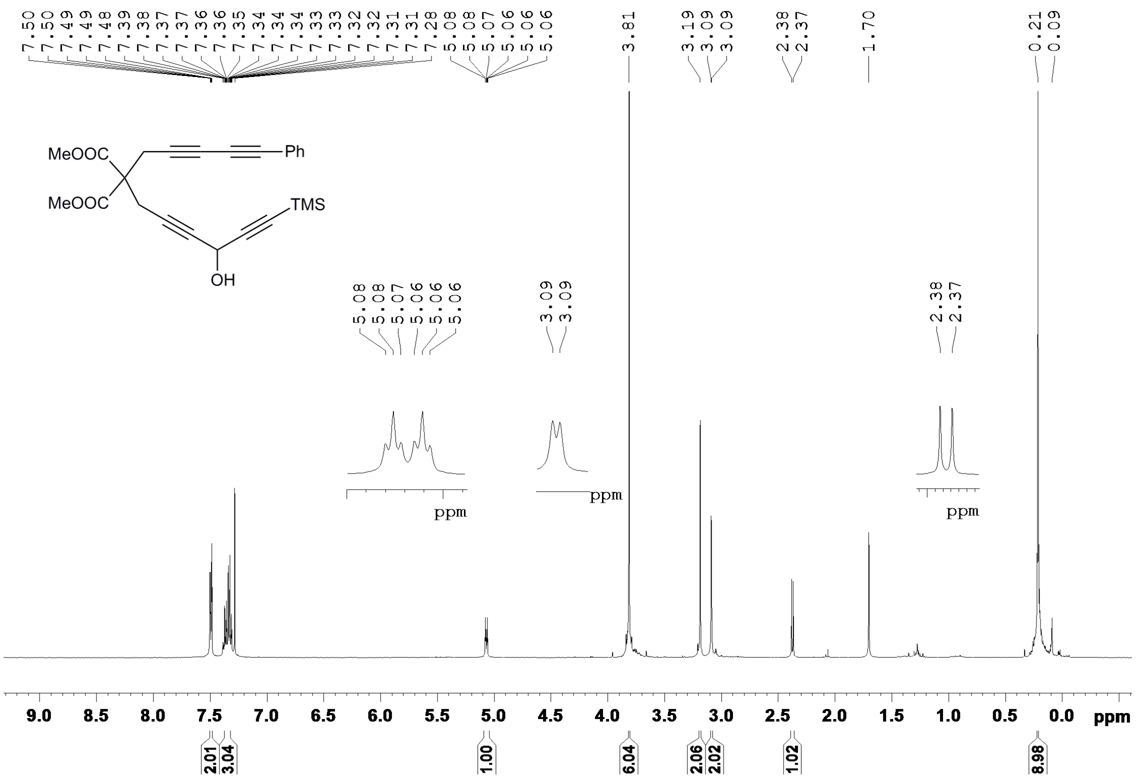

Supplementary Figure 50. ^{1}H NMR spectrum (500.2 MHz, $CDCl_3$) of compound **S6** at room temperature.

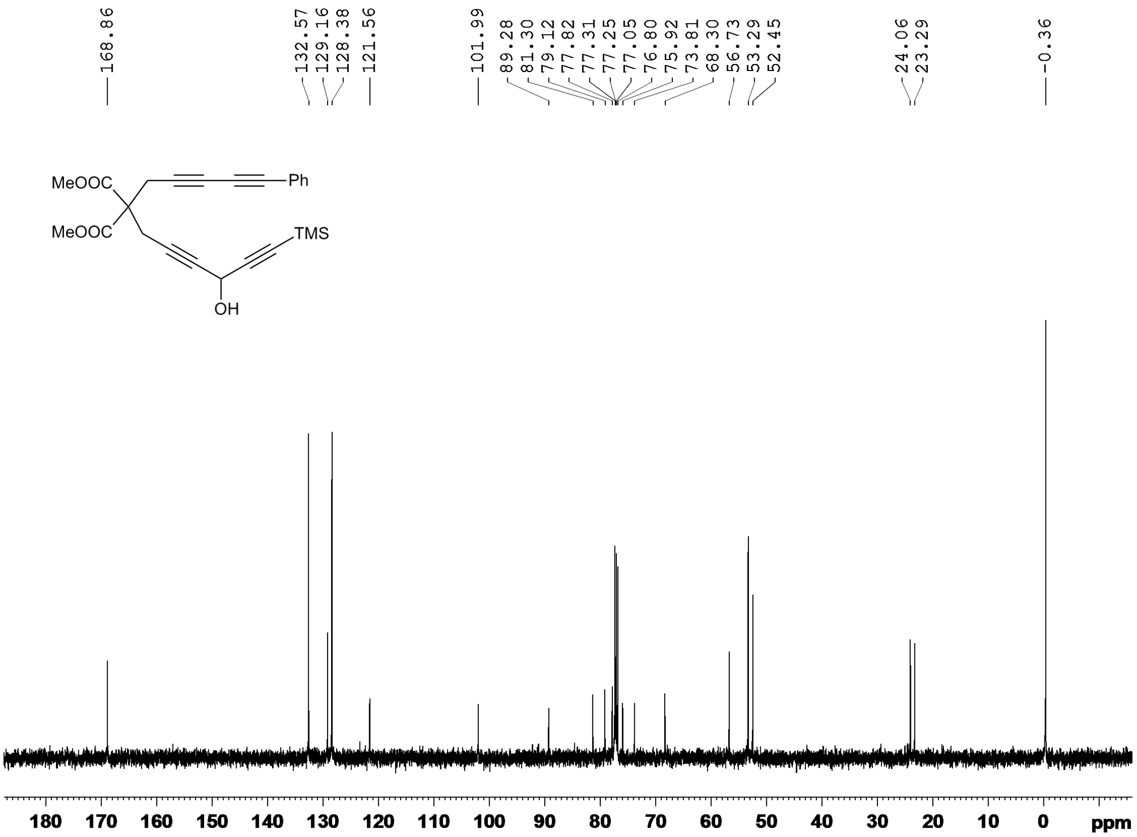

Supplementary Figure 51. ^{13}C NMR spectrum (125.8 MHz, $CDCl_3$) of compound **S6** at room temperature.

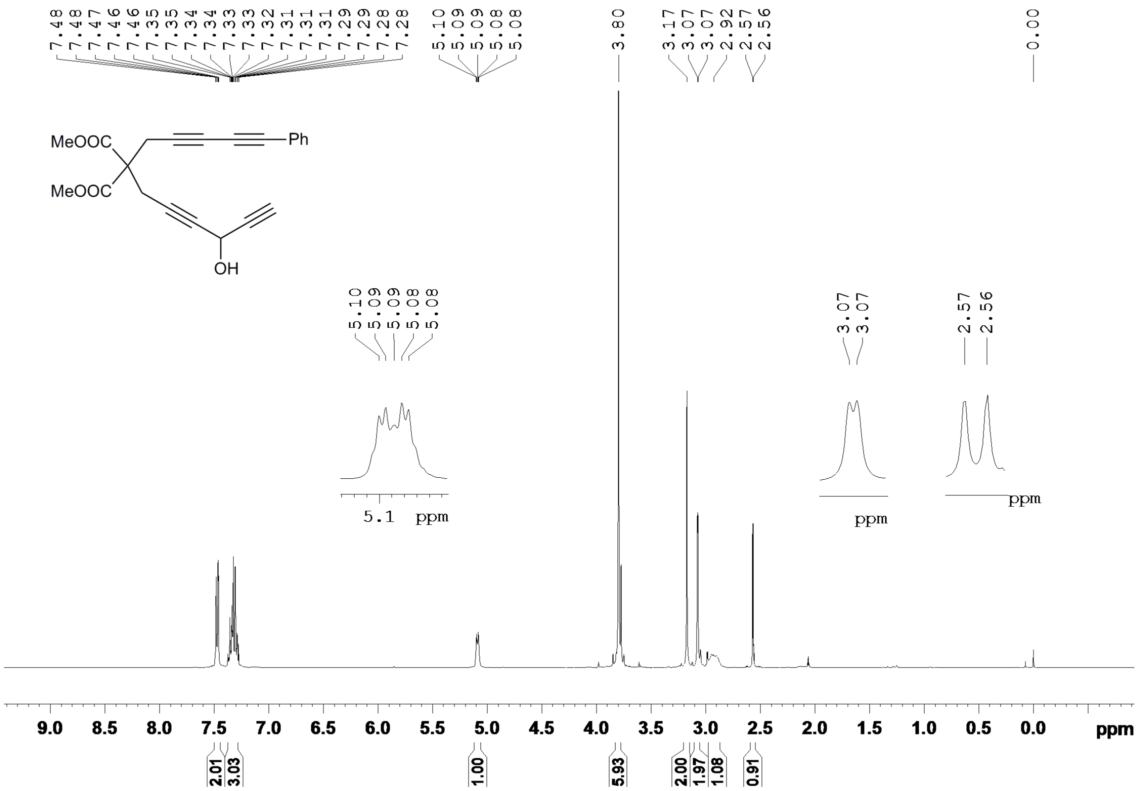

Supplementary Figure 52. ^{1}H NMR spectrum (400.1 MHz, $CDCl_3$) of compound **L5** at room temperature.

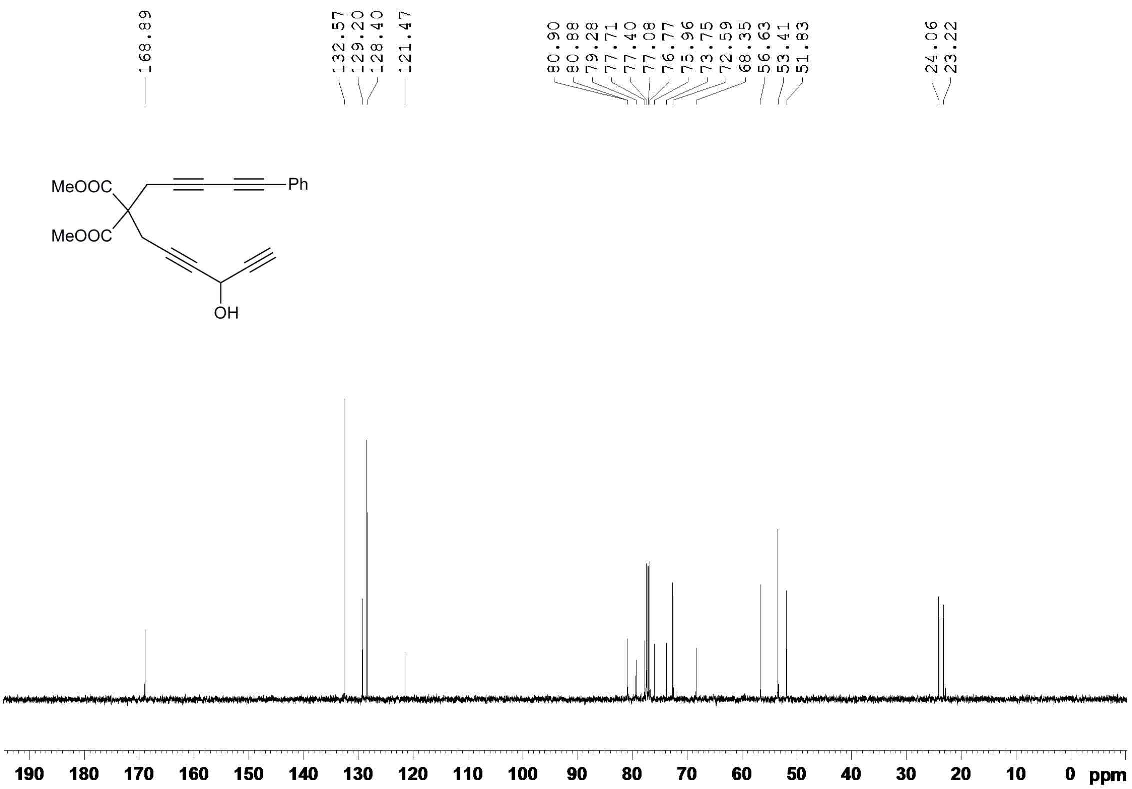

Supplementary Figure 53. ^{13}C NMR spectrum (100.6 MHz, $CDCl_3$) of compound **L5** at room temperature.

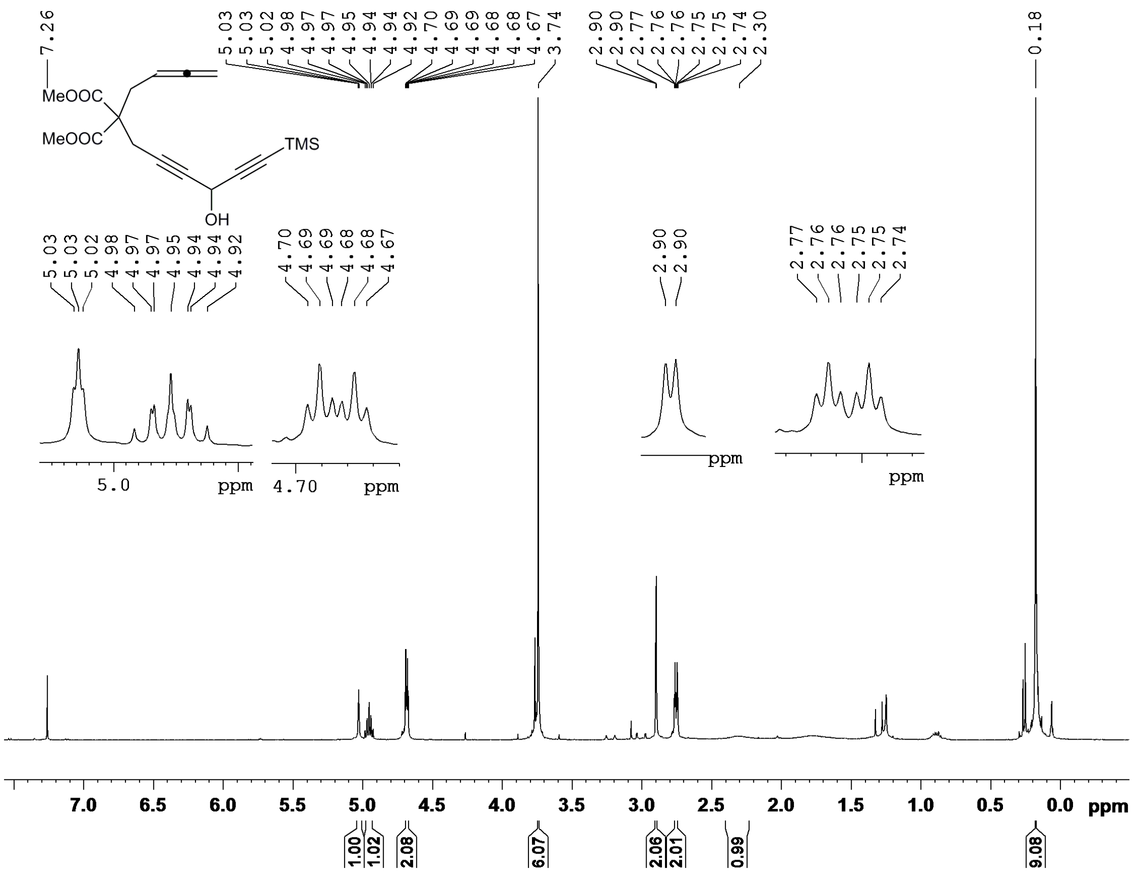

Supplementary Figure 54. ^{1}H NMR spectrum (500.2 MHz, $CDCl_3$) of compound **S7** at room temperature.

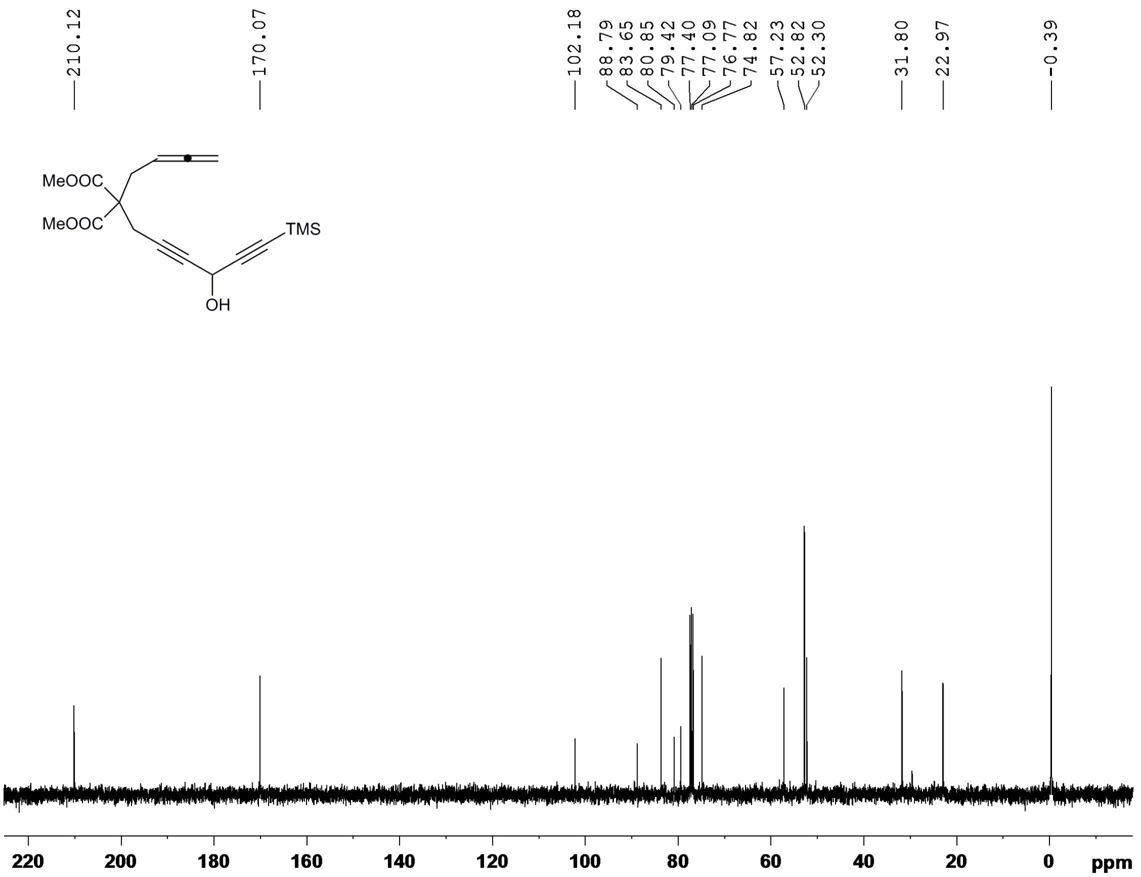

Supplementary Figure 55. ^{13}C NMR spectrum (100.6 MHz, $CDCl_3$) of compound **S7** at room temperature.

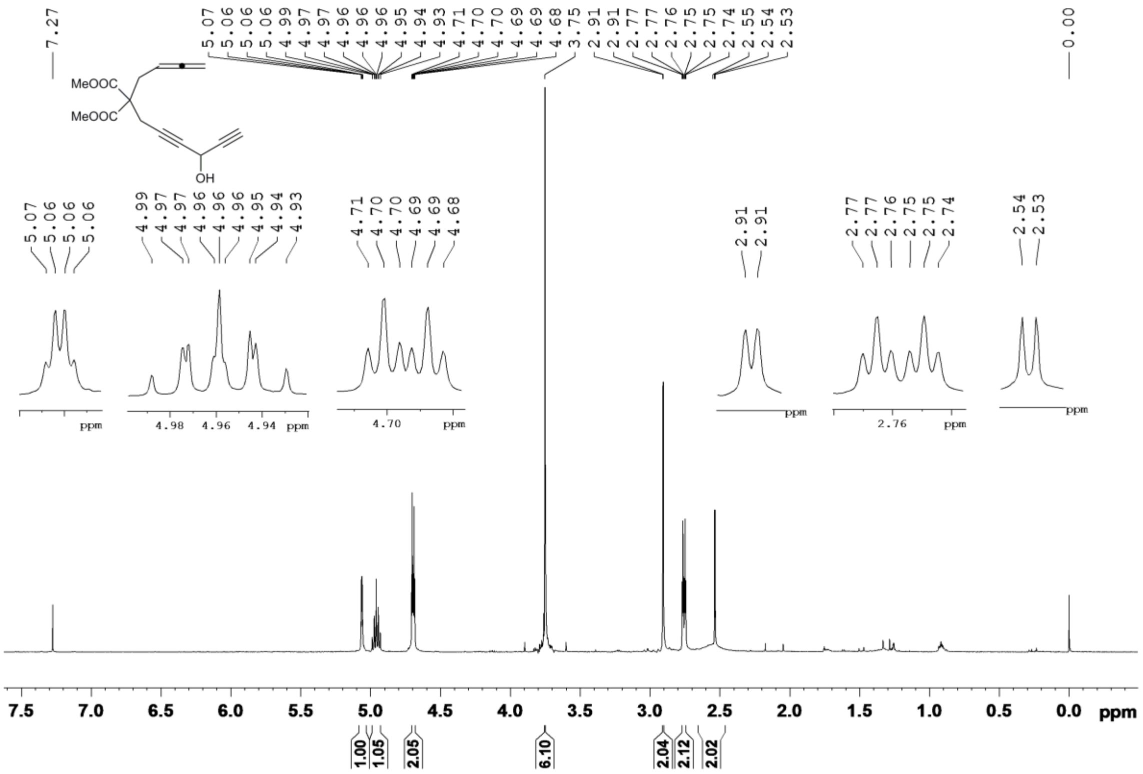

Supplementary Figure 56. ^{1}H NMR spectrum (500.2 MHz, CD_3Cl) of compound **L6** at room temperature.

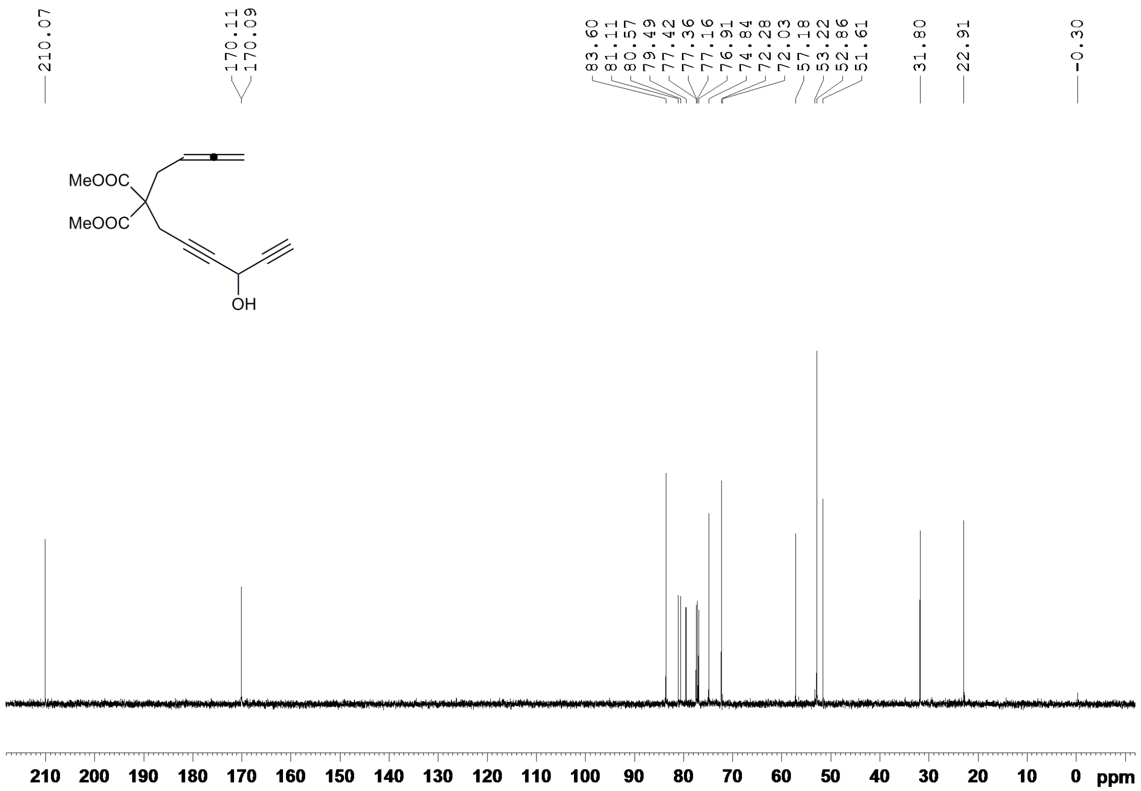

Supplementary Figure 57. ^{13}C NMR spectrum (125.8 MHz, $CDCl_3$) of compound **L6** at room temperature.

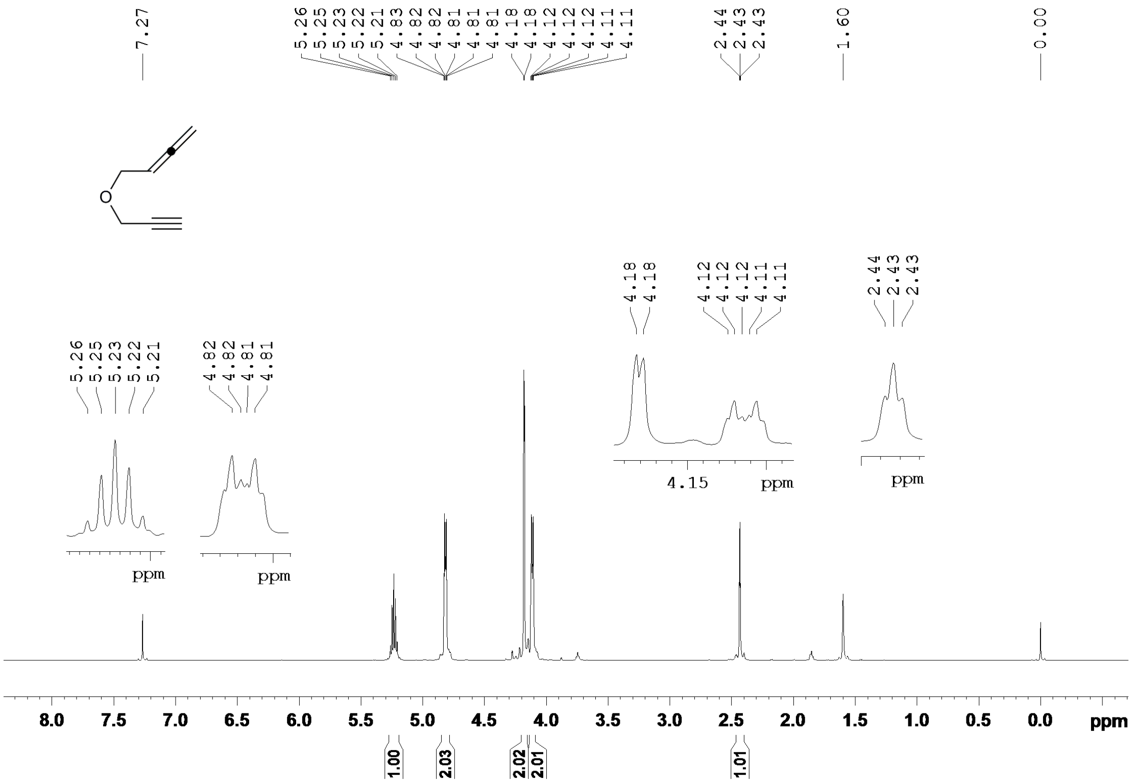

Supplementary Figure 58. ^{1}H NMR spectrum (500.2 MHz, CD_3Cl) of compound **S8** at room temperature.

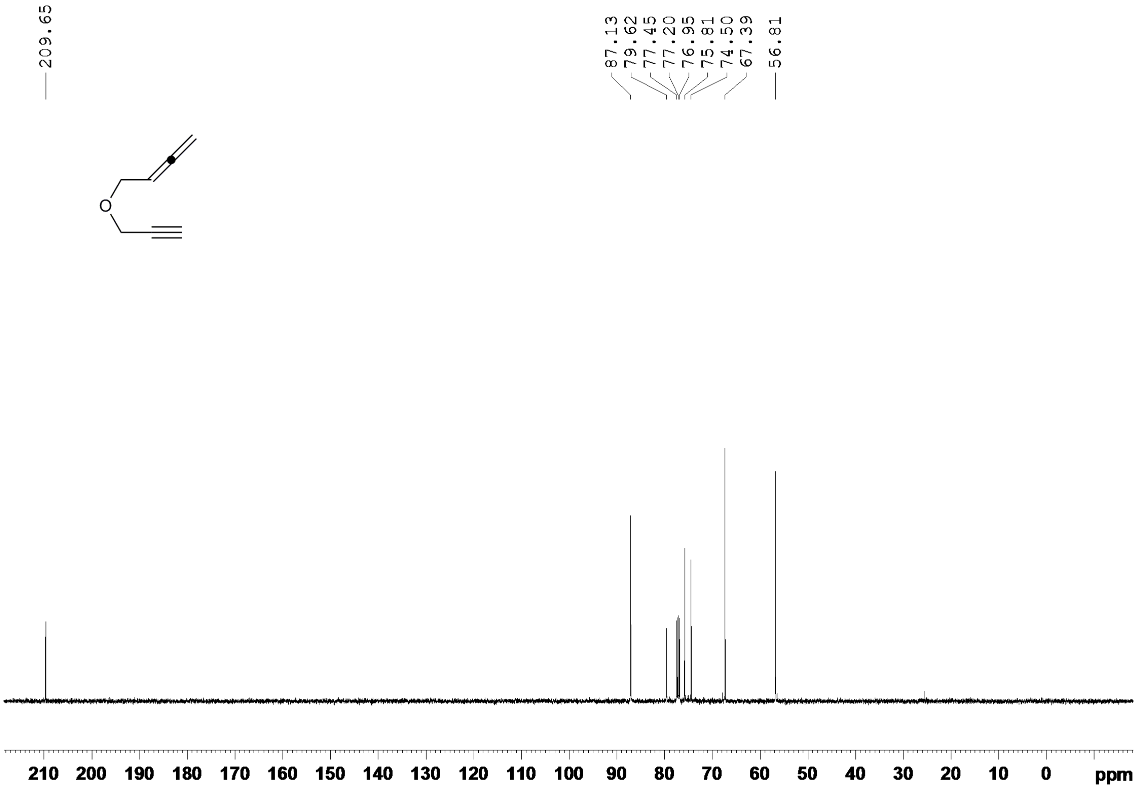

Supplementary Figure 59. ^{13}C NMR spectrum (125.8 MHz, $CDCl_3$) of compound **S8** at room temperature.

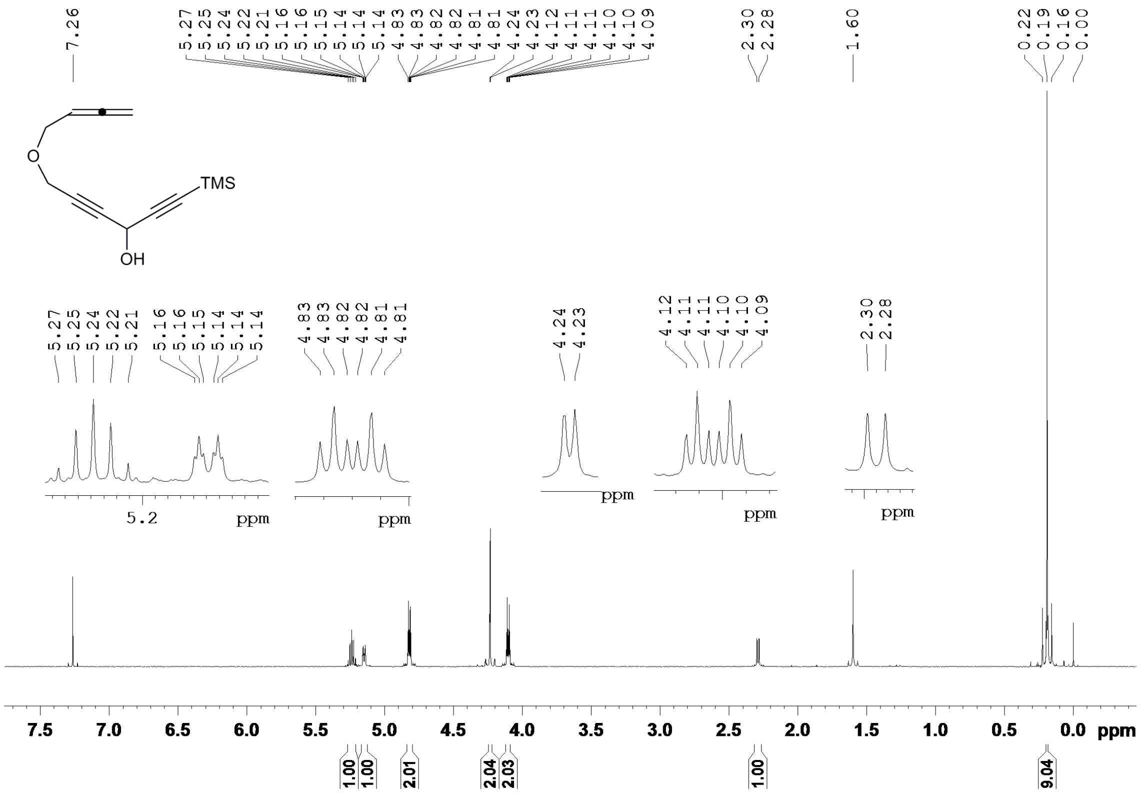

Supplementary Figure 60. ^{1}H NMR spectrum (500.2 MHz, CD_3Cl) of compound **S9** at room temperature.

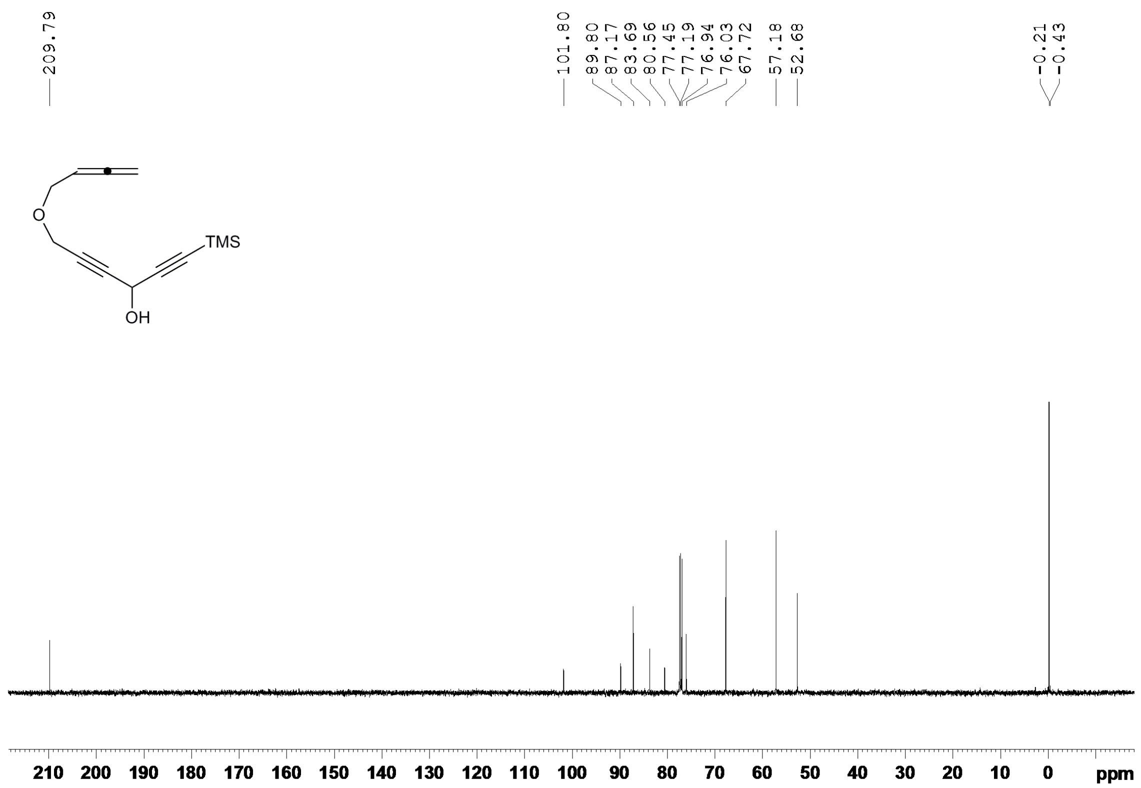

Supplementary Figure 61. ^{13}C NMR spectrum (125.8 MHz, $CDCl_3$) of compound **S9** at room temperature.

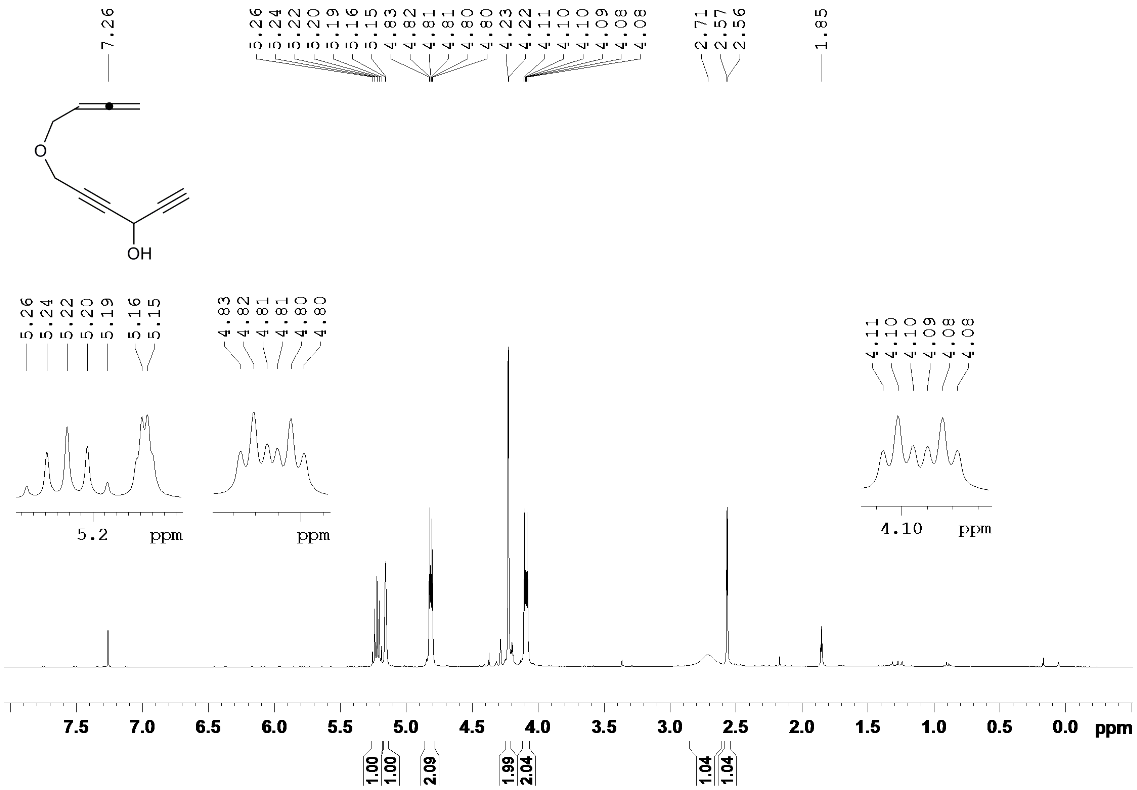

Supplementary Figure 62. ^{1}H NMR spectrum (400.1 MHz, $CDCl_3$) of compound **L7** at room temperature.

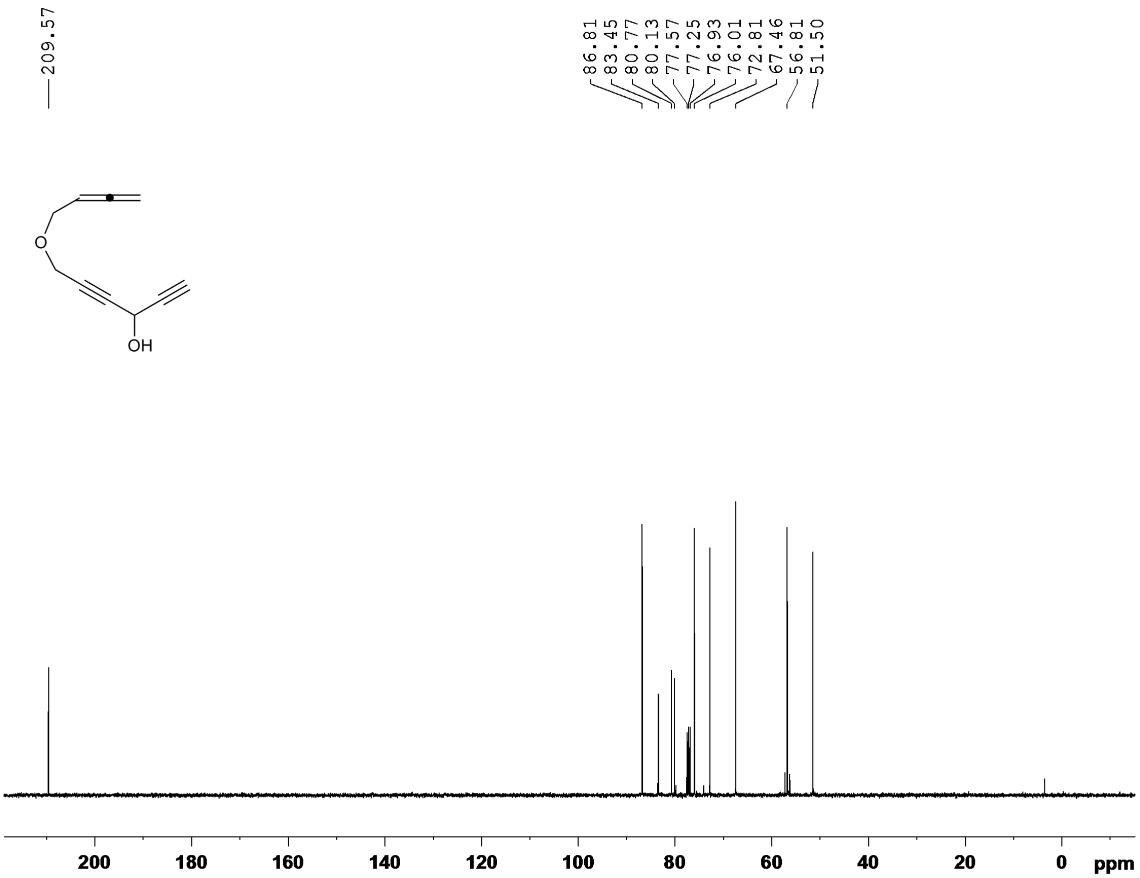

Supplementary Figure 63. ^{13}C NMR spectrum (100.6 MHz, $CDCl_3$) of compound **L7** at room temperature.

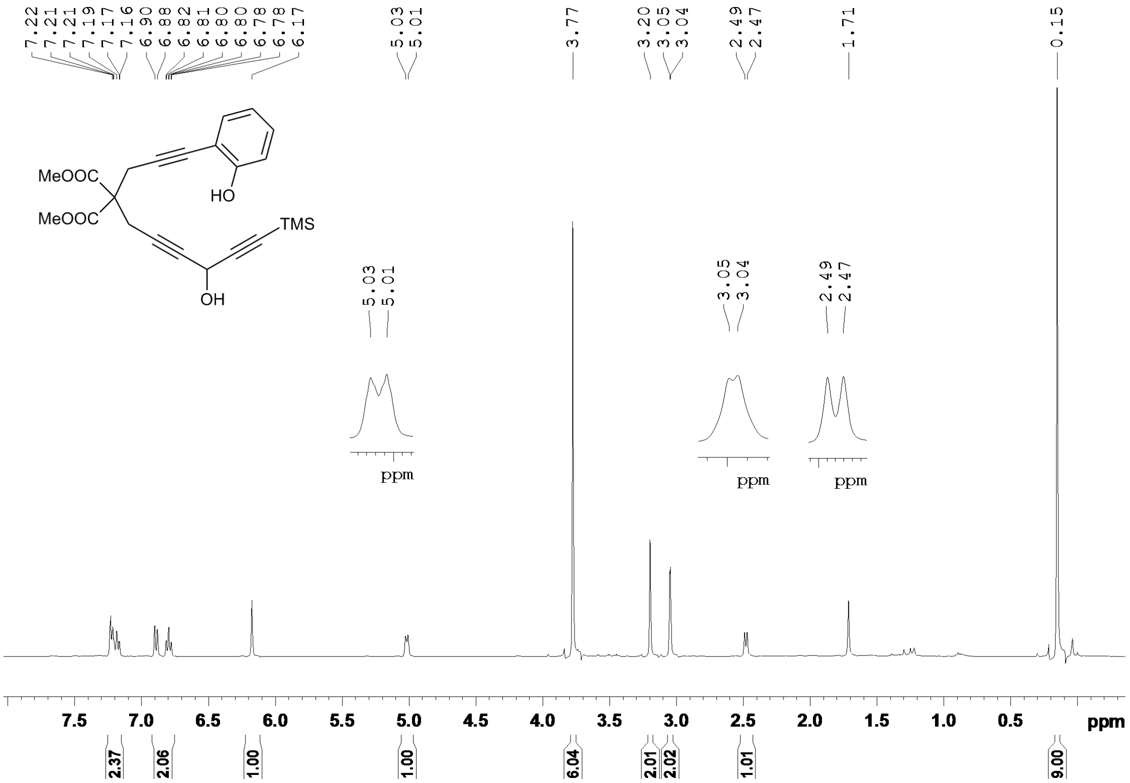

Supplementary Figure 64. ^{1}H NMR spectrum (400.1 MHz, $CDCl_3$) of compound **S10** at room temperature.

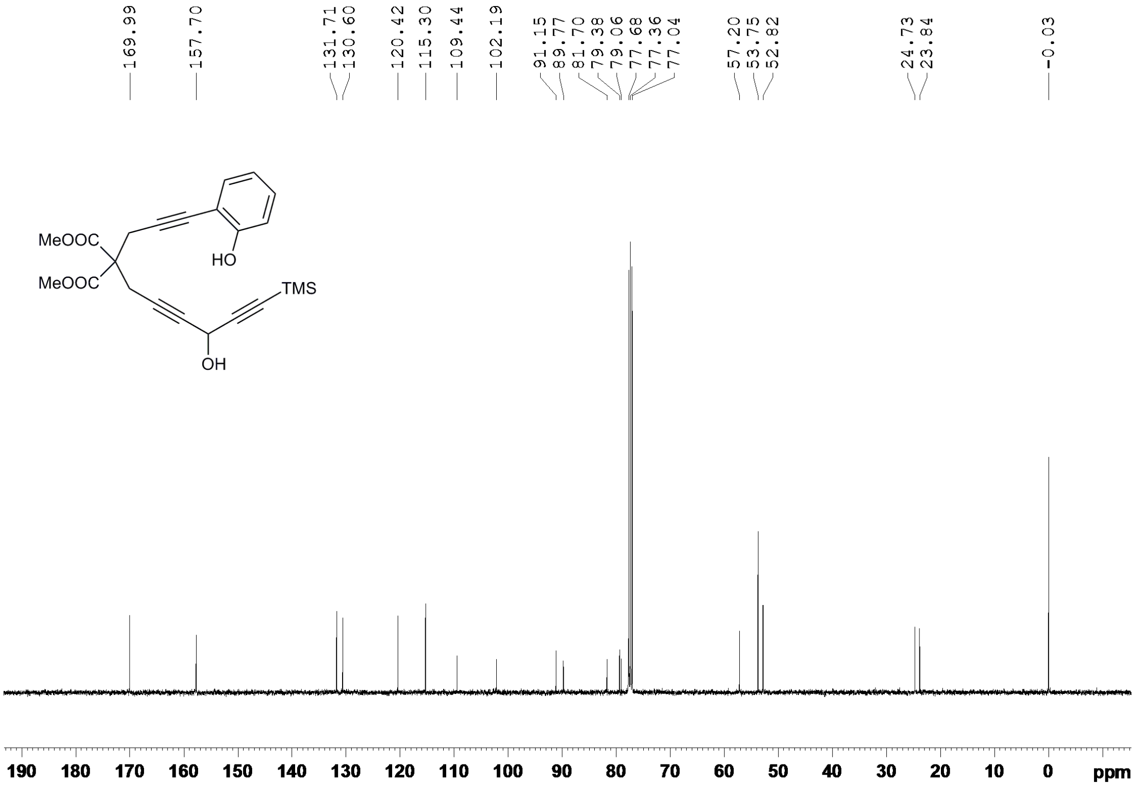

Supplementary Figure 65. ^{13}C NMR spectrum (100.6 MHz, $CDCl_3$) of compound **S10** at room temperature.

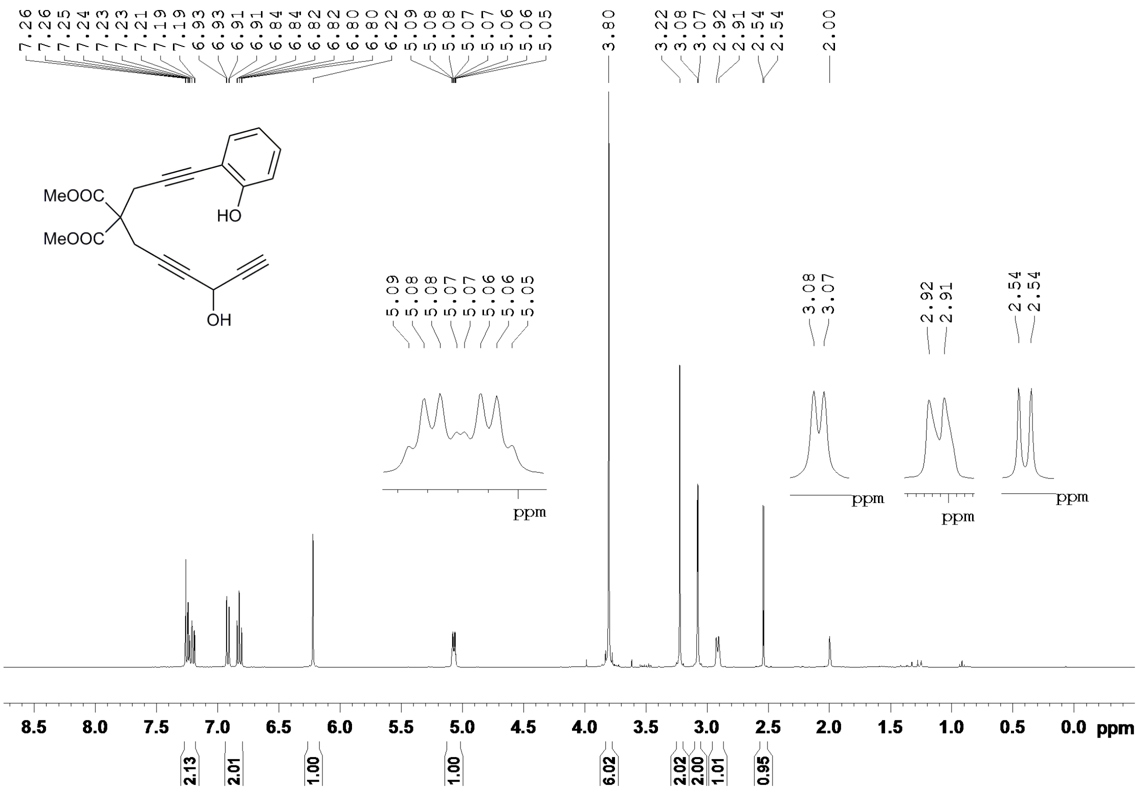

Supplementary Figure 66. ^{1}H NMR spectrum (400.1 MHz, $CDCl_3$) of compound **L8** at room temperature.

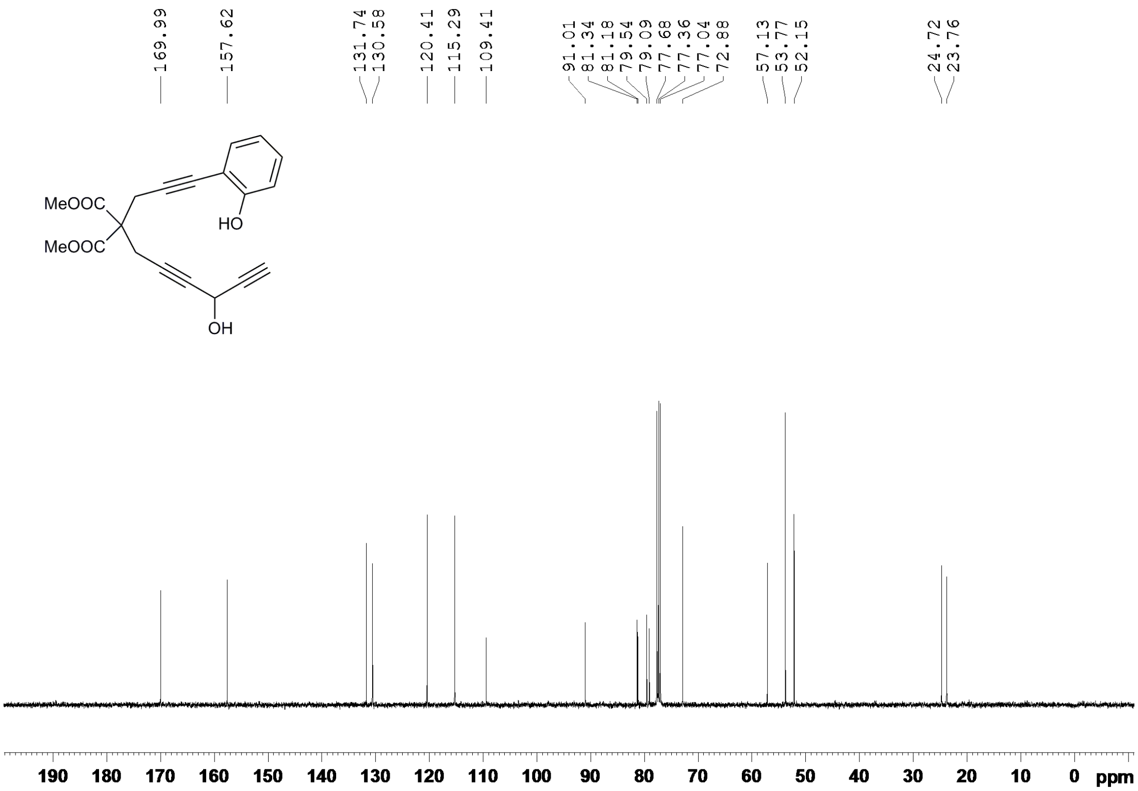

Supplementary Figure 67. ^{13}C NMR spectrum (100.6 MHz, $CDCl_3$) of compound **L8** at room temperature.

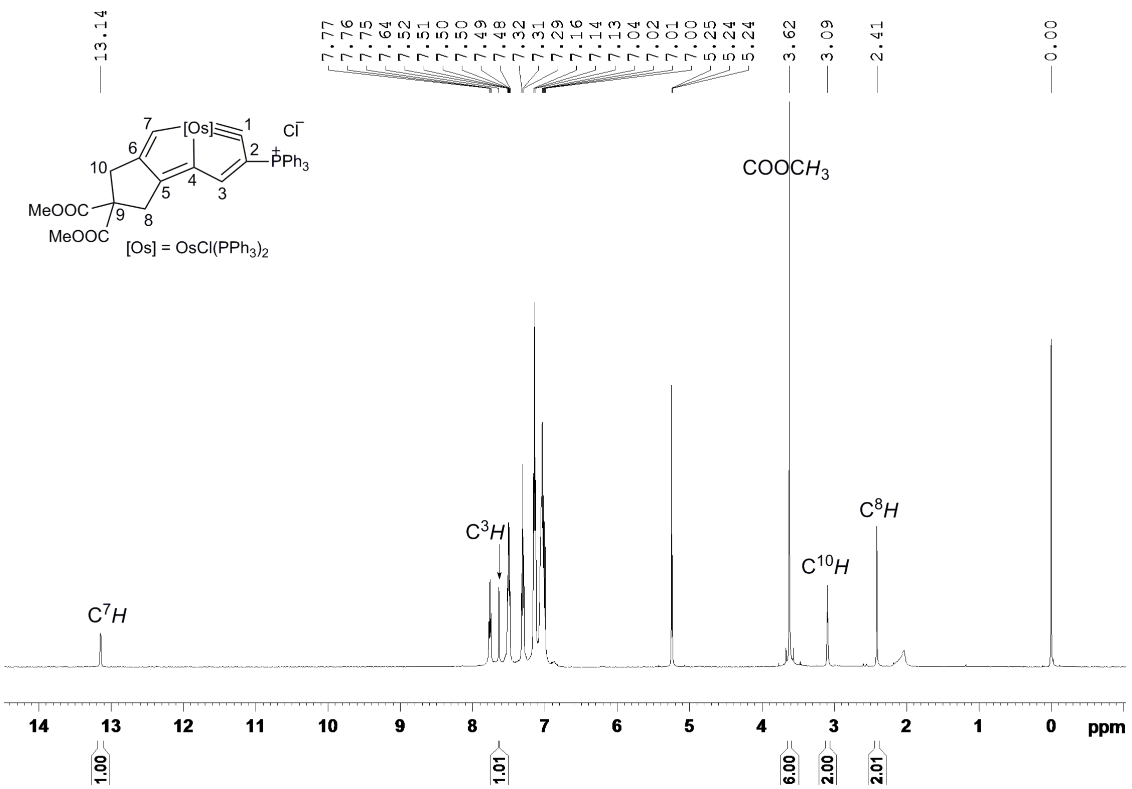

Supplementary Figure 68. ^{1}H NMR spectrum (500.2 MHz, CD_2Cl_2) of complex **1** at room temperature.

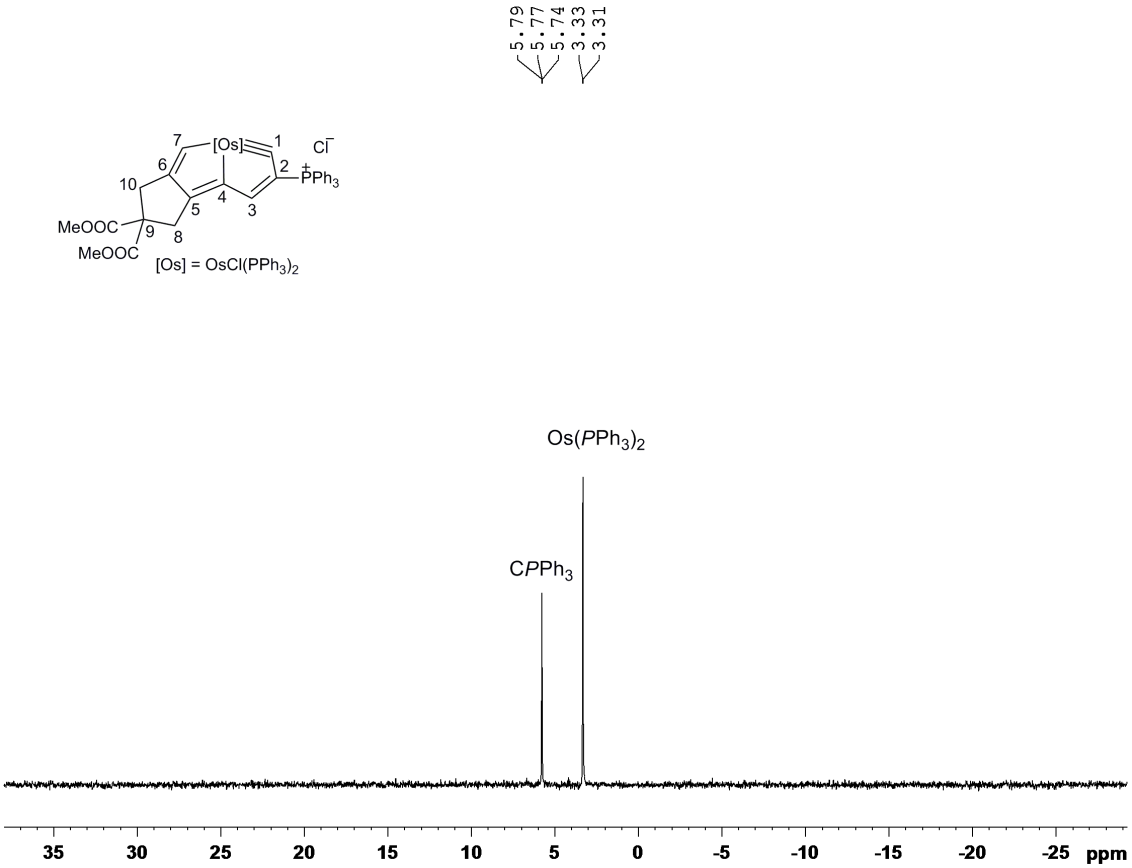

Supplementary Figure 69. ^{31}P NMR spectrum (202.5 MHz, CD_2Cl_2) of complex **1** at room temperature.

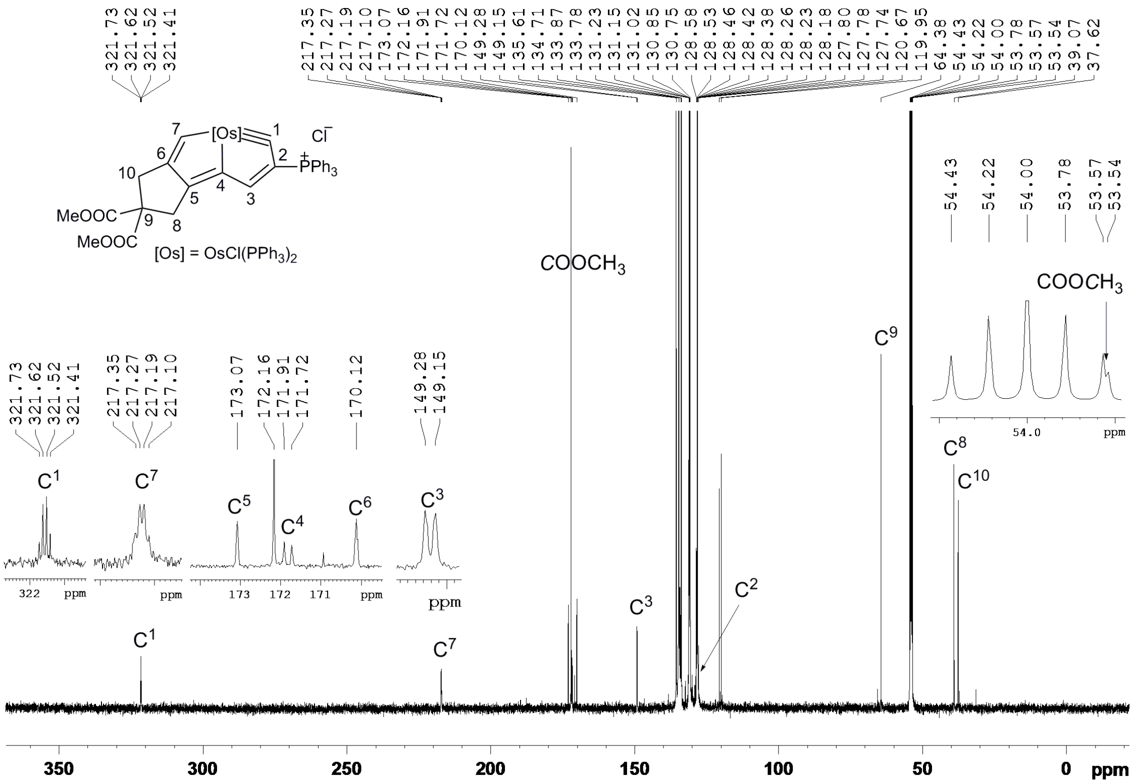

Supplementary Figure 70. ^{13}C NMR spectrum (125.8 MHz, CD_2Cl_2) of complex **1** at room temperature.

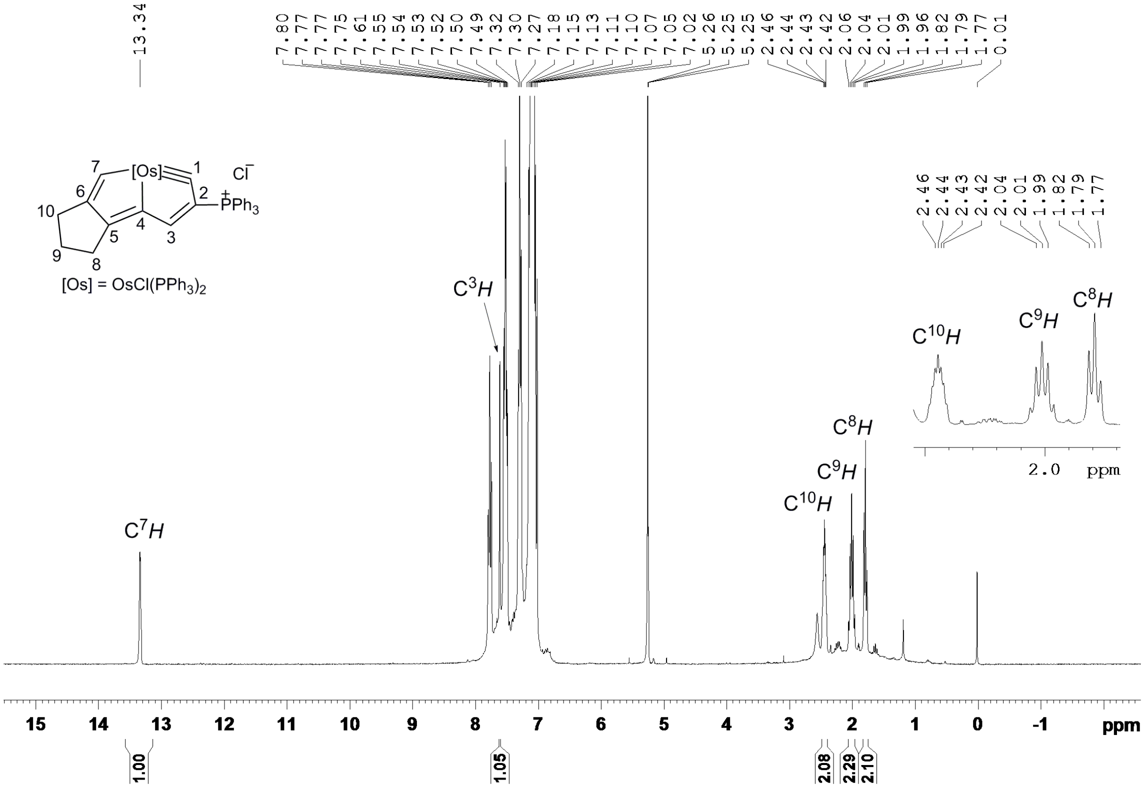

Supplementary Figure 71. ^{1}H NMR spectrum (300.1 MHz, CD_2Cl_2) of complex **2** at room temperature.

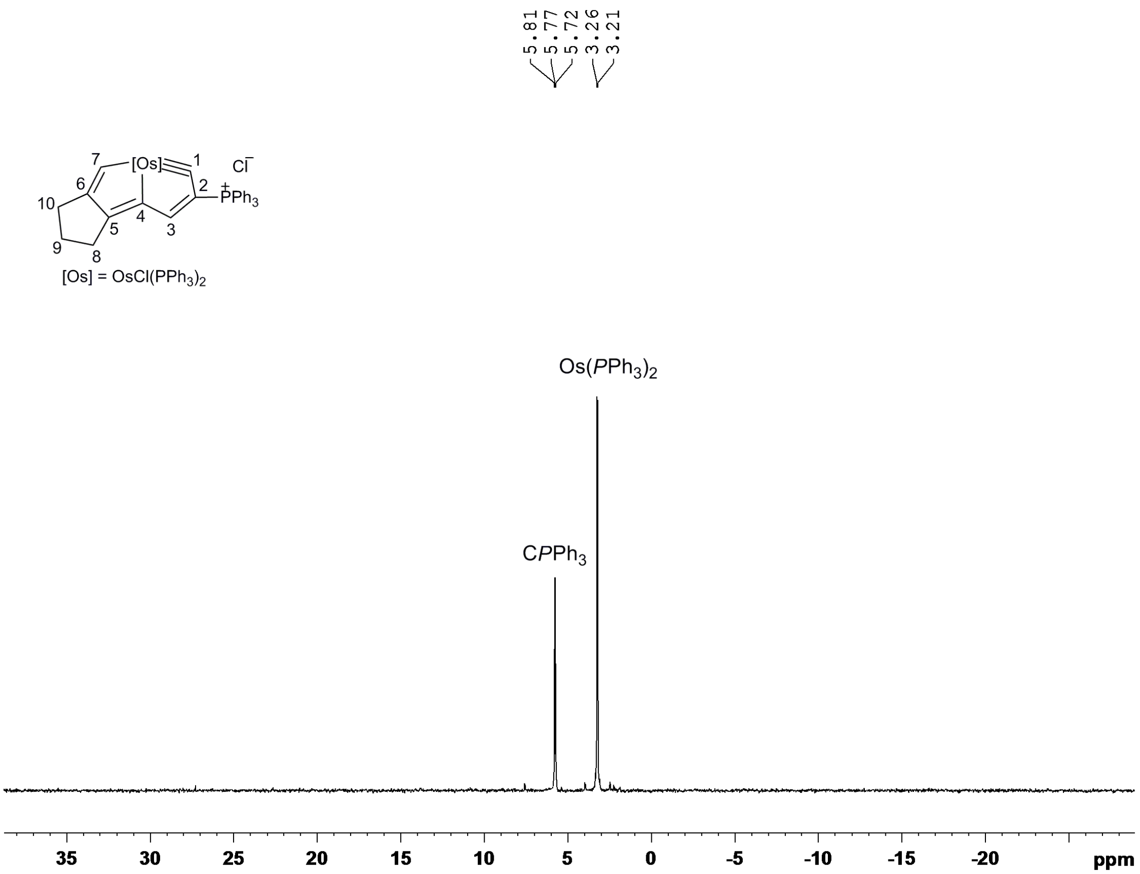

Supplementary Figure 72. ^{31}P NMR spectrum (121.5 MHz, CD_2Cl_2) of complex **2** at room temperature.

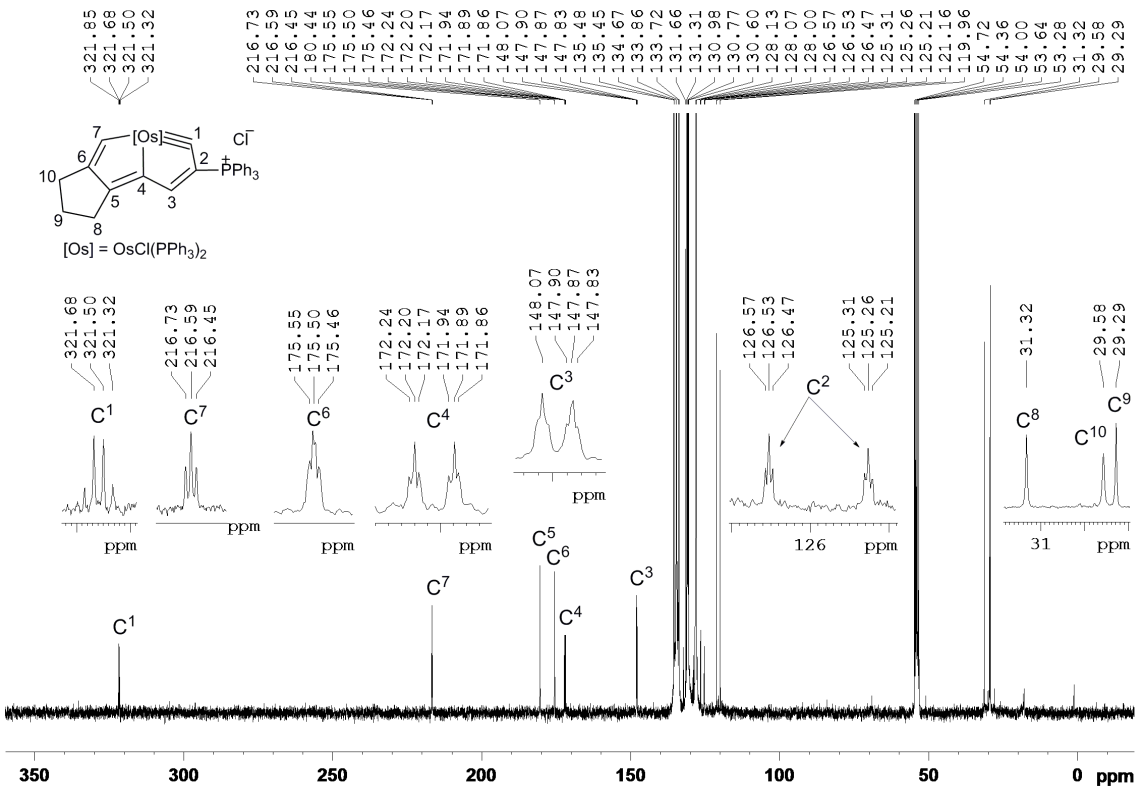

Supplementary Figure 73. ^{13}C NMR spectrum (75.5 MHz, CD_2Cl_2) of complex **2** at room temperature**.**

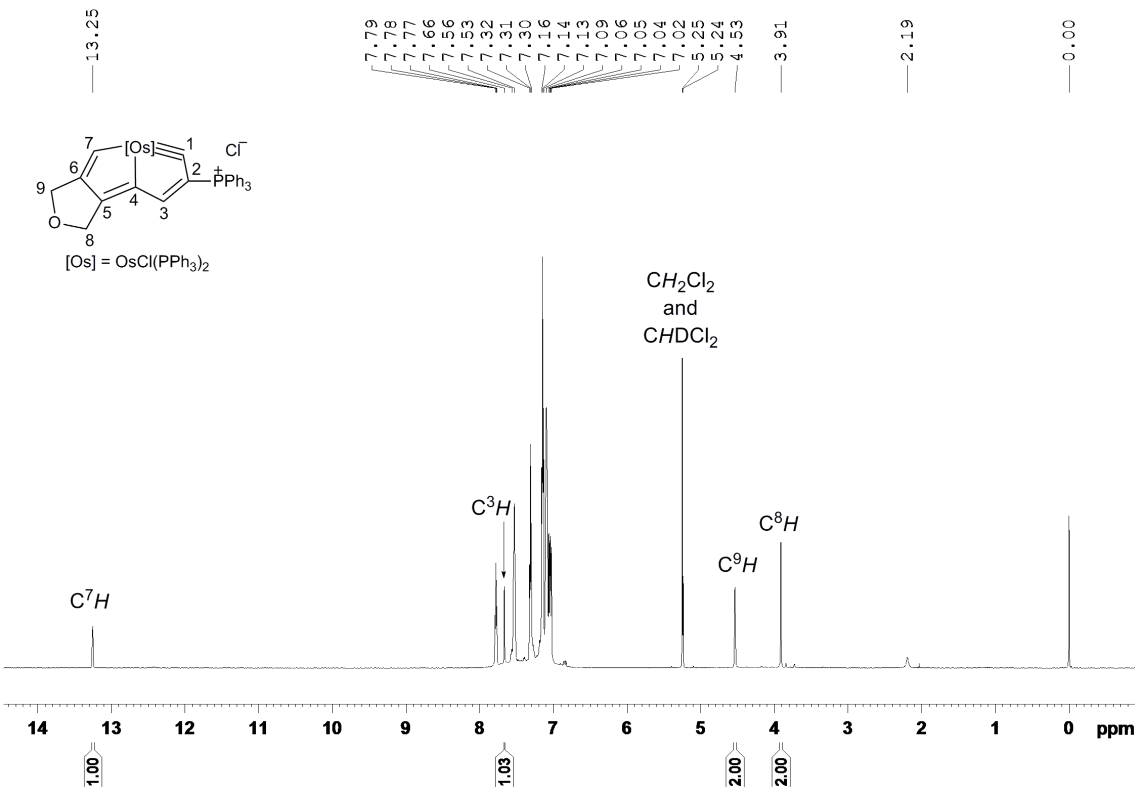

Supplementary Figure 74. ^{1}H NMR spectrum (600.1 MHz, CD_2Cl_2) of complex **3** at room temperature.

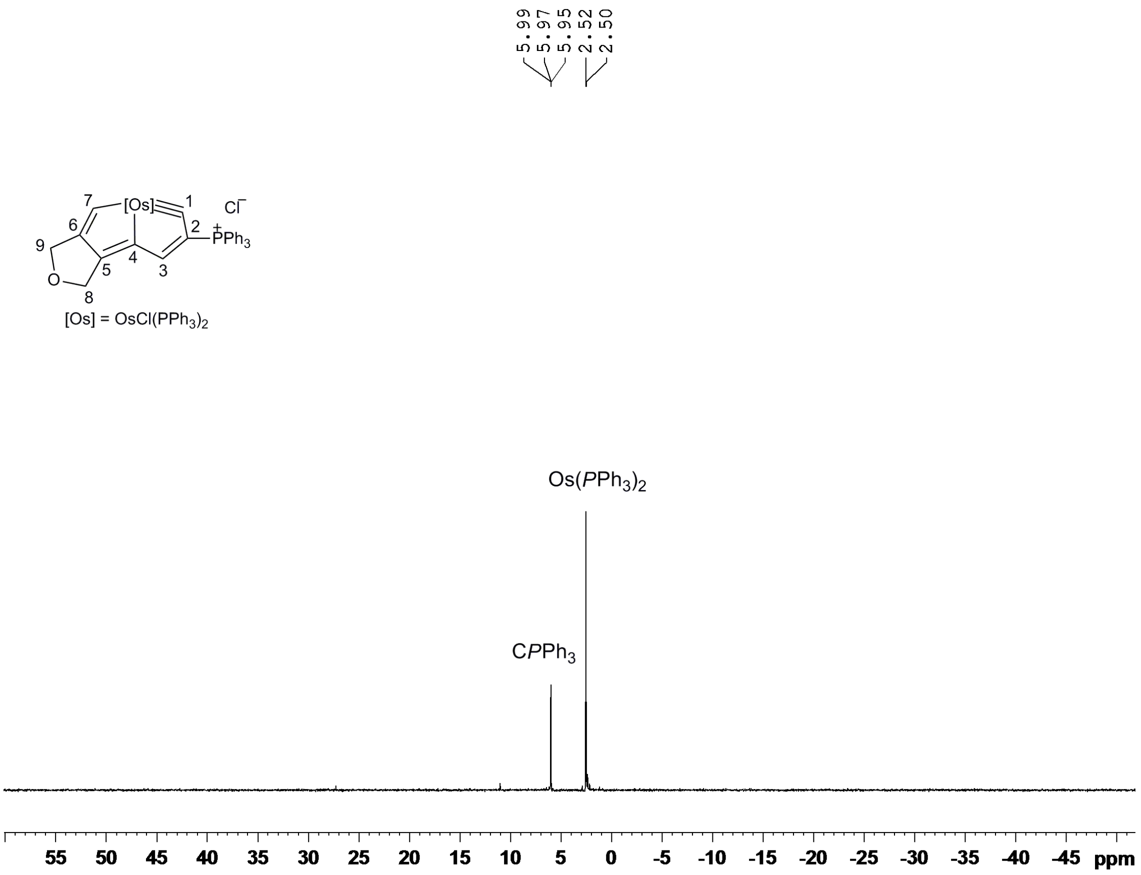

Supplementary Figure 75. ^{31}P NMR spectrum (242.9 MHz, CD_2Cl_2) of complex **3** at room temperature.

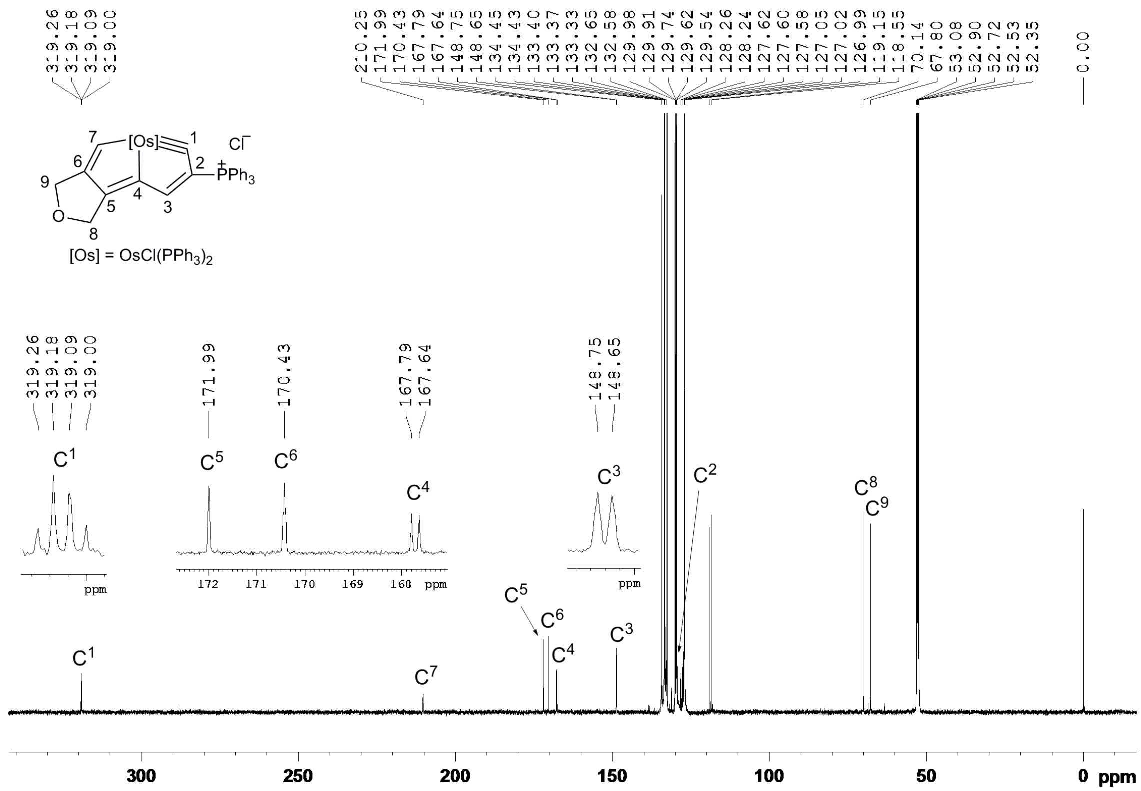

Supplementary Figure 76. ^{13}C NMR spectrum (150.9 MHz, CD_2Cl_2) of complex **3** at room temperature.

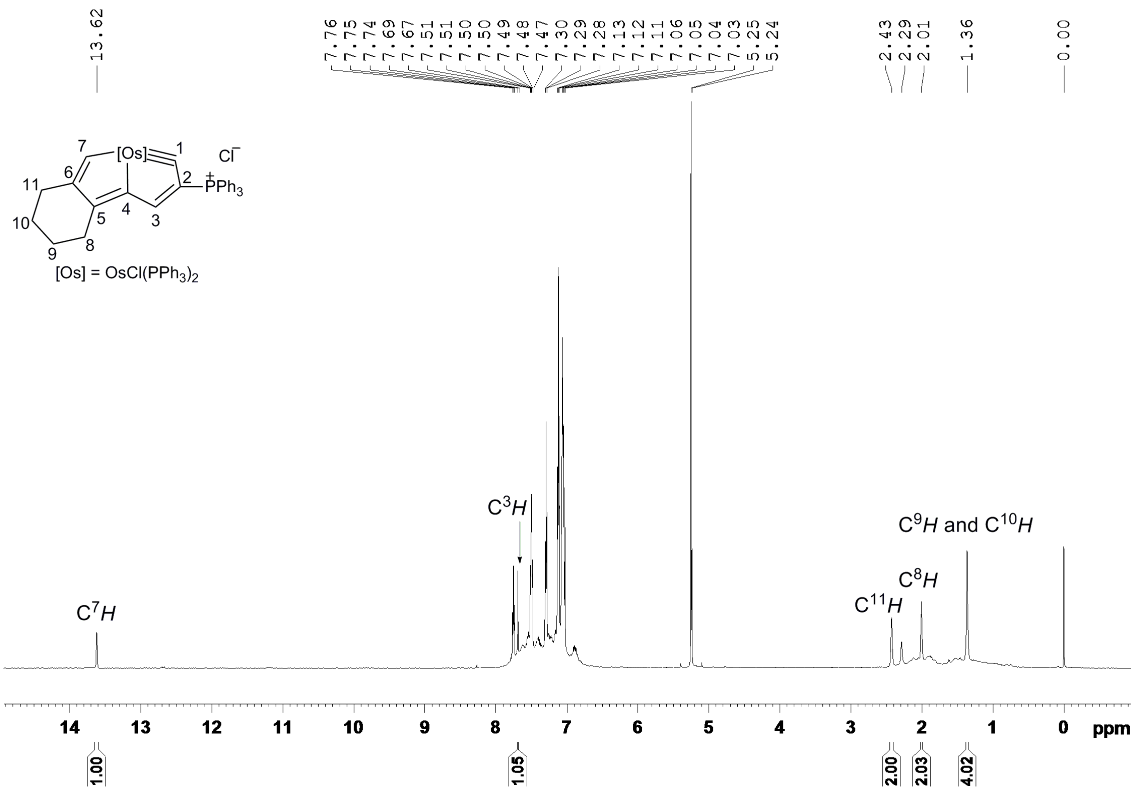

Supplementary Figure 77. ^{1}H NMR spectrum (600.1 MHz, CD_2Cl_2) of complex **4** at room temperature.

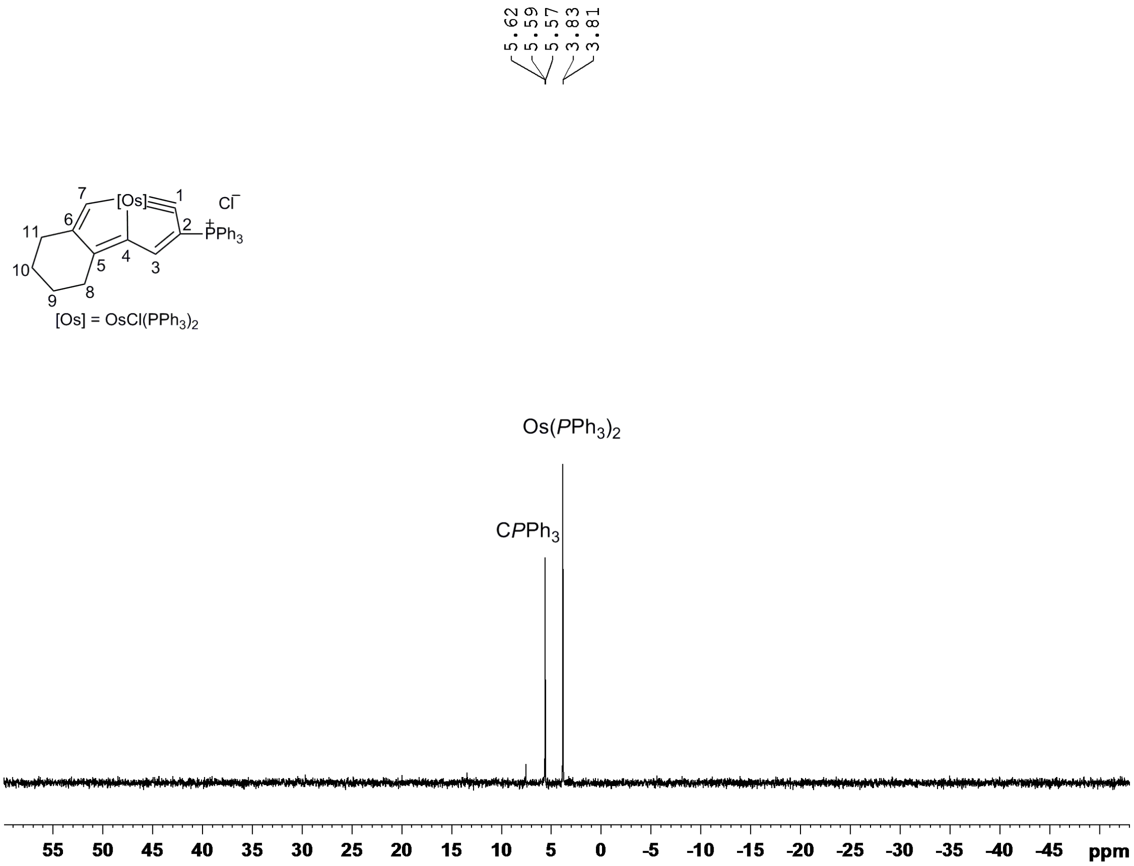

Supplementary Figure 78. ^{31}P NMR spectrum (242.9 MHz, CD_2Cl_2) of complex **4** at room temperature.

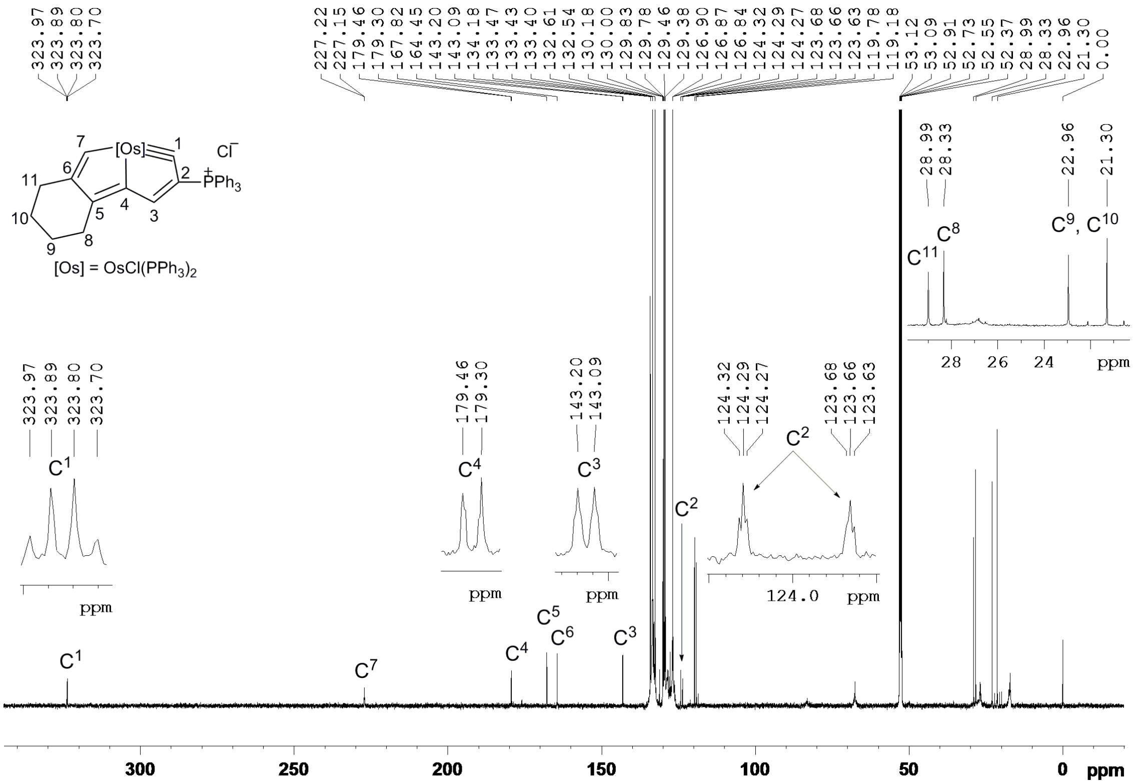

Supplementary Figure 79. ^{13}C NMR spectrum (150.9 MHz, CD_2Cl_2) of complex **4** at room temperature.

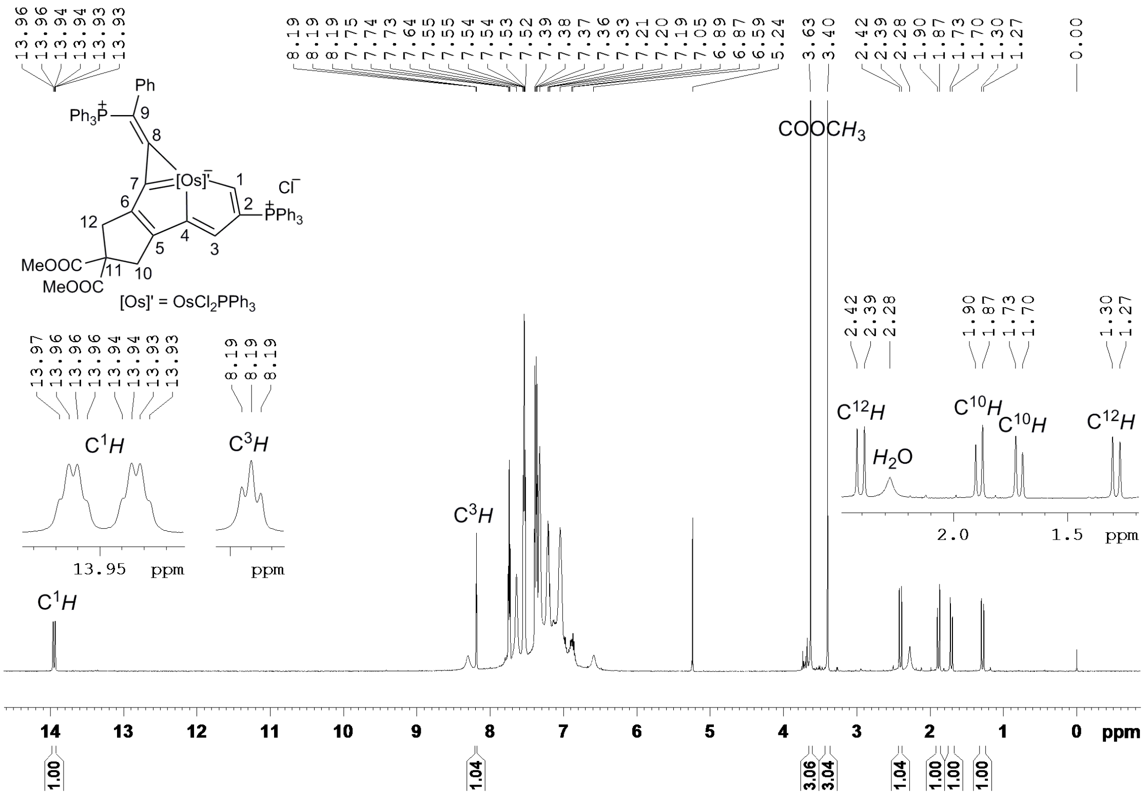

Supplementary Figure 80. ^{1}H NMR spectrum (600.1 MHz, CD_2Cl_2) of complex **5** at room temperature.

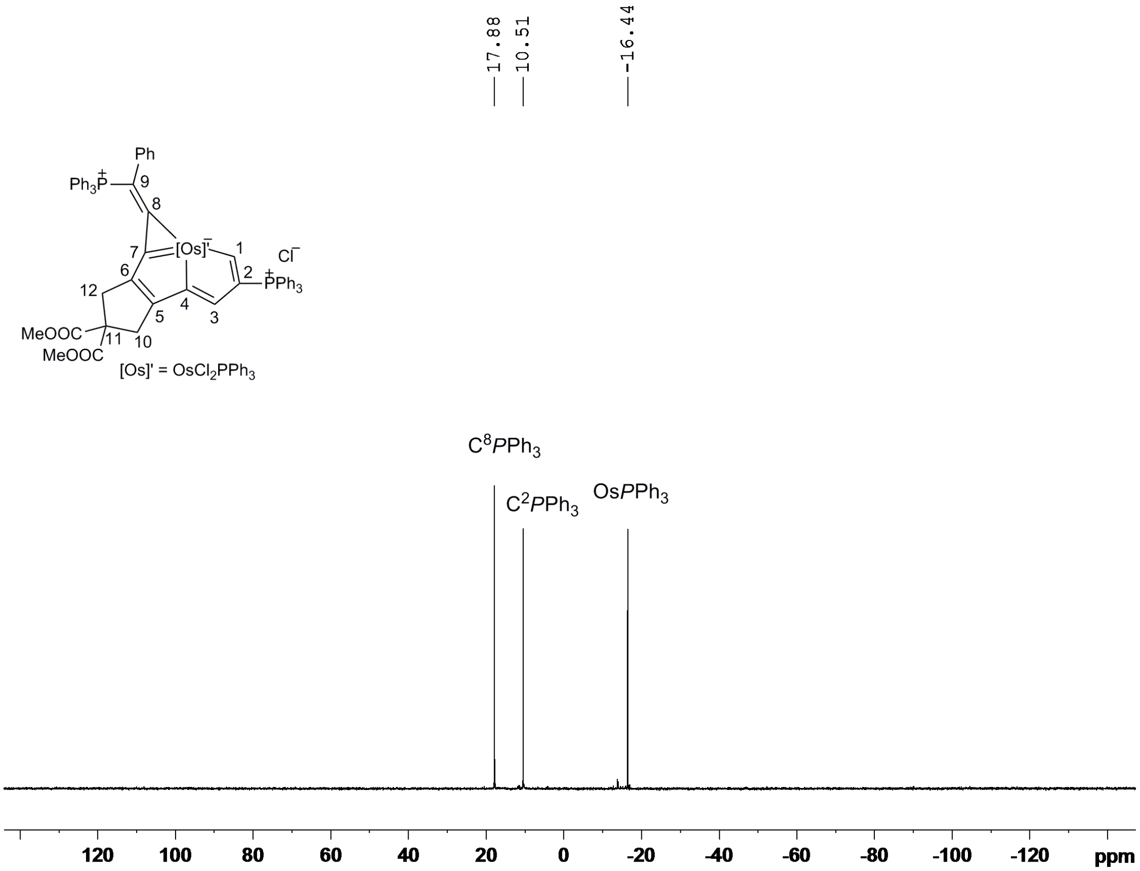

Supplementary Figure 81. ^{31}P NMR spectrum (242.9 MHz, CD_2Cl_2) of complex **5** at room temperature.

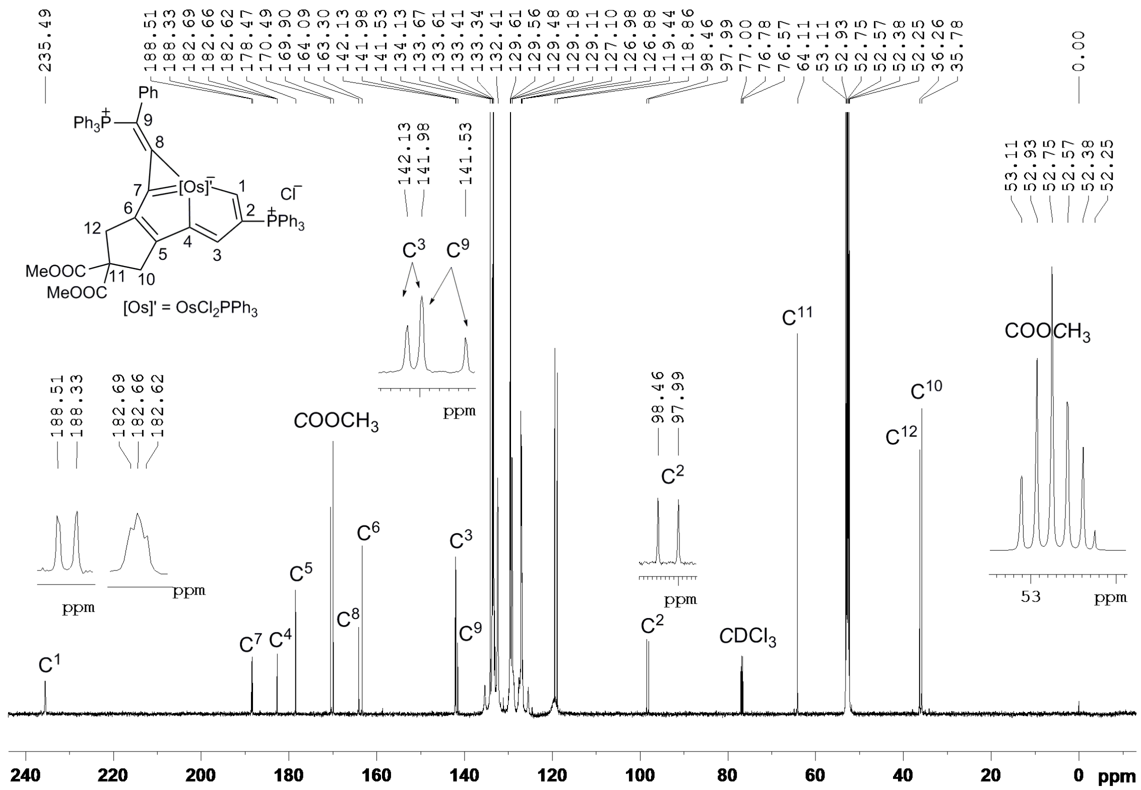

Supplementary Figure 82. ^{13}C NMR spectrum (150.5 MHz, CD_2Cl_2) of complex **5** at room temperature.

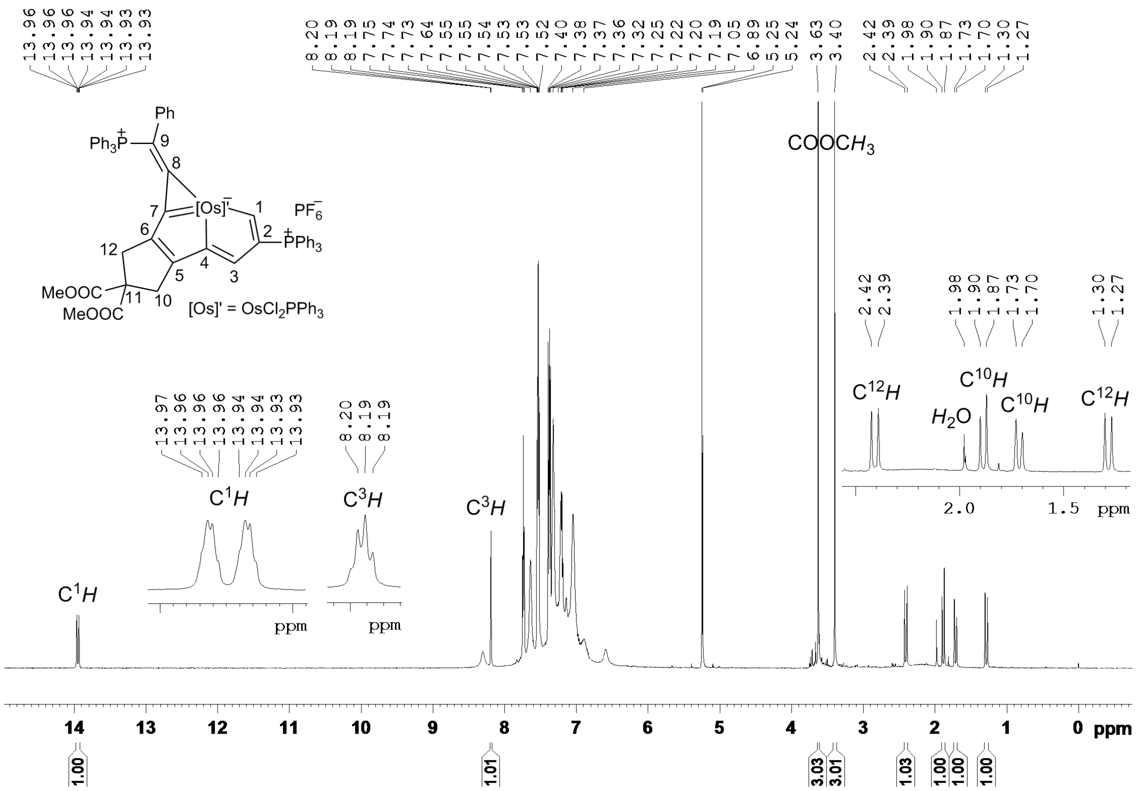

Supplementary Figure 83. ^{1}H NMR spectrum (600.1 MHz, CD_2Cl_2) of complex **5-PF$_6$** at room temperature.

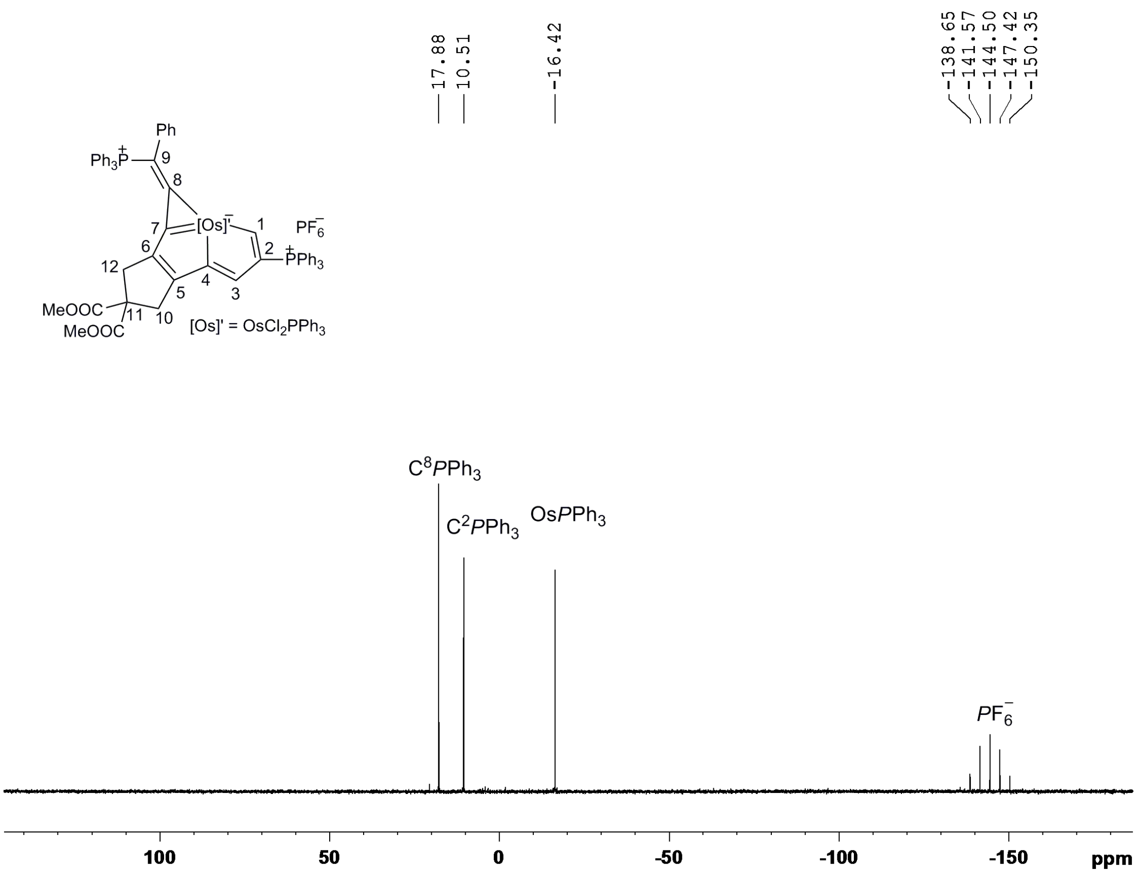

Supplementary Figure 84. ^{31}P NMR spectrum (242.9 MHz, CD_2Cl_2) of complex **5-PF$_6$** at room temperature.

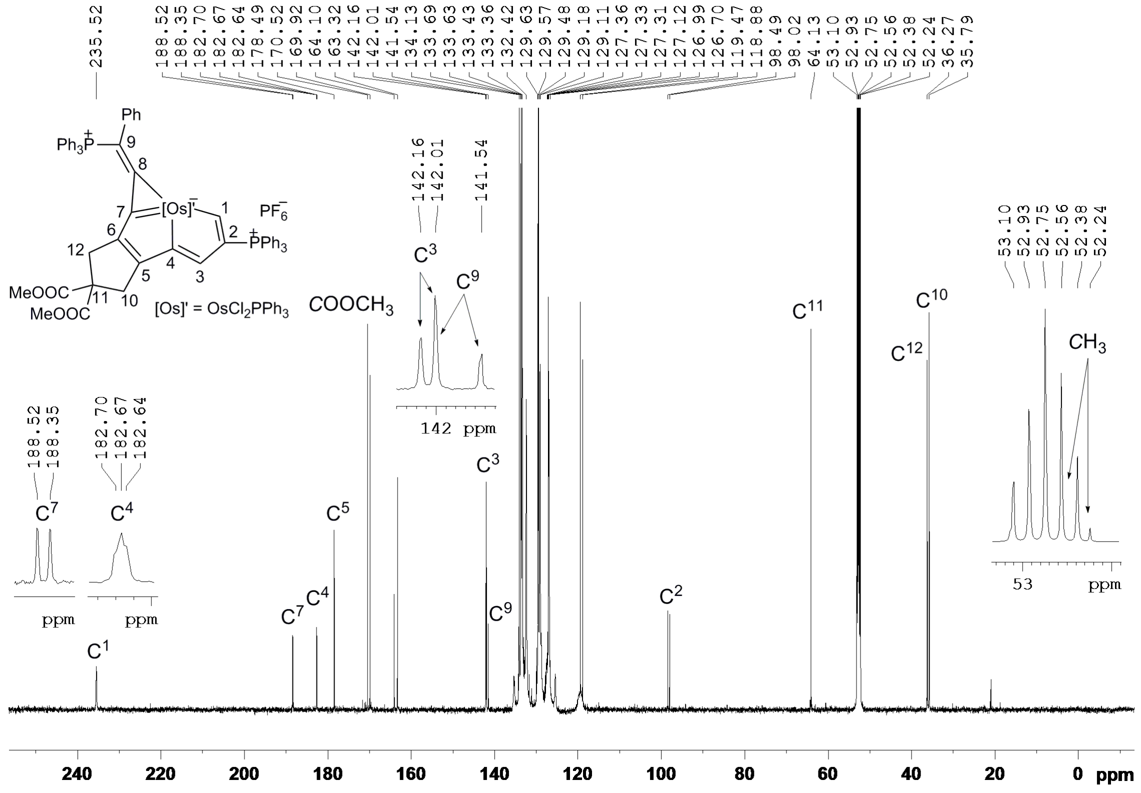

Supplementary Figure 85. ^{13}C NMR spectrum (150.5 MHz, CD_2Cl_2) of complex **5-PF$_6$** at room temperature.

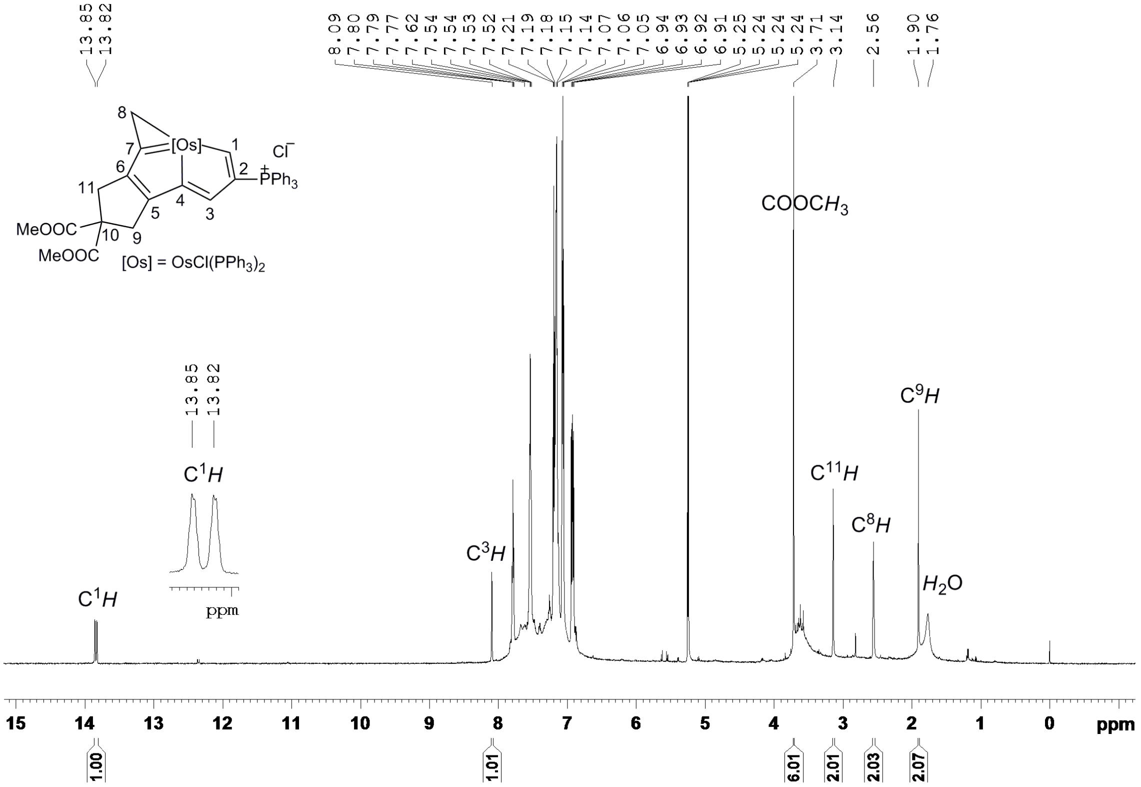

Supplementary Figure 86. ^{1}H NMR spectrum (600.1 MHz, CD_2Cl_2) of complex **6** at room temperature.

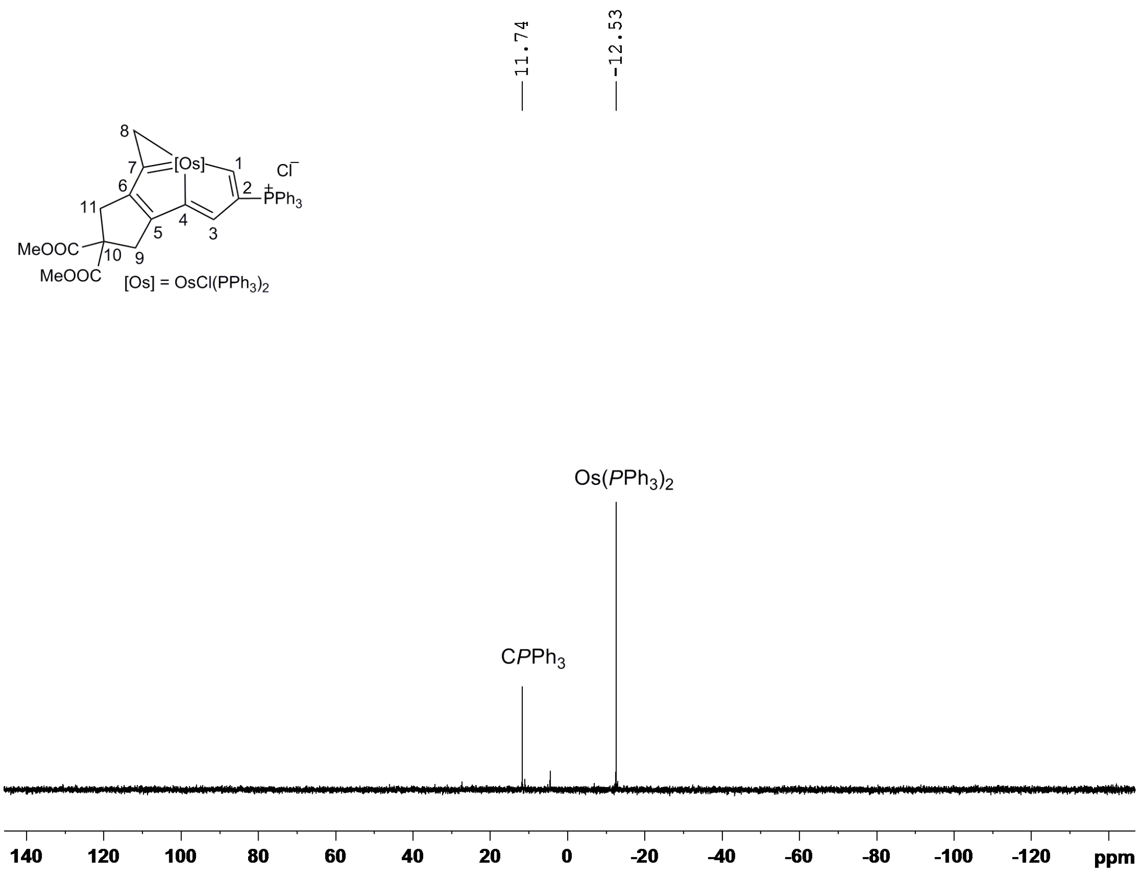

Supplementary Figure 87. ^{31}P NMR spectrum (242.9 MHz, CD_2Cl_2) of complex **6** at room temperature.

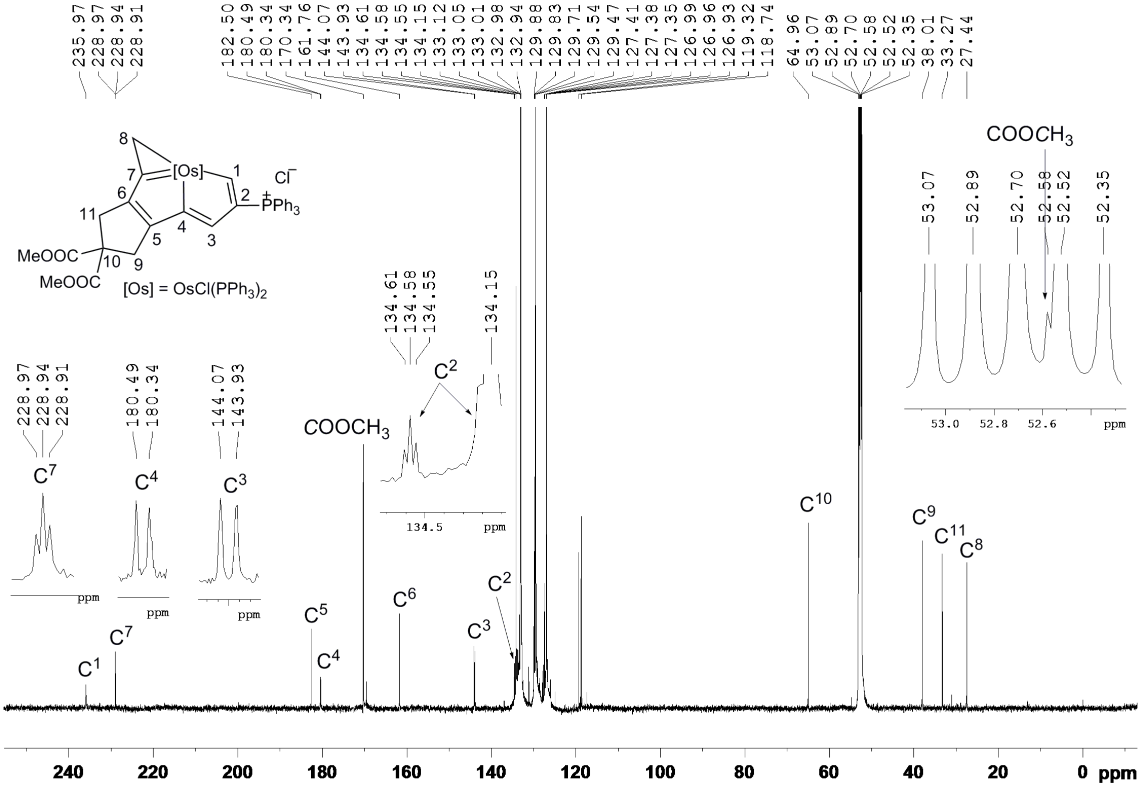

Supplementary Figure 88. ^{13}C NMR spectrum (150.9 MHz, CD_2Cl_2) of complex **6** at room temperature.

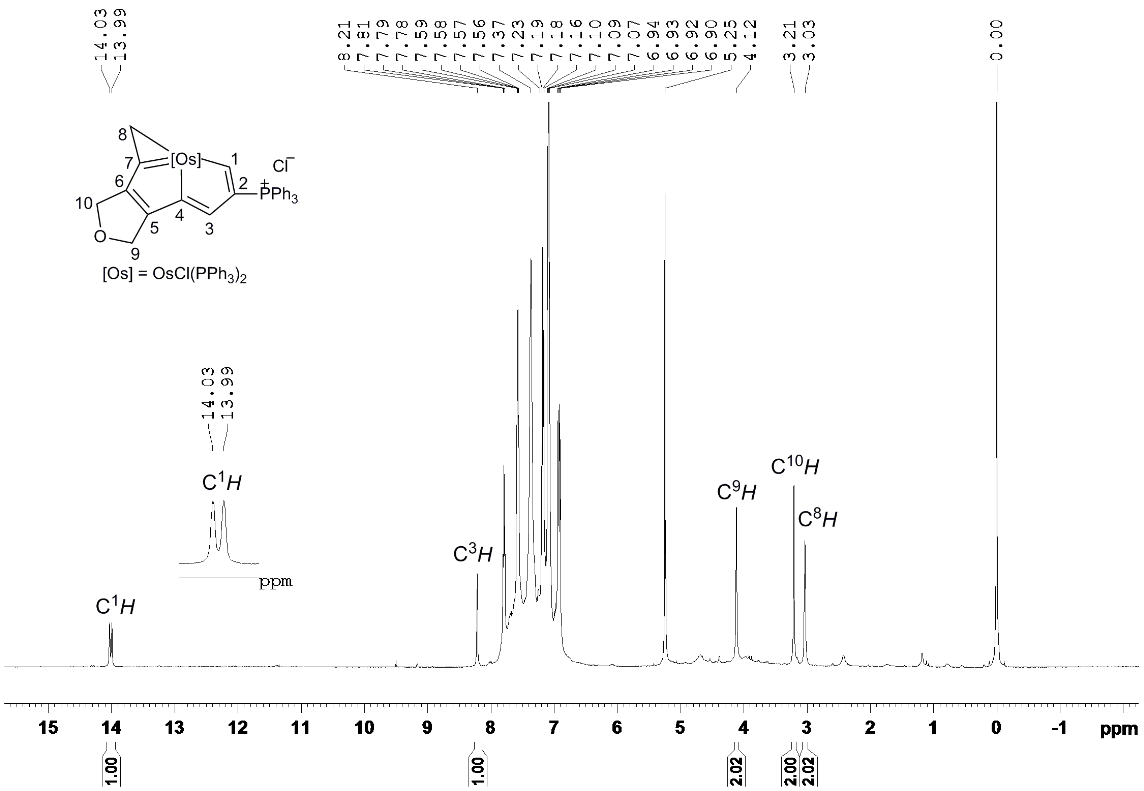

Supplementary Figure 89. ^{1}H NMR spectrum (500.2 MHz, CD_2Cl_2) of complex **7** at room temperature.

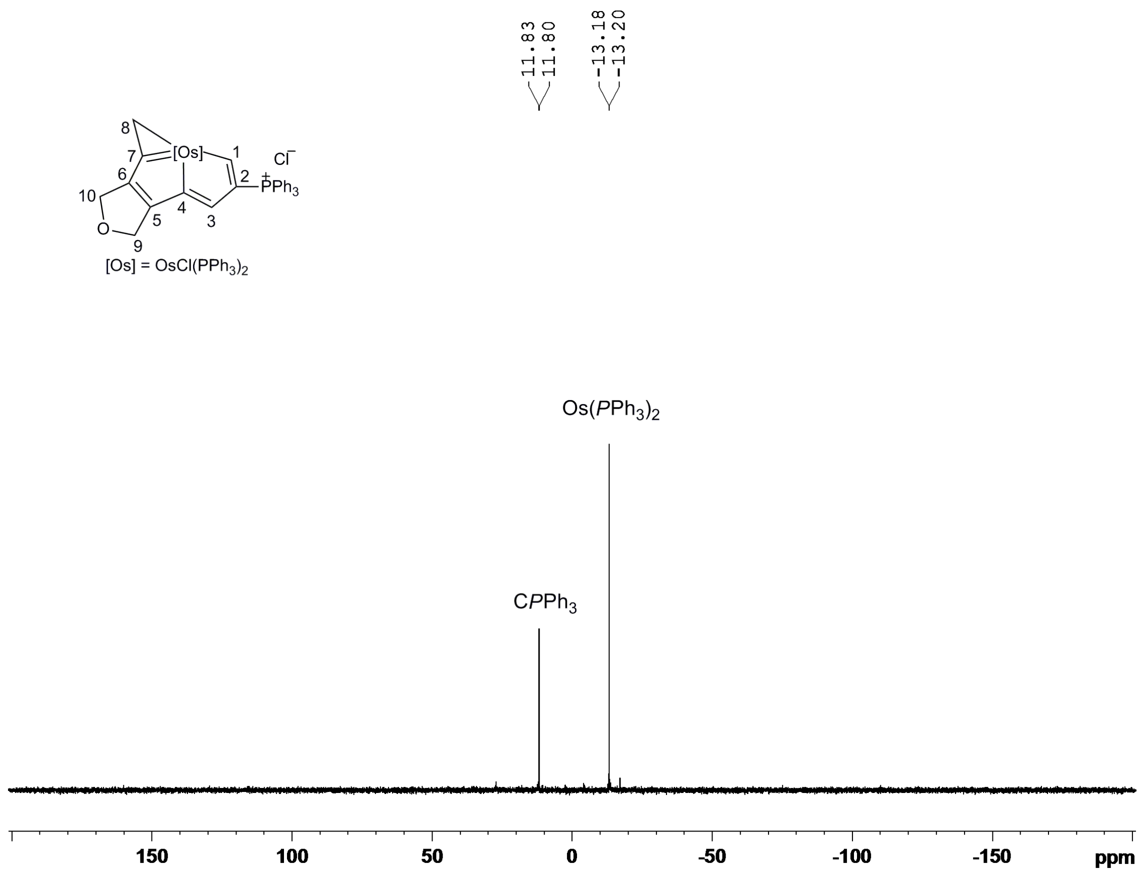

Supplementary Figure 90. ^{31}P NMR spectrum (202.5 MHz, CD_2Cl_2) of complex **7** at room temperature.

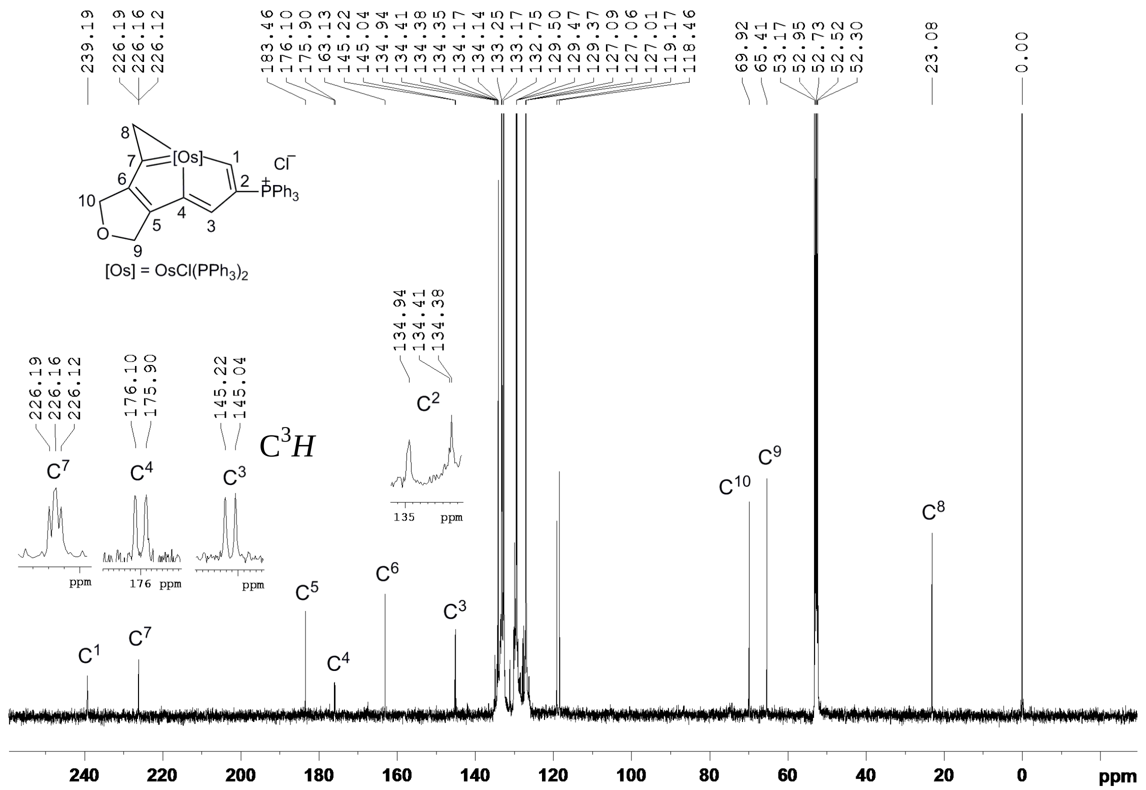

Supplementary Figure 91. ^{13}C NMR spectrum (125.8 MHz, CD_2Cl_2) of complex **7** at room temperature.

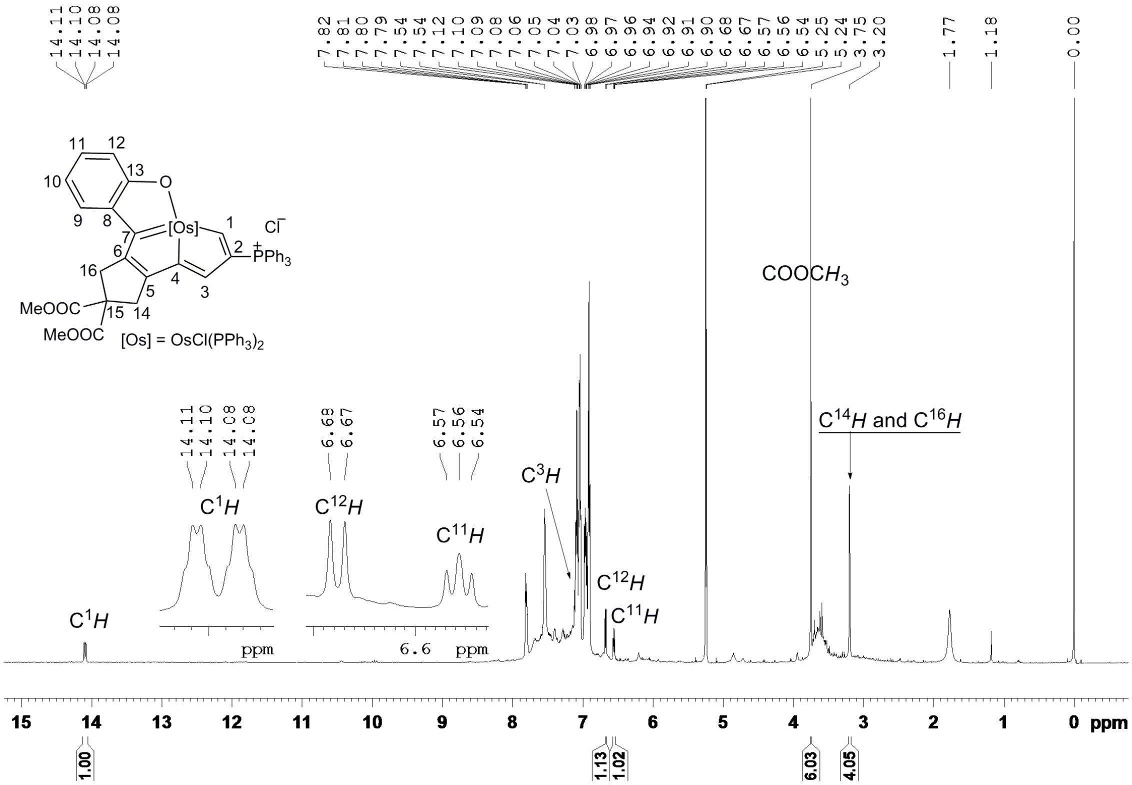

Supplementary Figure 92. ^{1}H NMR spectrum (600.1 MHz, CD_2Cl_2) of complex **8** at room temperature.

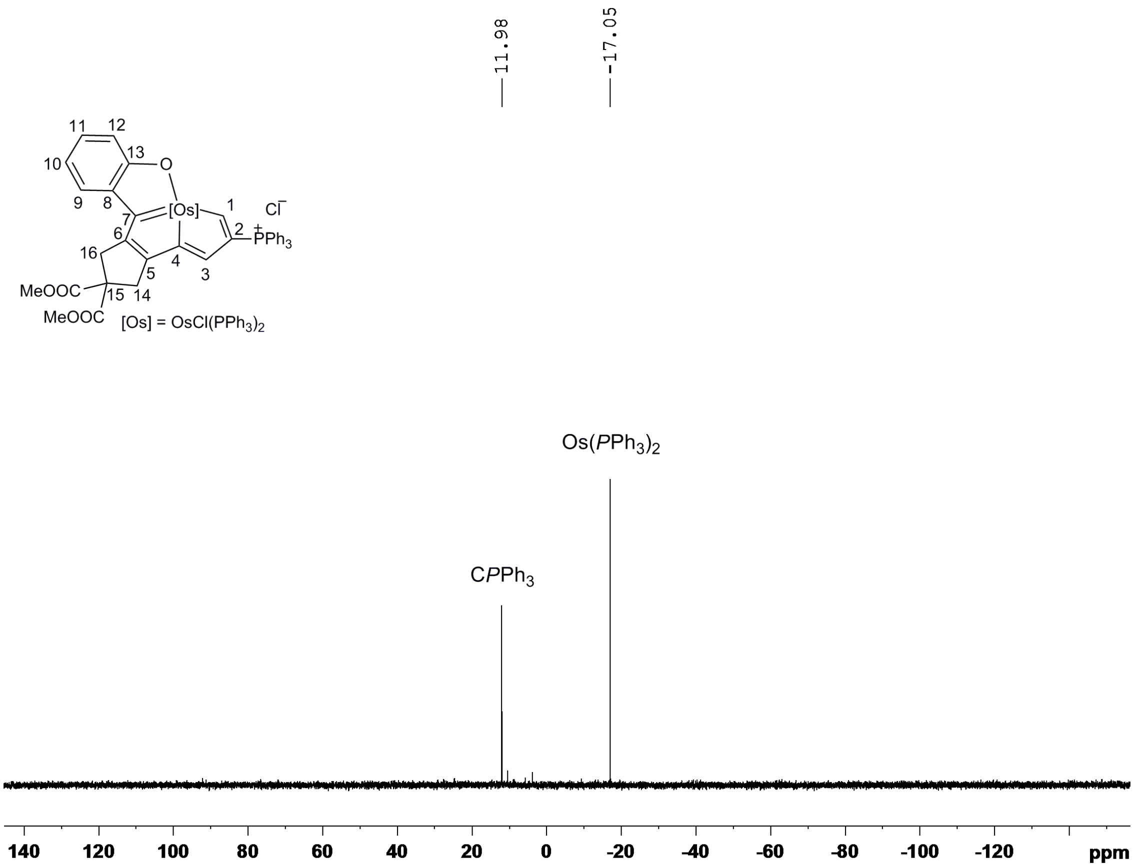

Supplementary Figure 93. ^{31}P NMR spectrum (242.9 MHz, CD_2Cl_2) of complex **8** at room temperature.

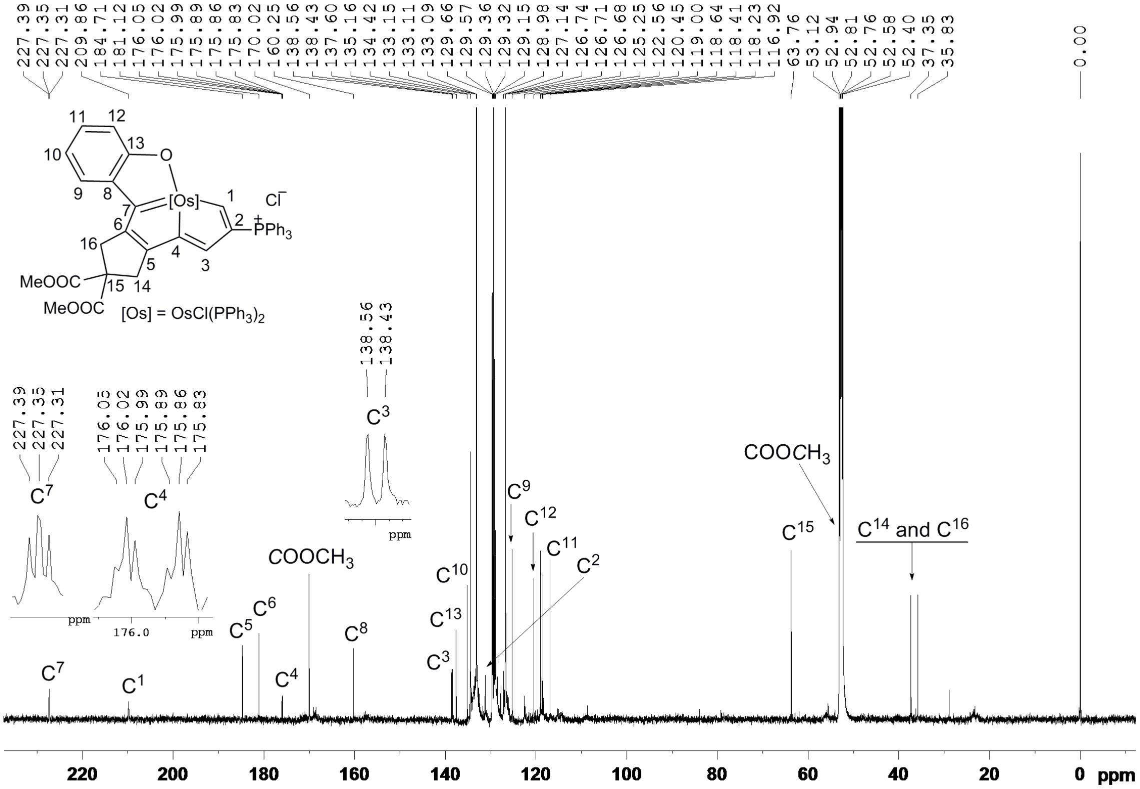

Supplementary Figure 94. ^{13}C NMR spectrum (150.9 MHz, CD_2Cl_2) of complex **8** at room temperature.

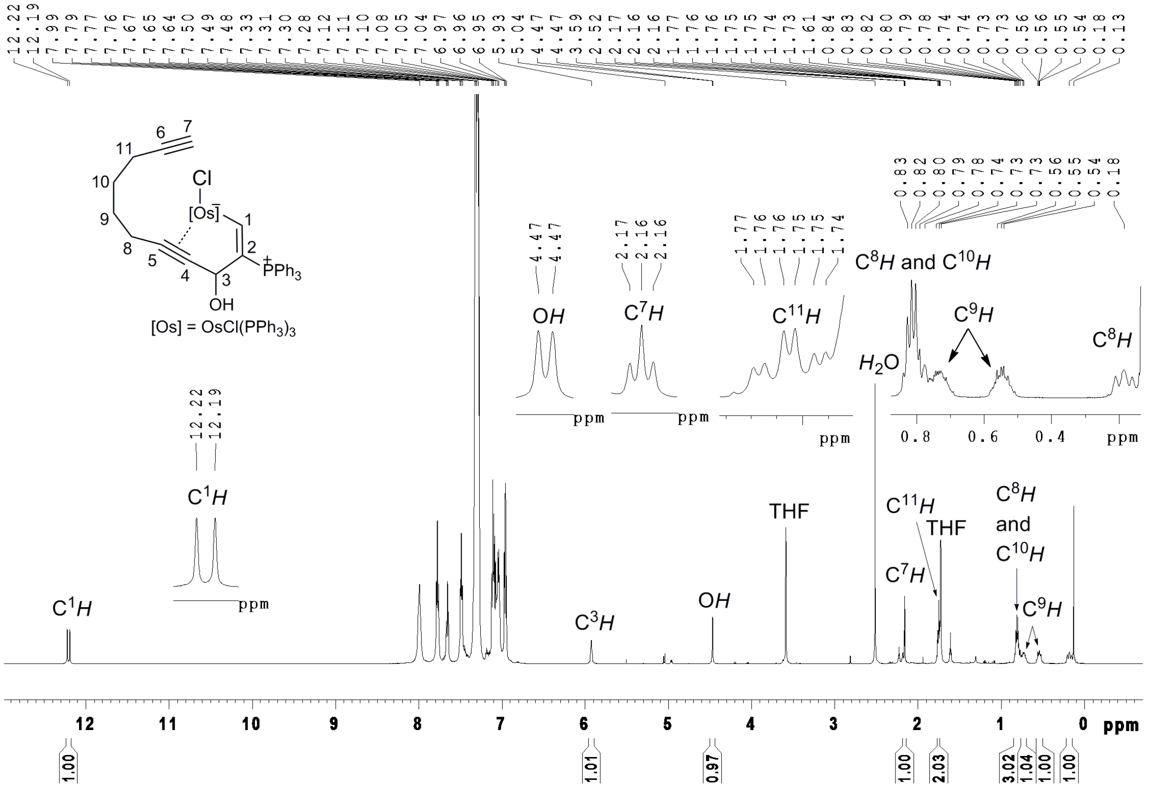

Supplementary Figure 95. In-situ ^{1}H NMR spectrum (600.1 MHz, THF-d8) of complex **9** at room temperature.

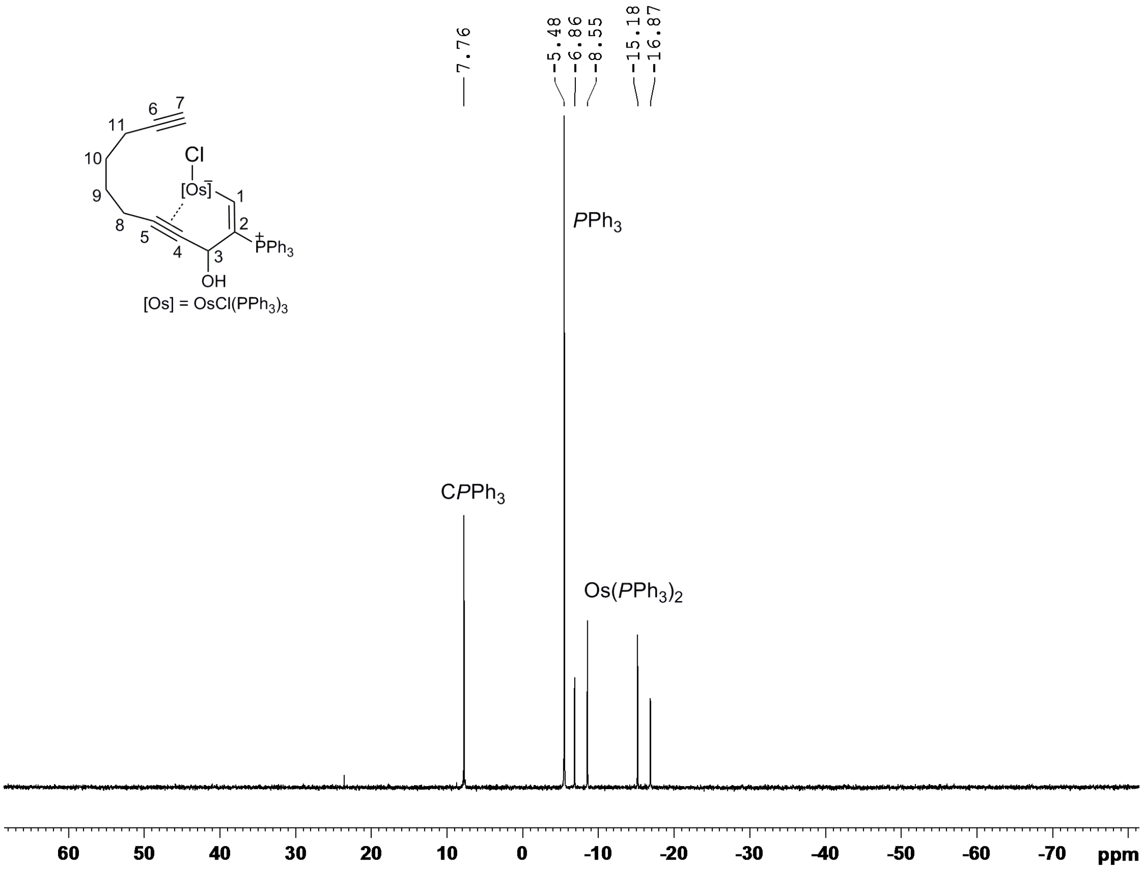

Supplementary Figure 96. In-situ ^{31}P NMR spectrum (242.9 MHz, THF-d8) of complex **9** at room temperature.

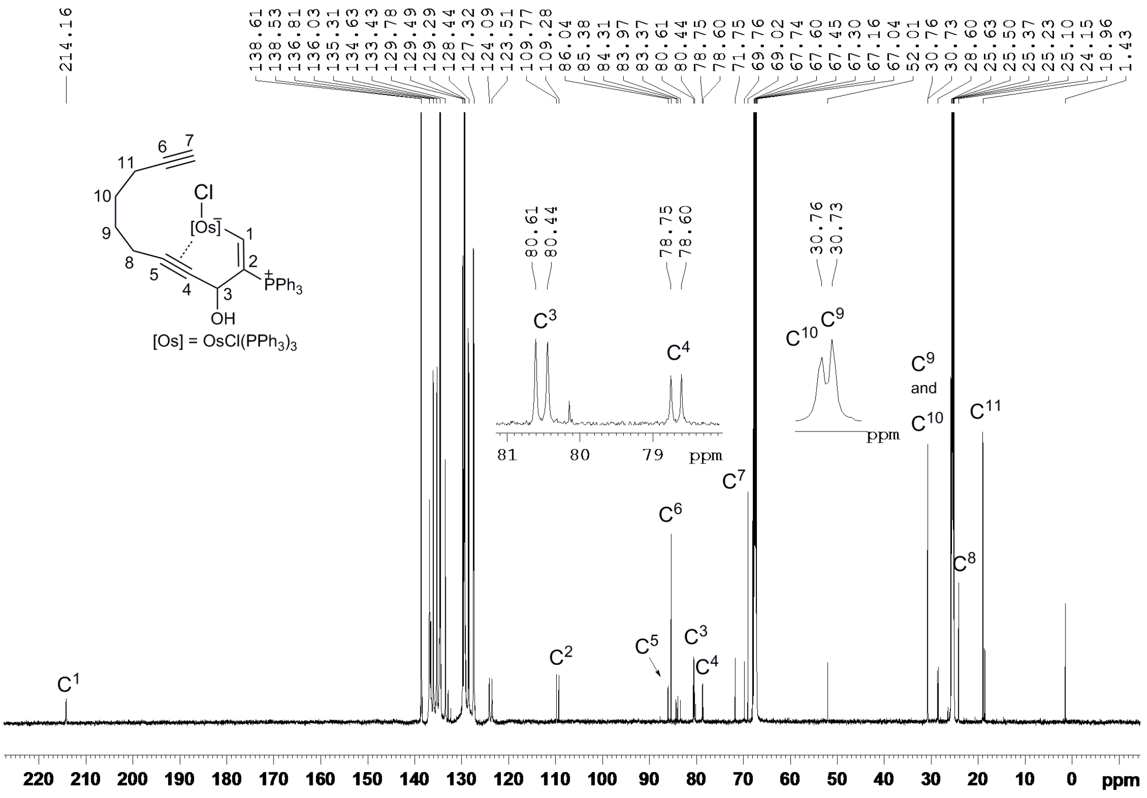

Supplementary Figure 97. In-situ ^{13}C NMR spectrum (150.9 MHz, THF-d8) of complex **9** at room temperature.

Supplementary Table 1. Thermal decomposition data of complexes **1**–**8** in the solid state[*].

Temperature (°C) / Complexes No.	30	40	60	80	100	120	140	160	180
**1**	●	●	●	●	▲	▲	■	–	–
**2**	●	●	●	■	–	–	–	–	–
**3**	●	▲	▲	■	–	–	–	–	–
**4**	●	●	▲	■	–	–	–	–	–
**5**	●	●	●	●	●	▲	▲	▲	■
**6**	●	●	●	●	▲	▲	▲	■	–
**7**	●	●	●	●	▲	▲	■	–	–
**8**	●	●	●	●	▲	▲	■	–	–

[*] All reactions were performed for 3 h in air.● = Stable; ▲ = Partly decomposed; ■ = Completely decomposed.

Supplementary Table 2. Crystal data and structure refinement for **1**, **5-PF$_6$** and **6**.

	**1** ·5CH$_2$Cl$_2$ ·1.5H$_2$O	**5-PF$_6$** ·1.5CH$_2$Cl$_2$ 0.75H$_2$O	2**6** ·3.5CH$_2$Cl$_2$ ·0.5H$_2$O
Empirical formula	$C_{73}H_{70}Cl_{12}O_{5.5}OsP_3$	$C_{77.5}H_{66.5}Cl_5F_6O_{4.75}OsP_4$	$C_{141.5}H_{126}Cl_{11}O_{8.5}Os_2P_6$
Mol. weight	1743.80	1679.13	2918.59
Temperature [K]	172.95(10)	173.00(10)	172.95(10)
Crystal size [mm]	0.30×0.10×0.10	0.40×0.30×0.30	0.748 ×0.387 ×0.035
Wavelength [Å]	0.71073	0.71073	0.71073
Crystal system	Triclinic	Triclinic	Triclinic
Space group	P-1	P-1	P-1
a [Å]	12.0632(4)	10.5960(4)	12.6947(6)
b [Å]	15.7535(7)	17.9231(7)	23.6189(17)
c [Å]	21.8701(8)	20.8821(8)	24.3434(14)
α [°]	103.803(3)	74.969(3)	114.652(6)
β [°]	90.169(3)	75.412(3)	99.582(4)
γ [°]	94.640(3)	84.350(3)	96.586(5)
V [Å^3]	4021.8(3)	3704.3(3)	6401.6(7)
Z	2	2	2
ρ_{calcd} [g cm^{-3}]	1.440	1.505	1.514
μ [mm^{-1}]	2.090	2.053	2.346
F(000)	1754.0	1689.0	2944.0
2θ range [°]	3.388 to 50	3.974 to 52.5	3.324 to 50
Coll. refl.	27334	31786	48556
Indep. refl.	14171	14939	22531
Obs. refl. [$I \geq 2\sigma(I)$]	11337	13869	16964
data/restraints/params	14171/70/923	14939/15/913	22531/684/1719
GOF on F^2	1.085	1.056	1.060
R_1/wR_2 [$I \geq 2\sigma(I)$]	0.0633 /0.1498	0.0343 /0.0864	0.0650/0.1495
R_1/wR_2 (all data)	0.0849 /0.1603	0.0382 /0.0881	0.0920 /0.1621
Largest peak/hole [e Å^{-3}]	1.40 /-0.75	1.46 /-1.26	2.14 /-1.21

Supplementary Table 3. Crystal data and structure refinement for **7** and **9**.

	**7**·3CH_2Cl_2·1.5H_2O	**9**·2C_4H_8O
Empirical formula	$C_{67}H_{62}Cl_8O_{2.5}OsP_3$	$C_{73}H_{73}Cl_2O_3OsP_3$
Mol. weight	1473.87	1352.32
Temperature [K]	153.0(10)	104(2)
Crystal size [mm]	0.4 × 0.2 × 0.2	0.2 × 0.1 × 0.02
Wavelength [Å]	0.71073	1.54184
Crystal system	Monoclinic	Monoclinic
Space group	$P2_1/c$	$P2_1/n$
a [Å]	12.3834(6)	15.0943(7)
b [Å]	17.8932(11)	24.7835(15)
c [Å]	29.580(2)	16.1256(7)
α [°]	90	90
β [°]	91.202(5)	98.130(4)
γ [°]	90	90
V [Å^3]	6552.9(7)	5971.7(5)
Z	4	4
ρ_{calcd} [g cm^{-3}]	1.494	1.504
μ [mm^{-1}]	2.389	6.004
F(000)	2964.0	2760.0
2θ range [°]	4.718 to 50	6.586 to 130.166
Coll. refl.	25650	24320
Indep. refl.	11519	10133
Obs. refl. [$I \geq 2\sigma(I)$]	9368	7706
data/restraints/params	11519/93/830	10133/60/740
GOF on F^2	1.056	1.006
R_1/wR_2 [$I \geq 2\sigma(I)$]	0.0476 /0.1066	0.0691 /0.1751
R_1/wR_2 (all data)	0.0631 /0.1133	0.0904 /0.1994
Largest peak/hole [e Å^{-3}]	1.31 /-0.79	3.55 /-1.93

Supplementary Methods

General Information

All alkyne syntheses were performed under an inert atmosphere (N_2) using standard Schlenk techniques. The transition metal complex syntheses were performed under an atmosphere of N_2 unless otherwise stated. Solvents were distilled from sodium/benzophenone (tetrahydrofuran, hexane and diethyl ether) or calcium hydride (dichloromethane) under N_2 prior to use. Non-distilled solvents were used in the reactions carried out in air. The starting materials, including dimethyl dipropargylmalonate[1], (trimethylsilyl)propiolaldehyde[2], dimethyl propargylmalonate,[3] (5-bromopenta-1,3-diyn-1-yl)benzene[4], dimethyl 2-(buta-2,3-dien-1-yl)-2-(prop-2-yn-1-yl)malonate[5], and buta-2,3-dien-1-ol[6] were synthesized according to previously published procedures. All other reagents were used as received from commercial sources without further purification. Column chromatography was performed on silica gel (100-200 or 200-300 mesh) in air. Nuclear magnetic resonance (NMR) spectroscopy was performed using a Bruker Advance II 300 spectrometer (^{1}H, 300.1 MHz; ^{13}C, 75.5 MHz; ^{31}P, 121.5 MHz), a Bruker Advance II 400 spectrometer (^{1}H, 400.1 MHz; ^{13}C, 100.6 MHz; ^{31}P, 162.0 MHz), a Bruker Advance III 500 spectrometer (^{1}H, 500.2 MHz; ^{13}C, 125.8 MHz; ^{31}P, 202.5 MHz) or a Bruker Ascend III 600 spectrometer (^{1}H, 600.1 MHz; ^{13}C, 150.9 MHz; ^{31}P, 242.9 MHz) at room temperature. The ^{1}H and ^{13}C NMR chemical shifts (δ) are relative to tetramethylsilane, and the ^{31}P NMR chemical shifts are relative to 85% H_3PO_4. The absolute values of the coupling constants are given in hertz (Hz). The multiplicities are abbreviated as s (singlet), d (doublet), t (triplet), q (quartet), m (multiplet), and br (broad). High-resolution mass spectrometry (HRMS) was conducted using a Bruker En Apex-Ultra 7.0 T FT-MS instrument. Elemental analyses were performed on a Vario EL III elemental analyser. Infrared spectra were obtained using a Nicolet AVATAR FTIR330 spectrometer using thin-film samples.

Single-Crystal X-Ray Diffraction Experiments

Single-crystal X-ray diffraction data were collected on an Oxford Gemini S Ultra CCD Area Detector with graphite-monochromated Mo Kα radiation (λ = 0.71073 Å) for **1**, **5-PF$_6$**, **6** and **7** and an Agilent SuperNova Dual system with mirror-monochromated Cu Kα radiation (λ = 1.54184 Å) for **9**. Absorption corrections were applied by using the program CrysAlis (Version 1.171.38.43[7] for **1**, **5-PF$_6$**, **6** and **7**, and Version 1.171.39.30t[8] for **9**; multi-scan). Using Olex2[9], structures were solved with the ShelXS[10] structure solution program using the Patterson Method (**1**, **5-PF$_6$**, **6** and **7)**, or the ShelXT[11] structure solution program using the Intrinsic Phasing Method (**9)**, and all of them were refined with the ShelXL[12] refinement package using least-squares minimization. The non-H atoms were refined anisotropically unless otherwise stated. The hydrogen atoms were introduced at their geometric positions and refined as riding atoms unless otherwise stated. Single crystals suitable for X-ray diffraction were grown from a solution of CH_2Cl_2 (**1**, **5-PF$_6$**, **6** and **7**) layered with hexane or a solution of tetrahydrofuran (**9**). A portion of the solvent molecules, dichloromethane in **1**, **5-PF$_6$**, **6**, and **7** and tetrahydrofuran in **9**, were disordered and refined with suitable restraints. A portion of the H_2O solvent molecules in **1**, **5-PF$_6$**, **6** and **7** were refined without the addition of H atoms. CCDC 1506325 (**1**), CCDC 1506339 (**5-PF$_6$**), CCDC 1548457 (**6**), CCDC 1548468 (**7**), and CCDC 1548472 (**9**) contain the supplementary crystallographic data for this paper. Further details on the crystal data, data collection, and refinements are provided in Supplementary Tables 2 and 3. These data can be obtained free of charge from the Cambridge Crystallographic Data Centre via www.ccdc.cam.ac.uk/data_request/cif.

Synthetic Methods

Multiyne Chain Synthesis:

Synthesis pathway for triyne L1.

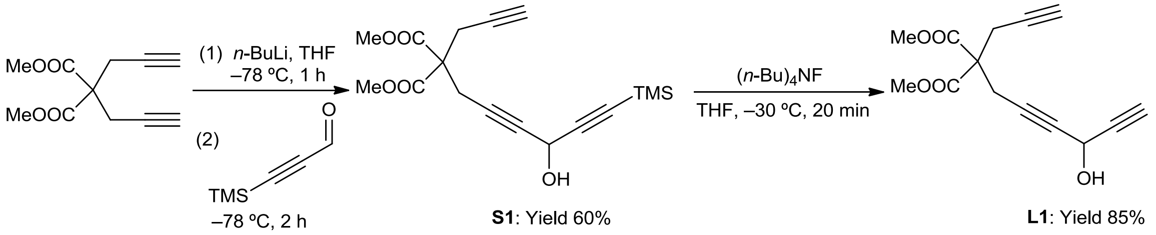

Preparation of compound S1: To an oven-dried flask containing dimethyl dipropargyl malonate (5.00 g, 24.0 mmol) and anhydrous tetrahydrofuran (200 mL), *n*-butyllithium (10.0 mL, 2.4 M in tetrahydrofuran, 24.0 mmol) was added dropwise over 45 min under N_2 at –78 ℃. After the reaction mixture was stirred for an additional 15 min, (trimethylsilyl)propiolaldehyde (3.55 mL, 24.0 mmol) was added dropwise at the same temperature. After 2 h, the reaction was quenched with saturated aqueous NH_4Cl (70 mL), and warmed to room temperature. The resulting mixture was extracted with Et_2O (3 × 250 mL), and the extract was washed with saturated brine (2 × 150 mL), dried over $MgSO_4$, and concentrated to dryness. The residue was chromatographed with hexane/EtOAc (5:1) to afford **S1** as a yellow oil. Yield: 4.82 g, 60%. ^{1}H NMR (500.2 MHz, $CDCl_3$): δ 5.03 (dt, J = 7.55 Hz, J = 2.01 Hz, 1H), 3.77 (s, 6H), 3.04 (d, J = 2.01 Hz, 2H), 2.98 (d, J = 2.60 Hz, 2H), 2.21 (br, 1H), 2.03 (t, J = 2.60 Hz, 1H), 0.18 ppm (s, 9H). ^{13}C NMR (125.8 MHz, $CDCl_3$): δ 168.96, 101.94, 89.19, 80.95, 79.27, 78.26, 71.73, 56.58, 53.05, 52.42, 22.91, 22.77, –0.45 ppm. HRMS-ESI (m/z) calcd for $[C_{17}H_{22}O_5SiNa]^+$ 357.1129 $[M + Na]^+$, found 357.1131. IR (thin film) 3480 (br), 3289 (s), 2957, 2900, 2237, 2175, 1741 (s), 1437, 1326, 1296, 1251, 1214, 1145, 1075, 1039, 974, 847, 762, 669 cm^{-1}.

Preparation of triyne L1: To a solution of **S1** (4.82 g, 14.4 mmol) in anhydrous tetrahydrofuran (150 mL), a solution of $(n\text{-Bu})_4NF$ in tetrahydrofuran (1.0 M, 21.6 mL, 21.6 mmol) was added at –30 ℃; the mixture was stirred at the same temperature until

the starting material disappeared (20 min). The mixture was then poured into a saturated aqueous solution of NH_4Cl (50 mL), extracted with Et_2O (3 × 150 mL), and washed with saturated brine (2 × 50 mL). The organic layer was dried over $MgSO_4$ and concentrated to dryness. The residue was chromatographed with hexane/EtOAc (3:1) to afford **L1** as a light yellow oil. Yield: 3.21 g, 85%. ^{1}H NMR (500.2 MHz, $CDCl_3$): δ 5.05 (ddt, J = 7.51 Hz, J = 2.25 Hz, J = 2.11 Hz, 1H), 3.76 (s, 6H), 3.02 (d, J = 2.11 Hz, 2H), 2.95 (d, J = 2.72 Hz, 2H), 2.81 (d, J = 7.51 Hz, 1H), 2.53 (d, J = 2.25 Hz, 1H), 2.03 ppm (t, J = 2.72 Hz, 1H). ^{13}C NMR (125.8 MHz, $CDCl_3$): δ 169.12, 169.10, 80.99, 80.78, 79.03, 78.19, 72.44, 72.10, 56.48, 53.25, 51.54, 22.84, 22.75 ppm. HRMS-ESI (m/z) calcd for $[C_{14}H_{14}O_5Na]^+$ 285.0733 $[M + Na]^+$, found 285.0734. IR (thin film) 3477 (br), 3240 (s), 2957, 2234, 2122, 2099, 1736 (s), 1437, 1327, 1297, 1215, 1144, 1074, 1026, 660 cm^{-1}.

Synthesis pathway for triyne L2.

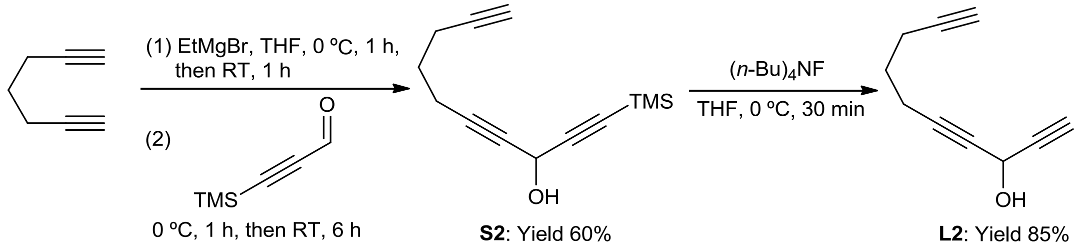

Preparation of compound S2: To an oven-dried flask containing 1,6-heptadiyne (5.00 mL, 4.02 g, 43.6 mmol) and anhydrous tetrahydrofuran (100 mL), EtMgBr (43.6 mL, 1.0 M in tetrahydrofuran, 43.6 mmol) was added dropwise over 1 h under N_2 at –0 ℃. After stirring for 1 h at room temperature, the reaction mixture was cooled to 0 ℃ and (trimethylsilyl)propiolaldehyde (6.44 mL, 43.6 mmol) was added in one portion. The resulting mixture was stirred for 1 h at 0 ℃, and then 6 h at room temperature. The reaction was quenched with saturated aqueous solutions of NH_4Cl (40 mL). The organic layer was separated, and the aqueous layer was extracted with Et_2O (3 × 150 mL). The extract was washed with brine (2 × 50 mL), dried with $MgSO_4$, and concentrated to dryness. The residue was chromatographed with hexane/EtOAc (6:1) to afford **S2** as a colorless oil. Yield: 5.71 g, 60%. ^{1}H NMR (500.2 MHz, $CDCl_3$): δ 5.09 (t, J = 1.70 Hz,

1H), 2.38 (td, J = 7.09 Hz, J = 1.70 Hz, 2H), 2.31 (td, J = 6.94 Hz, J = 2.51 Hz, 2H), 2.07 (br, 1H), 1.96 (t, J = 2.51, 1H), 1.76 (tt, J = 6.95 Hz, J = 7.09 Hz, 2H), 0.19 ppm (s, 9H). ^{13}C NMR (125.8 MHz, $CDCl_3$): δ 102.58, 89.17, 84.52, 83.53, 78.27, 69.15, 52.82, 27.31, 17.92, 17.67, –0.17 ppm. HRMS-ESI (m/z) calcd for $[C_{13}H_{18}OSiNa]^+$ 241.1019 $[M + Na]^+$, found 241.1023. IR (thin film) 3413 (br), 3294 (s), 2959, 2907, 2869, 2843, 2289, 2232, 2177, 2118, 1433, 1295, 1251, 1140, 1033, 998, 962, 845, 761, 637 cm^{-1}.

Preparation of triyne L2: To a solution of **S2** (5.00 g, 22.9 mmol) in anhydrous tetrahydrofuran (120 mL), a solution of $(n\text{-}Bu)_4NF$ in tetrahydrofuran (1.0 M, 27.5 mL, 27.5 mmol) was added at 0 ℃. The mixture was stirred at the same temperature until the starting material disappeared (30 min). The mixture was then poured into saturated aqueous solutions of NH_4Cl (50 mL), extracted with Et_2O (3 × 150 mL), and washed with saturated brine (2 × 50 mL). The organic layer was dried over $MgSO_4$ and concentrated to dryness. The residue was chromatographed with hexane/EtOAc (4:1) to afford **L2** as a colorless oil. Yield: 2.85 g, 85%. ^{1}H NMR (500.2 MHz, $CDCl_3$): δ 5.10 (dt, J = 2.21 Hz, J = 1.90 Hz, 1H), 2.55 (d, J = 2.21 Hz, 1H), 2.38 (td, J = 7.03 Hz, J = 1.90 Hz, 2H), 2.31 (td, J = 7.05 Hz, J = 2.56 Hz, 2H), 2.16 (br, 1H), 1.97 (t, J = 2.56 Hz, 1H), 1.75 ppm (tt, J = 7.05 Hz, J = 7.03 Hz, 2H). ^{13}C NMR (125.8 MHz, $CDCl_3$): δ 84.73, 83.49, 81.48, 77.88, 72.45, 69.24, 52.11, 27.18, 17.78, 17.63 ppm. HRMS-ESI (m/z) calcd for $[C_{10}H_{10}ONa]^+$ 169.0624 $[M + Na]^+$, found 169.0626. IR (thin film) 3381 (br), 3292 (s), 2940, 2910, 2869, 2841, 2287, 2225, 2118, 2099, 1453, 1432, 1300, 1187, 1139, 1018, 917, 794, 747, 645 cm^{-1}.

Synthesis pathway for triyne L3.

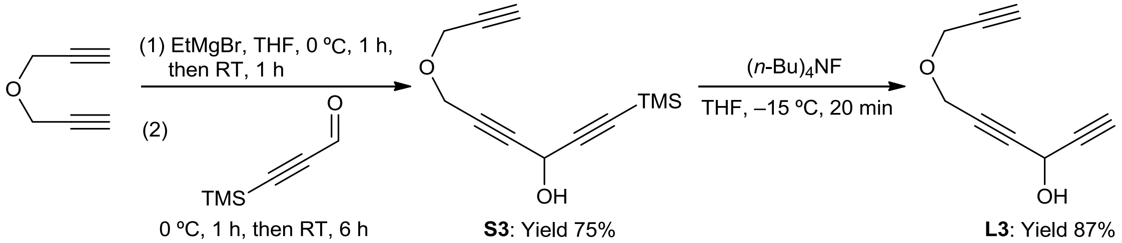

Preparation of compound S3: To an oven-dried flask containing propargyl ether (5.47

mL, 5.00 g, 53.1 mmol) and anhydrous tetrahydrofuran (130 mL), EtMgBr (53.1 mL, 1.0 M in tetrahydrofuran, 53.1 mmol) was added dropwise over 1 h under N_2 at 0 ℃. After stirring for 1 h at room temperature, the reaction mixture was cooled to 0 ℃ and (trimethylsilyl)propiolaldehyde (7.85 mL, 53.1 mmol) was added in one portion. The resulting mixture was stirred for 1 h at 0 ℃, and then 6 h at room temperature. The reaction was quenched with saturated aqueous solutions of NH_4Cl (50 mL). The organic layer was separated, and the aqueous layer was extracted with Et_2O (3 × 150 mL). The extract was washed with brine (2 × 50 mL), dried with $MgSO_4$, and concentrated to dryness. The residue was chromatographed with hexane/EtOAc (5:1) to afford **S3** as a light yellow oil. Yield: 8.78 g, 75%. ^{1}H NMR (300.1 MHz, $CDCl_3$): δ 5.14 (t, J = 1.67 Hz, 1H), 4.31 (d, J = 1.67 Hz, 1H), 4.24 (d, J = 2.37 Hz, 2H), 2.47 (br, 1H), 2.46 (t, J = 2.37 Hz, 1H), 0.17 (s, 9H). ^{13}C NMR (75.5 MHz, $CDCl_3$): δ 101.60, 89.95, 84.19, 79.75, 78.86, 75.40, 56.82, 56.75, 52.60, –0.22. HRMS-ESI (m/z) calcd for $[C_{12}H_{16}O_2SiNa]^+$ 243.0812 $[M + Na]^+$, found 243.0805. IR (thin film) 3404 (br), 3291 (s), 2960, 2901, 2858, 2178, 2118, 1444, 1346, 1295, 1251, 1129, 1082, 1038, 966, 846, 762, 673 cm^{-1}.

Preparation of triyne L3: To a solution of **S3** (6.00 g, 27.2 mmol) in anhydrous tetrahydrofuran (150 mL), a solution of $(n\text{-Bu})_4NF$ in tetrahydrofuran (1.0 M, 32.7 mL, 32.7 mmol) was added dropwise at –15 ℃. The mixture was stirred at the same temperature until the starting material disappeared (20 min). The mixture was then poured into saturated aqueous solutions of NH_4Cl (50 mL), extracted with Et_2O (3 × 150 mL), and washed with saturated brine (2 × 50 mL). The organic layer was dried over $MgSO_4$ and concentrated to dryness. The residue was chromatographed with hexane/EtOAc (4:1) to afford **L3** as a light yellow oil. Yield: 3.51 g, 87%. ^{1}H NMR (500.2 MHz, $CDCl_3$): δ 5.16 (s, 1H), 4.31 (d, J = 1.52 Hz, 1H), 4.25 (d, J = 2.25 Hz, 2H), 2.80 (br, 1H), 2.57 (d, J = 2.25 Hz, 1H), 2.47 ppm (t, J = 2.25 Hz, 1H). ^{13}C NMR (125.8 MHz, $CDCl_3$): δ 83.81, 80.69, 80.09, 78.82, 75.47, 73.13, 56.87, 56.76, 52.05 ppm. HRMS-ESI (m/z) calcd for $[C_9H_8O_2Na]^+$ 171.0417 $[M + Na]^+$, found 171.0412. IR (thin

film) 3386 (br), 3291 (s), 2954, 2906, 2858, 2120, 1443, 1384, 1348, 1299, 1265, 1128, 1080, 1026, 931, 884, 801, 652 cm^{-1}.

Synthesis pathway for triyne L4.

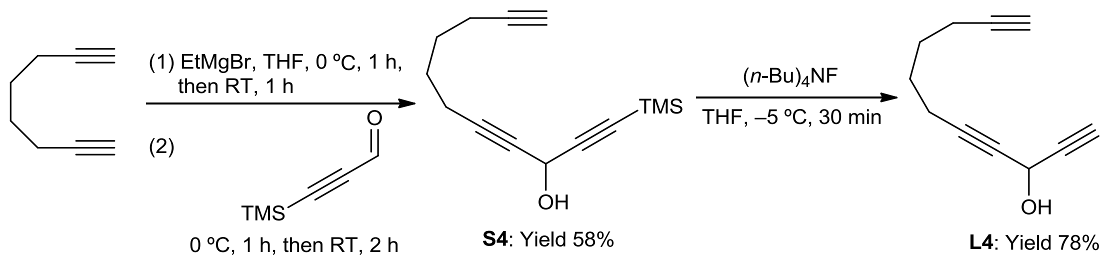

Preparation of compound S4: To an oven-dried flask containing 1,7-octadiyne (5 mL, 4.00 g, 37.7 mmol) and anhydrous tetrahydrofuran (120 mL), EtMgBr (37.7 mL, 1.0 M in tetrahydrofuran, 37.7 mmol) was added dropwise over 1 h under N_2 at 0 ℃. After stirring for 1 h at room temperature, the reaction mixture was cooled to 0 ℃, and (trimethylsilyl)propiolaldehyde (5.57 mL, 37.7 mmol) was added in one portion. The resulting mixture was stirred for 1 h at 0 ℃, and then 2 h at room temperature. The reaction was quenched with saturated aqueous solutions of NH_4Cl (50 mL). The organic layer was separated, and the aqueous layer was extracted with Et_2O (3 × 150 mL). The extract was washed with brine (2 × 50 mL), dried with $MgSO_4$, and concentrated to dryness. The residue was chromatographed with hexane/EtOAc (6:1) to afford **S4** as a colorless oil. Yield: 5.08 g, 58%. ^{1}H NMR (400.1 MHz, $CDCl_3$): δ 5.07 (dt, J = 7.29 Hz, J = 1.88 Hz, 1H), 2.33 (d, J = 7.29 Hz, 1H), 2.25 (td, J = 6.63 Hz, J = 1.88 Hz, 2H), 2.20 (td, J = 6.67 Hz, J = 2.83 Hz, 2H), 1.94 (t, J = 2.83 Hz, 1H), 1.63 (m, 4H), 0.17 (s, 9H). ^{13}C NMR (100.6 MHz, $CDCl_3$): δ 102.70, 88.90, 85.05, 84.17, 77.92, 68.77, 52.69, 27.51, 27.24, 18.33, 18.00, –0.22. HRMS-ESI (m/z) calcd for $[C_{14}H_{20}OSiNa]^+$ 255.1176 $[M + Na]^+$, found 255.1176. IR (thin film) 3413 (br), 3294 (s), 2952, 2904, 2865, 2289, 2232, 2177, 2117, 1431, 1329, 1297, 1251, 1139, 1034, 993, 953, 846, 761, 635 cm^{-1}.

Preparation of triyne L4: To a solution of **S4** (3.00 g, 12.9 mmol) in anhydrous tetrahydrofuran (100 mL), a solution of $(n\text{-Bu})_4NF$ in tetrahydrofuran (1.0 M, 15.5 mL, 15.5 mmol) was added at –5 ℃. The mixture was stirred at the same temperature until the

starting material disappeared (30 min). The mixture was then poured into a saturated aqueous solution of NH_4Cl (30 mL), extracted with Et_2O (3 × 150 mL), and washed with saturated brine (2 × 50 mL). The organic layer was dried over $MgSO_4$ and concentrated to dryness. The residue was chromatographed with hexane/EtOAc (4:1) to afford **L4** as a light yellow oil. Yield: 1.61 g, 78%. ^{1}H NMR (500.2 MHz, $CDCl_3$): δ 5.06 (ddt, *J* = 7.09 Hz, *J* = 2.30 Hz, *J* = 1.90 Hz, 1H), 2. 81 (br, 1H), 2.53 (d, *J* = 2.30 Hz, 1H), 2.22 (td, *J* = 6.44 Hz, *J* = 1.90 Hz, 2H), 2.17 (td, *J* = 6.61 Hz, *J* = 2.65 Hz, 2H), 1.93 (t, *J* = 2.65 Hz, 1H), 1.59 ppm (m, 4H). ^{13}C NMR (125.8 MHz, $CDCl_3$): δ 85.32, 84.20, 81.50, 77.49, 72.37, 68.83, 52.00, 27.44, 27.17, 18.22, 17.95 ppm. HRMS-ESI (m/z) calcd for $[C_{11}H_{12}ONa]^+$ 183.0780 $[M + Na]^+$, found 183.0785. IR (thin film) 3396 (br), 3292 (s), 2946, 2865, 2287, 2228, 2118, 2099, 1459, 1431, 1380, 1329, 1300, 1138, 1018, 928, 646 cm^{-1}.

Synthesis pathway for tetrayne L5.

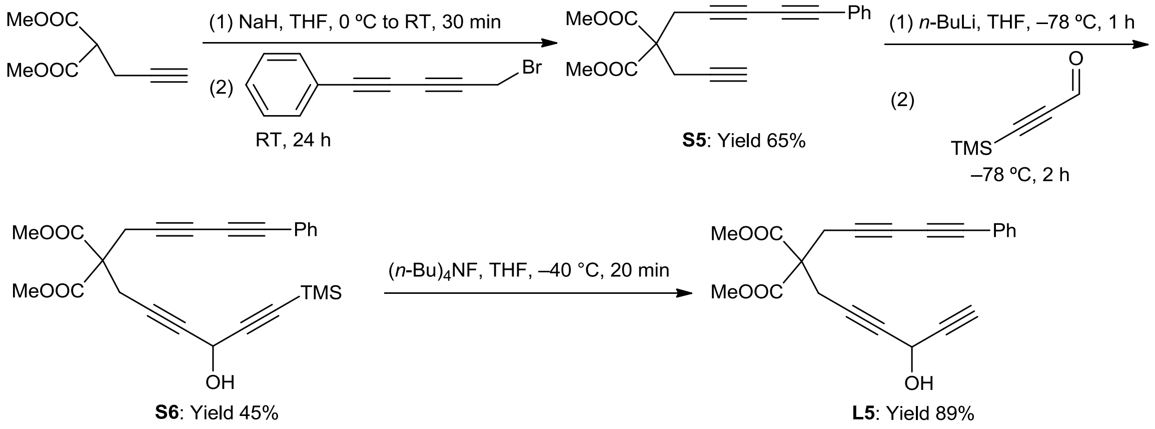

Preparation of compound S5: To an oven-dried three-necked flask, NaH (423 mg, 60% dispersion in mineral oil, 10.6 mmol) and anhydrous tetrahydrofuran (50 mL) was added sequentially under a N_2 atmosphere. After cooling to 0 ℃, a solution of dimethyl propargylmalonate (1.80 g, 10.6 mmol) in anhydrous tetrahydrofuran (10 mL) was added dropwise. The reaction mixture was the stirred at room temperature for 30 min, and (5-bromopenta-1,3-diyn-1-yl)benzene (2.32 g, 10.6 mmol) was added dropwise at room temperature. After stirring for 24 h at room temperature, the reaction was quenched with

saturated aqueous NH_4Cl (30 mL) at 0 ℃, and then warmed to room temperature. The resulting mixture was extracted with Et_2O (3 × 50 mL), and the extract was washed with saturated brine (2 × 50 mL), dried over $MgSO_4$, and concentrated to dryness. The residue was chromatographed with hexane/EtOAc (10:1) to afford dimethyl 2-(5-phenylpenta-2,4-diyn-1-yl)-2-(prop-2-yn-1-yl)malonate **S5** as a white solid. Yield: 2.12 g, 65%. ^{1}H NMR (500.2 MHz, $CDCl_3$): *δ* 7.20-7.39 (m, 5H), 3.69 (s, 6H), 3.10 (s, 2H), 2.94 (d, *J* = 2.70 Hz, 2H), 1.99 ppm (t, *J* = 2.70 Hz, 1H). ^{13}C NMR (125.8 MHz, $CDCl_3$): *δ* 168.83, 132.60, 129.24, 128.46, 121.58, 78.20, 77.91, 75.95, 73.88, 72.17, 68.29, 56.60, 53.29, 23.95, 23.04 ppm. HRMS-ESI (m/z) calcd for $[C_{19}H_{16}O_4Na]^+$ 331.0941 $[M + Na]^+$, found 331.0943. IR (thin film) 3295 (s), 2955, 2248, 2124, 1742 (s), 1436, 1291, 1213, 1072, 1055, 757, 689, 654 cm^{-1}.

Preparation of compound S6: To an oven-dried flask containing **S5** (2.56 g, 8.30 mmol) and anhydrous tetrahydrofuran (100 mL), *n*-butyllithium (3.46 mL, 2.4 M in tetrahydrofuran, 8.30 mmol) was added dropwise over 45 min under N_2 at –78 ℃. After stirring the reaction mixture for an additional 15 min, (trimethylsilyl)propiolaldehyde (1.54 mL, 10.4 mmol) was added dropwise at the same temperature. After 2 h, the reaction was quenched with saturated aqueous NH_4Cl (50 mL), and then warmed to room temperature. The resulting mixture was extracted with Et_2O (3 × 50 mL), and the extract was washed with saturated brine (2 × 50 mL), dried over $MgSO_4$, and concentrated to dryness. The residue was chromatographed with hexane/EtOAc (3:1) to afford **S6** as a light yellow solid. Yield: 1.62 g, 45%. ^{1}H NMR (500.2 MHz, $CDCl_3$): *δ* 7.31-7.50 (m, 5H), 5.07 (dt, *J* = 7.61 Hz, *J* = 2.02 Hz, 1H), 3.81 (s, 6H), 3.19 (s, 2H), 3.09 (d, *J* = 2.02 Hz, 2H), 2.38 (d, *J* = 7.61 Hz, 1H), 0.21 ppm (s, 9H). ^{13}C NMR (125.8 MHz, $CDCl_3$): *δ* 168.86, 132.57, 129.16, 128.38, 121.56, 101.99, 89.28, 81.30, 79.12, 77.82, 75.92, 73.81, 68.30, 56.73, 53.29, 52.45, 24.06, 23.29, –0.36 ppm. HRMS-ESI (m/z) calcd for $[C_{25}H_{26}O_5SiNa]^+$ 457.1442 $[M + Na]^+$, found 457.1450. IR (thin film) 3466 (br), 2956, 2238, 2175, 1743 (s), 1436, 1292, 1251, 1213, 1070, 1039, 971, 846, 758, 690 cm^{-1}.

Preparation of tetrayne L5: To a solution of **S6** (1.35 g, 3.11 mmol) in anhydrous tetrahydrofuran (20 mL), a solution of (n-Bu)$_4$NF in tetrahydrofuran (1.0 M, 4.66 mL, 4.66 mmol) was added at –40 ℃. The mixture was stirred at the same temperature until the starting material disappeared (20 min). The mixture was then poured into a saturated aqueous solution of NH_4Cl (10 mL). The mixture was extracted with Et_2O (3 × 30 mL), and washed with saturated brine (2 × 20 mL). The organic layer was dried over $MgSO_4$ and concentrated to dryness. The residue was chromatographed with hexane/EtOAc (3:1) to afford **L5** as a light yellow solid. Yield: 1.00 g, 89%. ^{1}H NMR (400.1 MHz, $CDCl_3$): δ 7.28-7.48 (m, 5H), 5.09 (m, 1H), 3.80 (s, 6H), 3.17 (s, 2H), 3.07 (d, J = 1.84 Hz, 2H), 2.92 (br, 1H), 2.57 ppm (d, J = 2.32 Hz, 1H). ^{13}C NMR (100.6 MHz, $CDCl_3$): δ 168.89, 132.57, 129.20, 128.40, 121.47, 80.90, 80.88, 79.28, 77.71, 75.96, 73.75, 72.59, 68.35, 56.63, 53.41, 51.83, 24.06, 23.22 ppm. HRMS-ESI (m/z) calcd for $[C_{22}H_{18}O_5Na]^+$ 385.1046 $[M + Na]^+$, found 385.1049. IR (thin film) 3467 (br), 3292 (s), 2955, 2925, 2236, 2122, 1740 (s), 1490, 1436, 1333, 1293, 1214, 1144, 1070, 1053, 1025, 757, 690 cm^{-1}.

Synthesis pathway for L6.

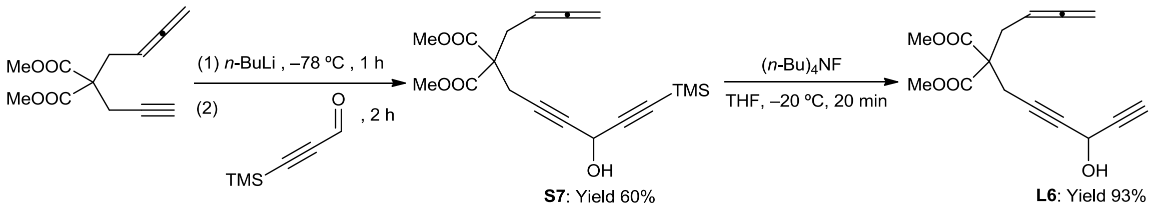

Preparation of compound S7: To an oven-dried flask containing dimethyl 2-(buta-2,3-dien-1-yl)-2-(prop-2-yn-1-yl)malonate (7.68 g, 34.6 mmol) and anhydrous tetrahydrofuran (400 mL), n-BuLi (14.4 mL, 2.4 M in anhydrous tetrahydrofuran, 34.6 mmol) was added dropwise over 45 min under N_2 at –78 ℃. After stirring the reaction mixture for an additional 15 min, (trimethylsilyl)propiolaldehyde (5.11 mL, 34.6 mmol) was added dropwise at the same temperature. After 2 h, the reaction was quenched with saturated aqueous NH_4Cl (150 mL), and warmed to room temperature. The resulting

mixture was extracted with Et_2O (3 × 150 mL), and the extract was washed with saturated brine (2 × 100 mL), dried with $MgSO_4$, and concentrated to dryness. The residue was chromatographed with hexane/EtOAc (6:1) to afford **S7** as a yellow oil. Yield: 7.23 g, 60%. ^{1}H NMR (500.2 MHz, $CDCl_3$): δ 5.03 (t, J = 1.85 Hz, 1H), 4.95 (tt, J = 8.00 Hz, J = 6.64 Hz, 1H), 4.68 (dt, J = 6.64 Hz, J = 2.41 Hz, 2H), 3.74 (s, 6H), 2.90 (d, J = 1.85 Hz, 1H), 2.76 (dt, J = 7.95 Hz, J = 2.41 Hz, 1H), 2.30 ppm (br, 1H), 0.18 (s, 9H). ^{13}C NMR (100.6 MHz, $CDCl_3$): δ 210.12, 170.07, 102.18, 88.79, 83.65, 80.85, 79.42, 74.82, 57.23, 52.82, 52.30, 31.80, 22.97, –0.39 ppm. HRMS-ESI (m/z) calcd for $[C_{18}H_{24}O_5SiNa]^+$ 371.1285 $[M + Na]^+$, found 371.1290. IR (thin film) 3466 (br), 2957, 2901, 2872, 2236, 2174, 1956, 1739 (s), 1438, 1289, 1251, 1211, 1145, 1078, 1039, 971, 845, 761 cm^{-1}.

Preparation of compound L6: To a solution of **S7** (7.23 g, 20.7 mmol) in anhydrous tetrahydrofuran (100 mL), a solution of (n-Bu)$_4$NF in tetrahydrofuran (1.0 M, 31.1 mL, 31.1 mmol) was added at –20 °C. The mixture was stirred at the same temperature until the starting material disappeared (20 min). The mixture was then poured into a saturated aqueous solutions of NH_4Cl (50 mL), extracted with Et_2O (3 × 50 mL), and washed with saturated brine (2 × 50 mL). The organic layer was dried over $MgSO_4$ and concentrated to dryness. The residue was chromatographed with hexane/EtOAc (3:1) to afford **L6** as a light yellow oil. Yield: 5.34 g, 93%. ^{1}H NMR (500.2 MHz, $CDCl_3$): δ 5.06 (dt, J = 2.30 Hz, J = 2.05 Hz, 1H), 4.97 (tt, J = 8.01 Hz, J = 6.66 Hz, 1H), 4.69 (dt, J = 6.66 Hz, J = 2.41 Hz, 2H), 3.75 (s, 6H), 2.91 (d, J = 2.05 Hz, 1H), 2.76 (dt, J = 8.01 Hz, J = 2.41 Hz, 1H), 2.55 (br, 1H), 2.54 ppm (d, J = 2.30 Hz, 1H). ^{13}C NMR (125.8 MHz, $CDCl_3$): δ 210.07, 170.11, 83.60, 81.11, 80.57, 79.49, 74.84, 72.28, 57.18, 52.86, 51.61, 31.80, 22.92 ppm. HRMS-ESI (m/z) calcd for $[C_{15}H_{16}O_5Na]^+$ 299.0890 $[M + Na]^+$, found 299.0894. IR (thin film) 3467 (br), 3289 (s), 2955, 2849, 2235, 2121, 1955, 1735, 1438, 1291, 1248, 1212, 1144, 1079, 1026, 850, 664 cm^{-1}.

Synthesis pathway for L7.

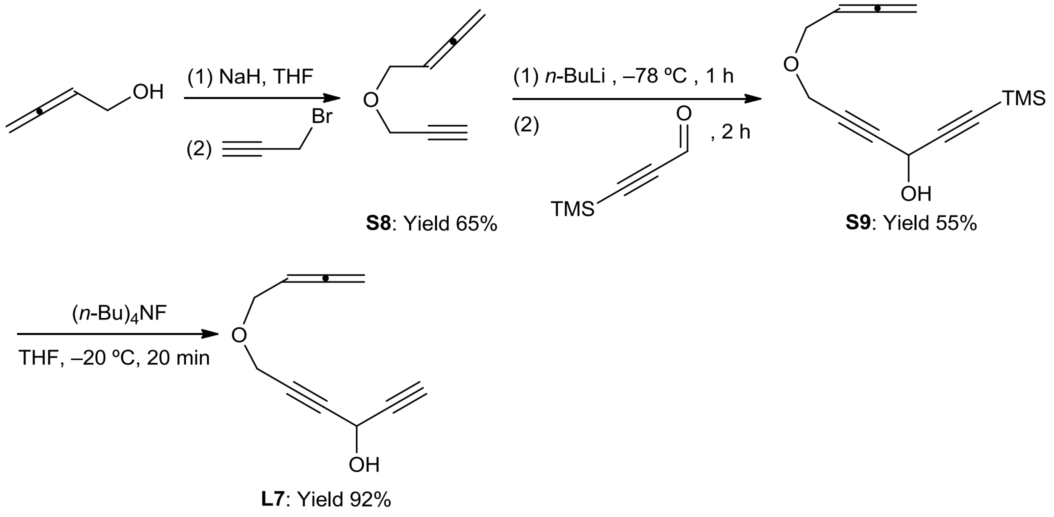

Preparation of compound S8: To an oven-dried three-necked flask, NaH (2.29g, 60% dispersion in mineral oil, 57.2 mmol) and anhydrous tetrahydrofuran (120 mL) was added sequentially under and N_2 atmosphere. After cooling to 0 °C, a solution of 2,3-butadien-1-ol (4.00 g, 57.1 mmol) in anhydrous tetrahydrofuran (30 mL) was added dropwise. The reaction mixture was then stirred at room temperature for 30 min, and 3-bromopropyne (4.92 ml, 57.1 mmol) was added dropwise at room temperature. After stirring for 24 h at room temperature, the reaction was quenched with a saturated aqueous solution of NH_4Cl (30 mL) at 0 °C, and then warmed to room temperature. The resulting mixture was extracted with Et_2O (3 × 100 mL). The extract was washed with saturated brine (2 × 100 mL), dried over $MgSO_4$, and concentrated to dryness. The compound was purified by distillation (88–92 °C at 75 torr) to afford **S8** as a colorless oil. Yield: 4.01 g, 65%. ^{1}H NMR (500.2 MHz, $CDCl_3$): δ 5.23 (tt, J = 7.01 Hz, J = 6.61 Hz, 1H), 4.82 (dt, J = 6.61 Hz, J = 2.10 Hz, 2H), 4.18 (d, J = 2.20 Hz, 2H), 4.11 (dt, J = 7.01 Hz, J = 2.10 Hz, 2H), 2.43 ppm (t, J = 2.20 Hz, 1H). ^{13}C NMR (125.8 MHz, $CDCl_3$): δ 209.65, 87.13, 78.62, 75.81, 74.50, 67.39, 56.81 ppm. HRMS-ESI (m/z) calcd for $[C_7H_8ONa]^+$ 131.0467 $[M + Na]^+$, found 131.0470. IR (thin film) 3287 (s), 2921, 2852, 2123, 1956, 1719, 1260, 1086, 1016, 853, 800, 662 cm^{-1}.

Preparation of compound S9: To an oven-dried flask containing **S8** (1.61 g, 14.9 mmol)

and anhydrous tetrahydrofuran (50 mL), *n*-BuLi (6.21 mL, 2.4 M in tetrahydrofuran, 14.9 mmol) was added dropwise over 45 min under N_2 at –78 °C. After the reaction mixture was stirred for an additional 15 min, (trimethylsilyl)propiolaldehyde (2.20 mL, 14.9 mmol) was added dropwise at the same temperature. After 2 h, the reaction was quenched with saturated aqueous NH_4Cl (50 mL), and warmed to room temperature. The resulting mixture was extracted with Et_2O (3 × 50 mL), and the extract was washed with saturated brine (2 × 50 mL), dried with $MgSO_4$, and concentrated to dryness. The residue was chromatographed with hexane/AcOEt (10:1) to afford **S9** as a light yellow oil. Yield: 1.92 g, 55%. ^{1}H NMR (500.2 MHz, $CDCl_3$): δ 5.24 (tt, apparent quit, *J* = 7.00 Hz, *J* = 6.60 Hz, 1H), 5.15 (dt, *J* = 7.42 Hz, *J* = 1.65 Hz, 1H), 4.82 (dt, *J* = 6.60 Hz, *J* = 2.40 Hz, 2H), 4.24 (d, *J* = 1.65 Hz, 2H), 4.10 (dt, *J* = 7.00 Hz, *J* = 2.40 Hz, 2H), 2.29 (d, *J* = 7.42 Hz, 1H), 0.19 (s, 9H). ^{13}C NMR (125.8 MHz, $CDCl_3$): δ 209.79, 101.80, 89.80, 87.17, 83.69, 80.56, 76.03, 67.72, 57.18, 52.68. HRMS-ESI (m/z) calcd for $[C_{13}H_{18}O_2SiNa]^+$ 257.0968 $[M + Na]^+$, found 257.0986. IR (thin film) 3396 (br), 2960, 2899, 2858, 2228, 2178, 1956, 1356, 1295, 1251, 1129, 1080, 1040, 966, 845, 761 cm^{-1}.

Preparation of Compound L7: To a solution of **S9** (1.92 g, 8.19 mmol) in anhydrous tetrahydrofuran (30 mL), a solution of $(n\text{-Bu})_4NF$ in tetrahydrofuran (1.0 M, 12.3 mL, 12.3 mmol) was added at –20 °C. The mixture was stirred at the same temperature until the starting material disappeared (20 min). The mixture was then poured into a saturated aqueous solution of NH_4Cl (50 mL), extracted with Et_2O (3 × 50 mL), and washed with saturated brine (2 × 50 mL). The organic layer was dried over $MgSO_4$ and concentrated to dryness. The residue was chromatographed with hexane/EtOAc (3:1) to afford **L7** as a light yellow oil. Yield: 1.22 g, 92%. ^{1}H NMR (400.1 MHz, $CDCl_3$): δ 5.22 (tt, apparent quit, *J* = 7.00 Hz, *J* = 6.59 Hz, 1H), 5.18 (dt, apparent q, *J* = 2.28 Hz, *J* = 1.68 Hz, 1H), 4.81 (dt, *J* = 6.59 Hz, *J* = 2.36 Hz, 2H), 4.23 (d, *J* = 1.68 Hz, 2H), 4.09 (dt, *J* = 7.00 Hz, *J* = 2.36 Hz, 2H), 2.71 (br, 1H), 2.57 (d, *J* = 2.28 Hz, 1H). ^{13}C NMR (100.6 MHz, $CDCl_3$): δ 209.57, 86.81, 83.45, 80.77, 80.13, 76.01, 72.81, 67.46, 56.81, 51.50. HRMS-ESI (m/z)

calcd for $[C_{10}H_{10}O_2Na]^+$ 185.0573 $[M + Na]^+$, found 185.0574. IR (thin film) 3383 (br), 3291 (s), 2919, 2854, 2121, 1955, 1356, 1299, 1263, 1128, 1077, 1027, 930, 853, 654 cm^{-1}.

Synthesis pathway for triyne L8.

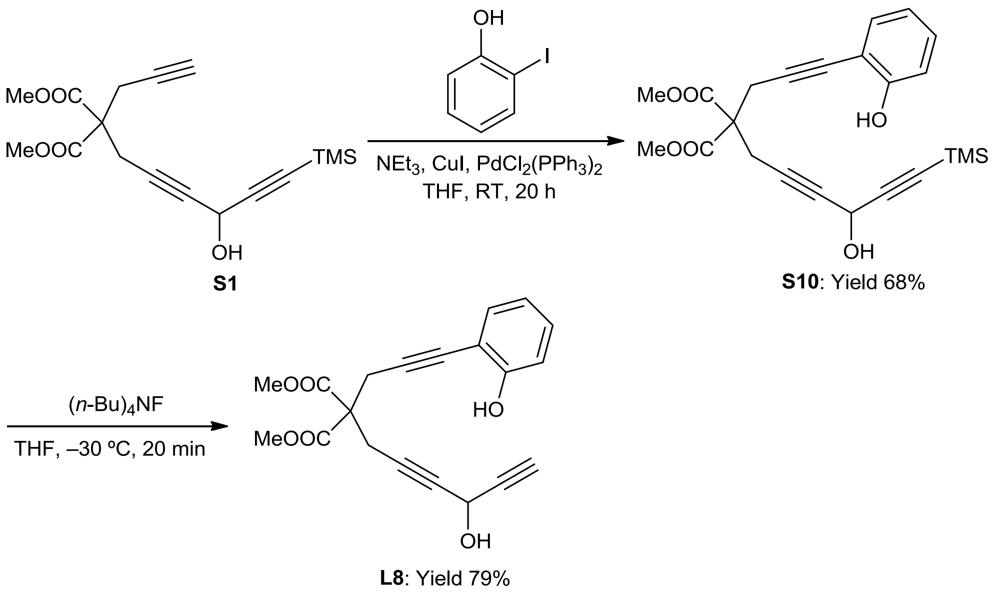

Preparation of compound S10: To a solution of 2-iodophenol (1.98 g, 9.00 mmol), $PdCl_2(PPh_3)_2$ (117 mg, 0.167 mmol) and CuI (63 mg, 0.331 mmol) in anhydrous tetrahydrofuran (200 mL) under a N_2 atmosphere, a anhydrous tetrahydrofuran solution (10 mL) of **S1** (2.01 g, 6.01 mmol) and dry triethylamine (12 mL) was added successively. The mixture was stirred at room temperature for 20 h. After total consumption of the aryl halide, the reaction mixture was filtered through celite. Celite was washed with dichloride methane (3 × 50 ml). The filtrate was washed with saturated solution of NH_4Cl (50 ml), saturated brine (3 × 50 mL) and dried over $MgSO_4$. Solvent was removed under vacuum. The residue was chromatographed with hexane/EtOAc (5:1) to afford **S10** as a yellow oil. Yield: 1.73 g, 68%. ^1H NMR: ^1H NMR (400.1 MHz, $CDCl_3$): δ 6.78-7.23 (Ph, 4H), 6.17 (s, 1H), 5.02 (dt, J = 7.24 Hz, J = 1.64 Hz, 1H), 3.77 (s, 6H), 3.20 (s, 2H), 3.05 (d, J = 1.64 Hz, 2H), 2.48 (d, J = 7.24 Hz, 1H), 0.15 ppm (s, 9H). ^{13}C NMR (100.6 MHz, $CDCl_3$): δ 169.99, 157.70, 131.71, 130.60, 120.42, 115.30, 109.44, 102.19, 91.15, 89.77,

81.70, 79.38, 79.06, 57.20, 53.75, 52.82, 24.73, 23.84, –0.03 ppm. HRMS-ESI (m/z) calcd for $[C_{23}H_{26}O_6SiNa]^+$ 449.1391 $[M + Na]^+$, found 449.1391. IR (thin film) 3477 (br), 3006, 2957, 2929, 2900, 2235, 2175, 1733, 1487, 1437, 1328, 1295, 1251, 1213, 1036, 847, 758 cm^{-1}.

Preparation of triyne L8: To a solution of **S10** (1.37 g, 3.21 mmol) in anhydrous tetrahydrofuran (100 mL), a solution of $(n\text{-}Bu)_4NF$ in tetrahydrofuran (1.0 M, 4.8 mL, 4.80 mmol) was added at –30 ℃. The mixture was stirred at the same temperature until the starting material disappeared (20 min). The mixture was then poured into a saturated aqueous solution of NH_4Cl (50 mL), extracted with Et_2O (3 × 50 mL), and washed with saturated brine (2 × 50 mL). The organic layer was dried over $MgSO_4$ and concentrated to dryness. The residue was chromatographed with hexane/EtOAc (3:1) to afford **L8** as brown oil. Yield: 0.90 g, 79%. ^1H NMR (400.1 MHz, $CDCl_3$): δ 6.80-7.26 (Ph, 4H), 6.22 (s, 1H), 5.07 (ddt, J = 7.51 Hz, J = 2.32 Hz, J = 2.04 Hz, 1H), 3.80 (s, 6H), 3.22 (s, 2H), 3.08 (d, J = 2.04 Hz, 2H), 2.91 (d, J = 7.51 Hz, 1H), 2.54 ppm (d, J = 2.32 Hz, 1H). ^{13}C NMR (100.6 MHz, $CDCl_3$): δ 169.99, 157.62, 131.74, 130.58, 120.41, 115.29, 109.41, 91.01, 81.34, 81.18, 79.54, 79.09, 72.88, 57.13, 53.77, 52.15, 24.72, 23.76 ppm. HRMS-ESI (m/z) calcd for $[C_{20}H_{18}O_6Na]^+$ 377.0996 $[M + Na]^+$, found 377.0994. IR (thin film) 3466 (br), 3288 (s), 3010, 2955, 2927, 2848, 2234, 2122, 1733 (s), 1487, 1437, 1330, 1296, 1214, 1152, 1074, 1057, 1024, 757 cm^{-1}.

Metal Complex Synthesis:

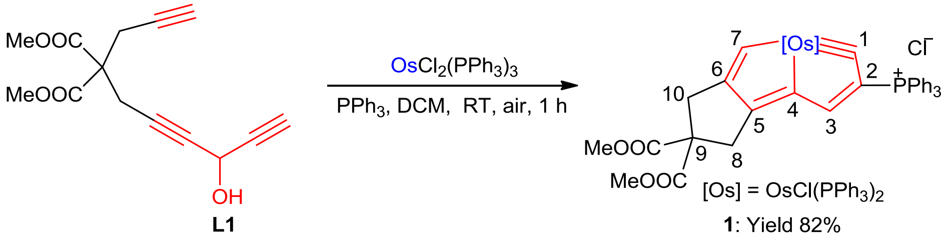

Preparation of complex 1: A dichloromethane solution (5 mL) of **L1** (1.20 g, 4.58 mmol) was added slowly to a green solution of $OsCl_2(PPh_3)_3$ (4.01 g, 3.83 mmol) and PPh_3 (5.02 g, 19.1 mmol; the excess PPh_3 may assist in increasing the stability of $OsCl_2(PPh_3)_3$) in dichloromethane (150 mL) under an air atmosphere. The reaction mixture was stirred at room temperature for 1 h to yield a brown solution. The solution was evaporated under vacuum to a volume of approximately 15 mL and then washed with Et_2O (3 × 200 mL) to afford a brown solid. The solid was purified by flash chromatography on silica gel (eluent: 20:1 dichloromethane/methanol) to yield complex **1** as a brown solid. Yield: 4.05 g, 82%. ^{1}H NMR (500.2 MHz, CD_2Cl_2): δ 13.14 (s, 1H, C^7H), 7.64 (s, 1H, C^3H), 7.00-7.77 (45H of Ph and 1H of C^3H mentioned above), 3.62 (s, 6H, COOCH_3), 3.09 (t, J(PH) = 3.62 Hz, 2H, $C^{10}H$), 2.41 ppm (s, 2H, C^8H). ^{31}P NMR (202.5 MHz, CD_2Cl_2): δ 5.77 (t, J(PP) = 4.84 Hz, CPPh$_3$), 3.32 ppm (d, J(PP) = 4.84 Hz, OsPPh$_3$). ^{13}C NMR (125.8 MHz, CD_2Cl_2, plus ^{13}C DEPT-135, ^{1}H-^{13}C HSQC and ^{1}H-^{13}C HMBC): δ 321.57 (dt, apparent q, J(PC) = 13.33 Hz, J(PC) = 13.33 Hz, C^1), 217.23 (dd, J(PC) = 19.55 Hz, J(PC) = 9.78 Hz, C^7), 173.07 (s, C^5), 172.16 (s, COOCH$_3$), 171.81 (d, J(PC) = 23.02 Hz, C^4), 170.12 (s, C^6), 149.22 (d, J(PC) = 16.31 Hz, C^3), 128.18-135.61 (Ph), 128.15 (dt, J(PC) = 81.30 Hz, J(PC) = 3.64 Hz, C^2), 120.30 (d, J(PC) = 91.07 Hz, Ph), 64.38 (s, C^9), 53.54 (s, COOCH$_3$), 39.07 (s, C^8), 37.62 ppm (s, C^{10}). Anal. Calcd (%) for $C_{68}H_{57}Cl_2O_4P_3Os$: C, 63.20; H, 4.45. Found: C, 63.25; H, 4.63. HRMS (ESI): *m/z* calcd for $[C_{68}H_{57}ClO_4P_3Os]^+$ 1257.2768 $[M]^+$, found 1257.2771.

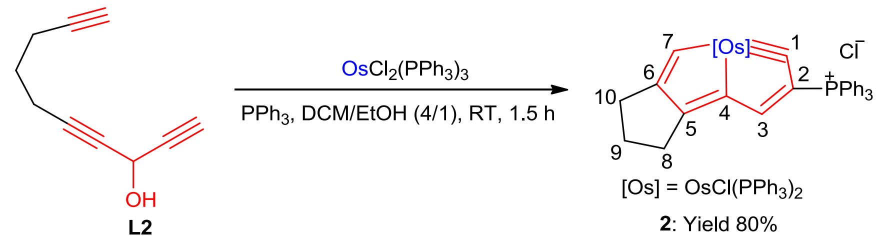

Preparation of complex 2: L2 (375 mg, 2.57 mmol) was added slowly to a green solution of $OsCl_2(PPh_3)_3$ (1.80 g, 1.72 mmol) and PPh_3 (1.80 g, 6.86 mmol) in a mixture of dichloromethane (40 mL) and ethanol (10 mL). The reaction mixture was stirred at room temperature for 1.5 h to yield a brown solution. The solvent completely removed under vacuum. The resulting residue was dissolved in dichloromethane and then washed with Et_2O (3 × 50 mL) to afford a brown solid. The solid was purified by flash chromatography (eluent: 20:1 dichloromethane/methanol) to yield complex **2** as a brown solid. Yield: 1.62 g, 80%. ^{1}H NMR (300.1 MHz, CD_2Cl_2): δ 13.34 (s, 1H, $C^{7}H$), 7.61 (s, 1H, $C^{3}H$), 7.02-7.80 (45H of Ph and 1H of $C^{3}H$ mentioned above), 2.44. (m, 2H, $C^{10}H$), 2.01 (tt, apparent quint, *J*(HH) = 7.27 Hz, *J*(HH) = 7.27 Hz, 2H, $C^{9}H$), 1.79 ppm (t, *J*(HH) = 7.27 Hz, 2H, $C^{8}H$). ^{31}P NMR (121.5 MHz, CD_2Cl_2): δ 5.76 (t, *J*(PP) = 5.72 Hz, C*P*Ph_3), 3.23 ppm (d, *J*(PP) = 5.72 Hz, Os*P*Ph_3). ^{13}C NMR (75.5 MHz, CD_2Cl_2, plus ^{13}C DEPT-135 , ^{1}H-^{13}C HSQC and ^{1}H-^{13}C HMBC): δ 321.56 (dt, apparent q, *J*(PC) = 13.33 Hz, *J*(PC) = 13.33 Hz, C^{1}), 216.59 (t, *J*(PC) = 10.59 Hz, C^{7}), 180.44 (s, C^{5}), 175.50 (t, *J*(PC) = 3.53 Hz, C^{6}), 172.04 (dt, *J*(PC) = 23.30 Hz, *J*(PC) = 3.21 Hz, C^{4}), 147.96 (dt, *J*(PC) = 15.36 Hz, *J*(PC) = 2.77 Hz, C^{3}), 128.00-135.48 (Ph), 125.89 (dt, *J*(PC) = 95.84 Hz, *J*(PC) = 3.61 Hz, C^{2}), 120.56 (d, *J*(PC) = 90.83 Hz , Ph), 31.32 (s, C^{8}), 29.58 (s, C^{10}), 29.29 ppm (s, C^{9}). Anal. Calcd (%) for $C_{64}H_{53}Cl_2P_3Os$: C, 65.36; H, 4.54. Found: C, 65.16; H, 4.86. HRMS (ESI): *m/z* calcd for $[C_{64}H_{53}ClP_3Os]^+$ 1141.2658 $[M]^+$, found 1141.2646.

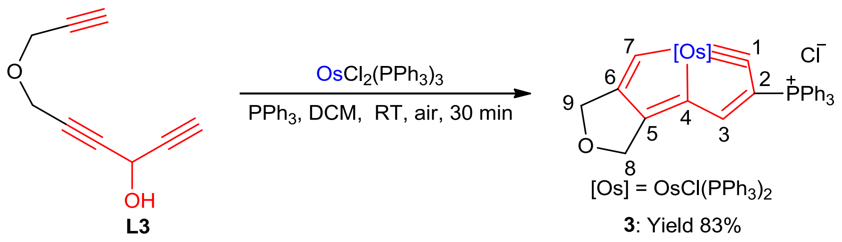

Preparation of complex 3: L3 (543 mg, 3.66 mmol) was added slowly to a green solution of $OsCl_2(PPh_3)_3$ (3.20 g, 3.05 mmol) and PPh_3 (4.00 g, 15.3 mmol) in dichloromethane (120 mL) under an air atmosphere. The reaction mixture was stirred at room temperature for 30 min to yield a brown solution. The solution was evaporated under vacuum to a volume of approximately 10 mL and washed with Et_2O (3 × 150 mL) to afford a brown solid. The solid was purified by flash chromatography on silica gel (eluent: 20:1 dichloromethane/methanol) to yield complex **3** as a brown solid. Yield: 2.98 g, 83%. ^{1}H NMR (600.1 MHz, CD_2Cl_2): δ 13.25 (s, 1H, C^7H), 7.66 (s, 1H, C^3H), 7.02-7.79 (45H of Ph and 1H of C^3H mentioned above), 4.53 (s, C^9H), 3.91 ppm (s, C^8H). ^{31}P NMR (242.9 MHz, CD_2Cl_2): δ 5.97 (t, J(PP) = 4.66 Hz, CPPh$_3$), 2.51 ppm (d, J(PP) = 4.66 Hz, OsPPh$_3$). ^{13}C NMR (150.9 MHz, CD_2Cl_2, plus ^{13}C DEPT-135, ^{1}H-^{13}C HSQC, and ^{1}H-^{13}C HMBC): δ 319.13 (dt, apparent q, J(PC) = 12.82 Hz, J(PC) = 12.82 Hz, C^1), 210.25 (br, C^7), 171.99 (s, C^5), 170.43 (s, C^6), 167.71 (d, J(PC) = 23.69 Hz, C^4), 148.70 (d, J(PC) = 14.86 Hz, C^3), 129.53-134.45 (Ph), 127.92 (dt, J(PC) = 95.56 Hz, J(PC) = 3.36 Hz, C^2), 126.99-127.05 (Ph), 118.85 (d, J(PC) = 91.08 Hz, Ph), 70.14 (s, C^8), 67.80 ppm (s, C^9). Anal. Calcd for $C_{63}H_{51}Cl_2OP_3Os$: C, 64.23; H, 4.36. Found: C, 64.43; H, 4.71. HRMS (ESI): m/z calcd for $[C_{63}H_{51}ClOP_3Os]^+$, 1143.2451 $[M]^+$; found, 1143.2435.

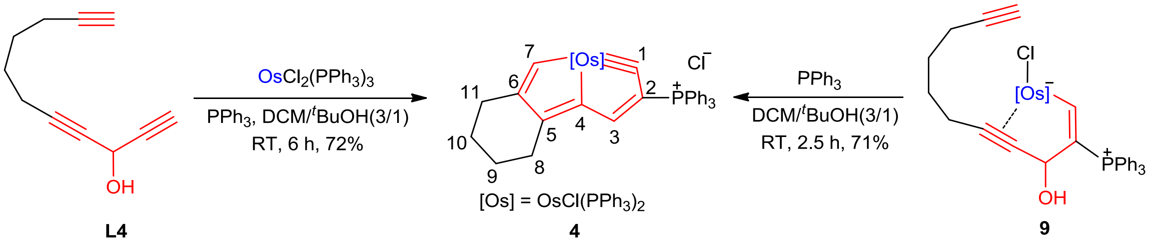

Preparation of complex 4: *Method A*: **L4** (1.10 g, 6.87 mmol) was added slowly to a green solution of $OsCl_2(PPh_3)_3$ (3.60 g, 3.44 mmol) and PPh_3 (3.60 g, 13.7 mmol) in a mixture of dichloromethane (75 mL) and tert-butanol (25 mL). The reaction mixture was stirred at room temperature for 6 h to yield a brown solution. The solvent was completely removed under vacuum. The resulting residue was dissolved in dichloromethane and then washed with Et_2O (3 × 150 mL) to afford a brown solid. The solid was purified by flash chromatography on silica gel (eluent: 20:1 dichloromethane/methanol) at 0 ℃ to yield complex **4** as a brown solid. Yield: 2.94 g, 72%. *Method B*: A light brown solution of **9** (350 mg, 0.290 mmol) and PPh_3 (300 mg, 1.14 mmol) in a mixture of dichloromethane (12 mL) and tert-butanol (4 mL) was stirred at room temperature for 2.5 h to yield a dark brown solution. The solvent was completely removed under vacuum. The resulting residue was dissolved in dichloromethane and then washed with Et_2O (3 × 20 mL) to afford a brown solid. The solid was purified by flash chromatography on silica gel (eluent: 20:1 dichloromethane/methanol) at 0 ℃ to yield complex **4** as a brown solid. Yield: 245 mg, 71%. ^{1}H NMR (600.1 MHz, CD_2Cl_2): δ 13.62 (s, 1H, C^7H), 7.69 (s, 1H, C^3H), 7.03-7.76 (46H, Ph and above-mentioned C^3H), 2.43 (s, 2H, $C^{11}H$), 2.01 (s, 2H, C^8H), 1.36 (s, 4H, C^9H and $C^{10}H$). ^{31}P NMR (242.9 MHz, CD_2Cl_2): δ 5.59 (t, *J*(PP) = 5.82 Hz, C*P*Ph$_3$), 3.82 ppm (d, *J*(PP) = 5.82 Hz, Os*P*Ph$_3$). ^{13}C NMR (150.9 MHz, CD_2Cl_2, plus ^{1}H-^{13}C HSQC and ^{1}H-^{13}C HMBC): δ 323.84 (dt, apparent q, *J*(PC) = 13.02 Hz, *J*(PC) = 13.02 Hz, C^1), 227.18 (br, C^7), 179.38 (d, *J*(PC) = 23.30 Hz, C^4), 167.82 (s, C^5), 164.45 (s, C^6), 143.14 (d, *J*(PC) = 16.09 Hz, C^3), 126.83-134.18 (Ph), 123.98 (dt, *J*(PC) = 96.16 Hz, *J*(PC) = 3.33 Hz, C^2), 119.48 (d, *J*(PC) = 90.86 Hz, Ph), 28.99 (s, C^{11}), 28.33 (s, C^8), 22.96 and 21.30 ppm (s, C^9 and C^{10}). Anal. Calcd (%) for $C_{65}H_{55}Cl_2P_3Os$: C, 65.59; H, 4.66. Found: C, 65.80; H, 4.98. HRMS (ESI): *m/z* calcd for $[C_{65}H_{55}ClP_3Os]^+$, 1155.2814 $[M]^+$; found, 1155.2823.

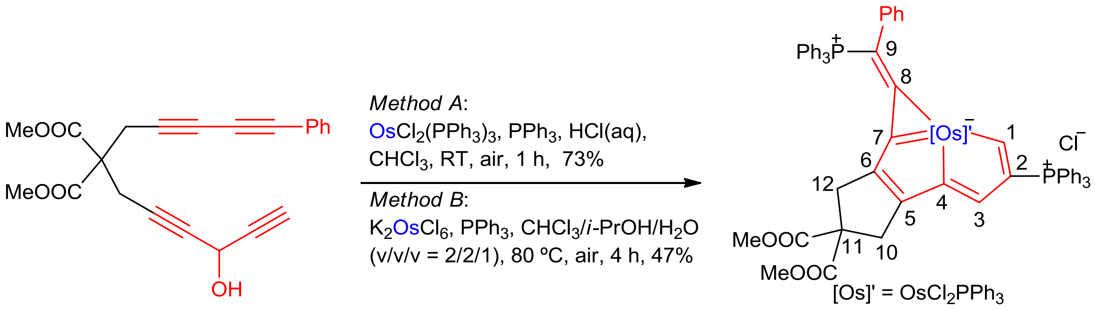

L5 **5**

Preparation of complex 5: *Method A*: HCl (1.30 M aqueous solution 146 μL, 0.190 mmol) and a chloroform solution (2 mL) of **L5** (70 mg, 0.193 mmol) were added to a green solution of $OsCl_2(PPh_3)_3$ (200 mg, 0.191 mmol) and PPh_3 (250 mg, 0.953 mmol) in chloroform (10 mL) under an air atmosphere. The reaction mixture was stirred at room temperature for 1 h to yield a red solution. The solvent was completely removed under vacuum. The resulting residue was dissolved in chloroform (2 mL) and washed with Et_2O (3 × 20 mL) to afford a red solid, and the solid was purified by column chromatography on silica gel (eluent: 20:1 dichloromethane/methanol) to yield complex **5** as a red solid. Yield: 199 mg, 73%. *Method B*: K_2OsCl_6 (203 mg, 0.422 mmol), PPh_3 (1.10 g, 4.19 mmol) and **L5** (153 mg, 0.422 mmol) in a mixture of chloroform (8 mL), isopropanol (8 mL) and water (4 mL) under an air atmosphere were stirred slowly at 80 ℃ for 4 h to yield a red solution. The solvent was completely removed under vacuum. The residue was dissolved in dichloromethane (5 mL) and washed with Et_2O (3 × 50 mL) to afford a red solid, and the solid was purified by column chromatography on silica gel (eluent: 20:1 dichloromethane/methanol) to afford complex **5** as a red solid. Yield: 283 mg, 47%. ^{1}H NMR (600.1 MHz, CD_2Cl_2): δ 13.95 (ddd, *J*(PH) = 17.06 Hz, *J*(PH) = 4.80 Hz, *J*(HH) = 2.72 Hz, 1H, C^1*H*), 8.19 (dd, apparent t, *J*(PH) = 2.72 Hz, *J*(HH) = 2.72 Hz, 1H, C^3*H*), 6.59-7.75 (50H, Ph), 3.63 (s, 3H, COOCH_3), 3.40 (s, 3H, COOCH_3), 2.41 (d, *J*(HH) = 19.45 Hz, 1H, C^{12}*H*), 1.89 (d, *J*(HH) = 18.11 Hz, 1H, C^{10}*H*), 1.71 (d, *J*(HH) = 18.11 Hz, 1H, C^{10}*H*), 1.29 ppm (d, *J*(HH) = 19.45 Hz, 1H, C^{12}*H*). ^{31}P NMR (242.9 MHz, CD_2Cl_2): δ 17.88 (s, C^9*P*Ph$_3$), 10.51 (s, C^2*P*Ph$_3$), –16.44 ppm (s, Os*P*Ph$_3$). ^{13}C NMR (150.9 MHz,

CD_2Cl_2, plus ^{13}C DEPT-135, ^{1}H-^{13}C HSQC and ^{1}H-^{13}C HMBC): δ 235.49 (br, C^1), 188.42 (d, J(PC) = 27.06 Hz, C^7), 182.66 (dd, apparent t, J(PC) = 4.41 Hz, J(PC) = 4.41 Hz, C^4), 178.47 (s, C^5), 170.49 (s, $COOCH_3$), 169.90 (s, $COOCH_3$), 164.09 (s, C^8), 163.30 (s, C^6), 142.06 (d, J(PC) = 22.61 Hz, C^3), 141.75 (d, J(PC) = 68.29 Hz, C^9), 126.88-134.13 (Ph), 119.15 (d, J(PC) = 87.95 Hz, Ph), 98.23 (d, J(PC) = 70.89 Hz, C^2), 64.11 (s, C^{11}), 52.57 (s, $COOCH_3$, determined by ^{1}H-^{13}C HSQC), 52.25 (s, $COOCH_3$), 36.26 (s, C^{12}), 35.78 ppm (s, C^{10}). Anal. Calcd (%) for $C_{76}H_{62}Cl_3O_4P_3Os$: C, 63.89; H, 4.37. Found: C, 64.00; H, 4.77. HRMS (ESI): m/z calcd for $[C_{76}H_{62}Cl_2O_4P_3Os]^+$ 1393.2847 $[M]^+$, found 1393.2847.

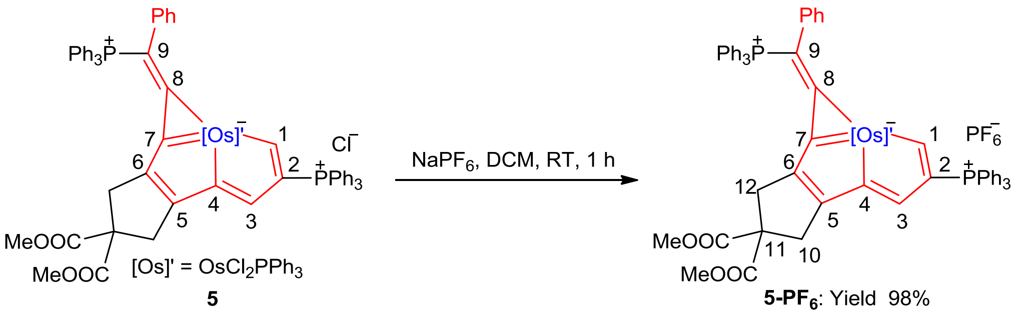

Preparation of complex 5-PF_6: $NaPF_6$ (84 mg, 0.500 mmol) was added to a red solution of **5** (150 mg, 0.105 mmol) in dichloromethane (10 mL). The reaction mixture was stirred at room temperature for 1 h to yield a red solution. The solid was removed by filtration, and the filtrate was evaporated under vacuum to afford a red solid. Yield: 158 mg, 98%. ^{1}H NMR (600.1 MHz, CD_2Cl_2): δ 13.95 (ddd, J(PH) = 16.99 Hz, J(PH) = 4.53 Hz, J(HH) = 2.75 Hz, 1H, C^1H), 8.19 (dd, apparent t, J(PH) = 2.75 Hz, J(HH) = 2.75 Hz, 1H, C^3H), 6.89-7.75 (50H, Ph), 3.63 (s, 3H, $COOCH_3$), 3.40 (s, 3H, $COOCH_3$), 2.41 (d, J(HH) = 19.53 Hz, 1H, $C^{12}H$), 1.89 (d, J(HH) = 18.16 Hz, 1H, $C^{10}H$), 1.71 (d, J(HH) = 18.16 Hz, 1H, $C^{10}H$), 1.29 ppm (d, J(HH) = 19.53 Hz, 1H, $C^{12}H$). ^{31}P NMR (242.9 MHz, CD_2Cl_2): δ 17.88 (s, C^9PPh_3), 10.51 (s, C^2PPh_3), –16.42 (s, $OsPPh_3$), –144.50 ppm (septet, J(FP) = 710.41 Hz, PF_6). ^{13}C NMR (150.9 MHz, CD_2Cl_2, plus ^{13}C DEPT-135, ^{1}H-^{13}C HSQC and ^{1}H-^{13}C HMBC): δ 235.52 (br, C^1), 188.43 (d, J(PC) = 25.46 Hz, C^7), 182.67 (dd, apparent

t, J(PC) = 4.30 Hz, J(PC) = 4.30 Hz, C^4), 178.49 (s, C^5), 170.52 (s, *C*OOCH$_3$), 169.92 (s, *C*OOCH$_3$), 164.10 (s, C^8), 163.32 (s, C^6), 142.08 (d, J(PC) = 22.59 Hz, C^3), 141.77 (d, J(PC) = 70.78 Hz, C^9), 126.70-134.13 (Ph), 119.18 (d, J(PC) = 88.28 Hz, Ph), 98.25 (d, J(PC) = 70.84 Hz, C^2), 64.13 (s, C^{11}), 52.56 (s, COO*C*H$_3$, determined by ^{1}H-^{13}C HSQC), 52.24 (s, COO*C*H$_3$), 36.27 ppm (s, C^{12}), 35.79 (s, C^{10}). Anal. Calcd (%) for $C_{76}H_{62}Cl_2F_6O_4P_4Os$: C, 59.34; H, 4.06. Found: C, 59.11; H, 4.42. HRMS (ESI): *m/z* calcd for $[C_{76}H_{62}Cl_2O_4P_3Os]^+$ 1393.2847 $[M]^+$, found 1393.2846.

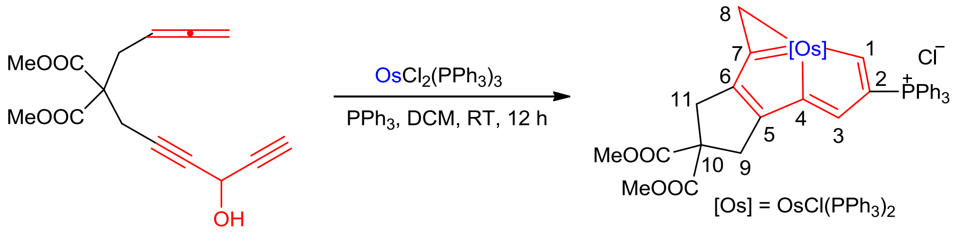

Preparation of complex 6: A dichloromethane solution (2 mL) of **L6** (135 mg, 0.489 mmol) was added slowly to a green solution of $OsCl_2(PPh_3)_3$ (425 mg, 0.406 mmol) and PPh_3 (538 mg, 2.05 mmol) in dichloromethane (15 mL). The reaction mixture was stirred at room temperature for 12 h to yield a brown solution. The solution was evaporated under vacuum to a volume of approximately 2 mL and washed with Et_2O (3 × 25 mL) to afford a yellow solid. The solid was purified by flash chromatography on silica gel (eluent: 20:1 dichloromethane/methanol) to give complex **6** as a brown solid. Yield: 273 mg, 51%. ^{1}H NMR (600.1 MHz, CD_2Cl_2): δ 13.84 (d, J(PH) = 17.52, 1H, C^1*H*), 8.09 (s, 1H, C^3*H*), 6.91–7.80 (45H, Ph), 3.71 (s, 6H, COOC*H*$_3$), 3.14 (s, 2H, C^{11}*H*) , 2.56 (s, 2H, C^8*H*), 1.90 ppm (s, 2H, C^9*H*). ^{31}P NMR (242.9 MHz, CD_2Cl_2): δ 11.74 (s, C*P*Ph$_3$), –12.53 ppm (s, Os*P*Ph$_3$). ^{13}C NMR (150.9 MHz, CD_2Cl_2, plus ^{13}C–dept 135, ^{1}H–^{13}C HSQC and ^{1}H–^{13}C HMBC): δ 235.97 (br, C^1), 228.94 (t, J(PC) = 4.41 Hz, C^7), 182.50 (s, C^5), 180.42 (d, J(PC) = 22.32 Hz, C^4), 170.33 (s, *C*OOCH$_3$), 161.76 (s, C^6), 144.00 (d, J(PC) = 22.01 Hz, C^3), 134.36 (dt, J(PC) = 64.20 Hz, J(PC) = 4.45 Hz, C^2), 126.93–134.15 (Ph), 119.03 (d, J(P,C) = 88.08 Hz, Ph), 64.96 (s, C^{10}), 52.58 (s, COO*C*H$_3$), 38.01 (s, C^9), 33.26 (s,

C^{11}), 27.44 ppm (s, C^8). Anal. Calcd for $C_{69}H_{59}Cl_2O_4P_3Os$: C, 63.44; H, 4.55. Found: C, 63.54; H, 4.89. HRMS (ESI): *m/z* calcd for $[C_{69}H_{59}ClO_4P_3Os]^+$ 1271.2924 $[M]^+$, found 1271.2932.

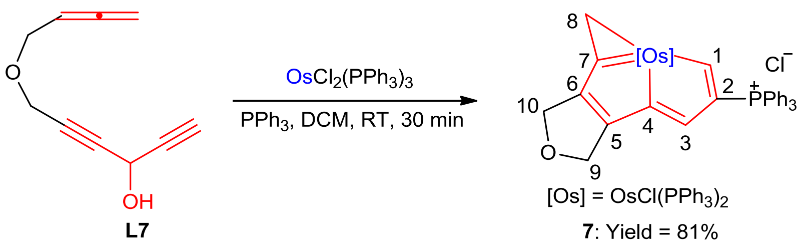

Preparation of complex 7: A dichloromethane solution (2 mL) of **L7** (130 mg, 0.802 mmol) was added slowly to a green solution of $OsCl_2(PPh_3)_3$ (550 mg, 0.525 mmol) and PPh_3 (692 mg, 2.64 mmol) in dichloromethane (20 mL). The reaction mixture was stirred at room temperature for 30 min to yield a brown solution. The solution was evaporated under vacuum to a volume of approximately 2 mL and washed with Et_2O (3 × 30 mL) to afford a brown solid. The solid was purified by flash chromatography on silica gel (eluent: 20:1 dichloromethane/methanol) to yield complex **7** as a yellow solid. Yield: 506 mg, 81%. ^{1}H NMR (500.2 MHz, CD_2Cl_2): δ 14.01 (d, *J*(PH) = 17.17 Hz, 1H, C^1H), 8.21 (s, 1H, C^3H), 6.90-7.81 (45H, Ph), 4.12 (s, 2H, C^9H), 3.21 (s, 2H, $C^{10}H$), 3.03 (s, 2H, C^8H). ^{31}P NMR (202.5 MHz, CD_2Cl_2): δ 11.81 (t, *J*(PP) = 4.95 Hz, C*P*Ph$_3$), –13.19 (d, *J*(PP) = 4.95 Hz, Os*P*Ph$_3$). ^{13}C NMR (125.8 MHz, CD_2Cl_2, plus ^{13}C DEPT-135, ^{1}H-^{13}C HSQC and ^{1}H-^{13}C HMBC): δ 239.19 (br, C^1), 226.16 (t, *J*(PC) = 4.37 Hz, C^7), 183.46 (s, C^5), 176.00 (d, *J*(PC) = 25.20 Hz, C^4), 163.13 (s, C^6), 145.12 (d, *J*(PC) = 21.87 Hz, C^3), 134.66 (dt, *J*(PC) = 70.73 Hz, *J*(PC) = 3.92 Hz, C^2), 118.46-134.17 (Ph), 69.92 (s, C^{10}), 65.41 (s, C^9), 23.08 (s, C^8). Anal. Calcd calcd (%) for $C_{64}H_{53}Cl_2OP_3Os$: C, 64.48; H, 4.48. Found: C, 64.32; H, 4.77. HRMS (ESI): *m/z* calcd for $[C_{64}H_{53}ClOP_3Os]^+$, 1157.2607 $[M]^+$; found, 1157.2594.

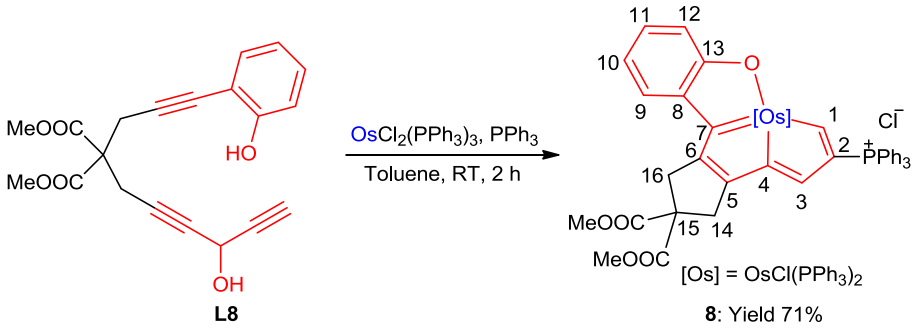

Preparation of complex 8: A toluene solution (5 mL) of **L8** (570 mg, 1.61 mmol) was added in one portion to a green solution of $OsCl_2(PPh_3)_3$ (1.36 g, 1.30 mmol) and PPh_3 (1.70 g, 6.48 mmol) in toluene (35 mL). The reaction mixture was stirred slowly at room temperature for 2 h to yield a green precipitate. Complex **8** was obtained as a green solid by filtration and washing with Et_2O (3 × 40 mL). Yield: 1.28 g, 71%. ^{1}H NMR (600.1 MHz, CD_2Cl_2): δ 14.09 (ddd, J(PH) = 14.93 Hz, J(PH) = 5.52 Hz, J(PH) = 2.82 Hz, 1H, C^1H), 7.81 (C^3H, determined by HSQC), 7.11 (C^9H, determined by HSQC), 7.08 ($C^{10}H$, determined by HSQC), 6.90-7.82 (47H, Ph and C^9H, $C^{10}H$ mentioned-above), 6.68 (d, J(PH) = 8.58 Hz, 1H, $C^{12}H$), 6.56 (dd, apparent t, J(PH) = 8.58 Hz, J(PH) = 7.45 Hz, 1H, $C^{11}H$), 3.75 (s, 6H, $COOCH_3$), 3.20 ppm (s, 4H, $C^{14}H$ and $C^{16}H$). ^{31}P NMR (242.9 MHz, CD_2Cl_2): δ 11.98 (s, $CPPh_3$), –17.05 ppm (s, $OsPPh_3$). ^{13}C NMR (150.9 MHz, CD_2Cl_2, plus ^{13}C DEPT-135, ^{1}H-^{13}C HSQC and ^{1}H-^{13}C HMBC): δ 227.35 (t, J(PC) = 6.11 Hz, C^7), 209.86 (br, C^1), 184.71 (s, C^5), 181.12 (s, C^6), 175.94 (dt, J(PC) = 23.71 Hz, J(PC) = 3.92 Hz, C^4), 170.02 (s, $COOCH_3$), 160.25 (s, C^8), 138.49 (d, J(PC) = 19.38 Hz, C^3), 137.60 (s, C^{13}), 135.16 (s, C^{10}), 128.98-134.42 (Ph), 126.88 (d, J(PC) = 79.82 Hz, C^2), 126.68-126.74 (Ph), 125.25 (s, C^9), 120.45 (s, C^{12}), 118.71 (d, J(PC) = 89.14 Hz, Ph), 116.92 (s, C^{11}), 63.76 (s, C^{15}), 52.82 (s, $COOCH_3$), 37.35 and 35.83 (s, C^{14} and C^{16}). Anal. Calcd calcd (%) for $C_{74}H_{61}Cl_2O_5P_3Os$: C, 64.20; H, 4.44. Found: C, 63.87; H, 4.81. HRMS (ESI): m/z calcd for $[C_{74}H_{61}ClO_5P_3Os]^+$, 1349.3030 $[M]^+$; found, 1349.3033.

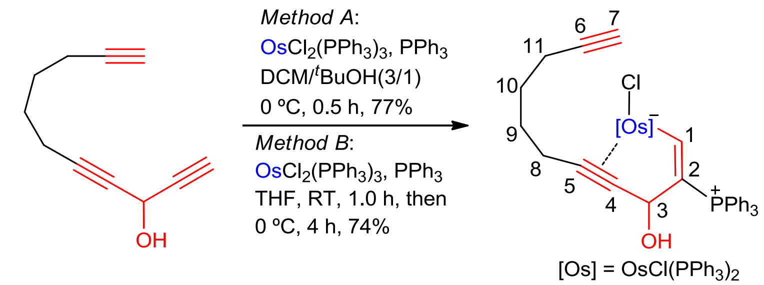

L4 **9**

Preparation of complex 9: *Method A*: **L4** (140 mg, 0.874 mmol) was added to a green solution of $OsCl_2(PPh_3)_3$ (450 mg, 0.429 mmol) and PPh_3 (500 mg, 1.91 mmol) in a mixture of dichloromethane (9 mL) and tert-butanol (3 mL). The reaction mixture was stirred at 0 ℃ for 0.5 h to yield a brown solution. The solution was evaporated under vacuum to a volume of approximately 6 mL and washed with *n*-hexane (3 × 20 mL) to afford a brown solid. Yield: 398 mg, 77%. *Method B*: To a green solution of $OsCl_2(PPh_3)_3$ (900 mg, 0.859 mmol) and PPh_3 (1.13 g, 4.31 mmol) in anhydrous tetrahydrofuran (10 mL), **L4** (153 mg, 0.955 mmol) was added. The reaction mixture was stirred at room temperature for 1.0 h and then cooled to 0 ℃ for 4 h without stirring to yield a brown suspension. The yellow solid was collected by filtration, washed with THF (2 × 5 mL) and then dried under vacuum. Yield: 768 mg, 74%. ^{1}H NMR (600.1 MHz, Tetrahydrofuran-d8): δ 12.21 (d, *J*(PH) = 18.30 Hz, 1H, C^{1}*H*), 6.95-7.99 (45H, Ph), 5.93 (br, 1H, C^{3}*H*), 4.47 (d, *J*(HH) = 4.50 Hz, 1H, O*H*), 2.16 (t, *J*(HH) = 2.58 Hz, 1H, C^{7}*H*), 1.75 (td, *J*(HH) = 7.08 Hz, *J*(HH) = 2.58 Hz, 2H, C^{11}*H*), 0.81 (tt, apparent quint, *J*(HH) = 7.08 Hz, *J*(HH) = 7.08 Hz, 2H, C^{10}*H*), 0.80 (m, 1H, C^{8}*H*), 0.73 (m, 1H, C^{9}*H*), 0.55 (m, 1H, C^{9}*H*), 0.18 ppm (m, 1H, C^{8}*H*). ^{31}P NMR (242.9 MHz, Tetrahydrofuran-d8): δ 7.76 (s, C*P*Ph_3), –7.70 ppm (d, *J*(PP) = 411.17 Hz, Os*P*Ph_3), –16.02 ppm (d, *J*(PP) = 411.17 Hz, Os*P*Ph_3). ^{13}C NMR (150.9 MHz, Tetrahydrofuran-d8, plus ^{13}C DEPT-135 , ^{1}H-^{13}C HSQC and ^{1}H-^{13}C HMBC): δ 214.16 (m, C^{1}), 127.32-138.61 (Ph), 123.80 (d, *J*(PC) = 86.70 Hz, Ph), 109.52 (d, *J*(PC) = 74.70 Hz, C^{2}), 86.03 (d, *J*(PC) = 3.60 Hz, C^{5}), 85.38 (s, C^{6}), 80.53 (d, *J*(PC) = 24.72 Hz, C^{3}), 78.68 (d, *J*(PC) = 21.78 Hz, C^{4}), 69.02 (s, C^{7}), 30.76 (s, C^{10}), 30.73 (s, C^{9}), 24.15 (s, C^{8}), 18.96 (s, C^{11}). Anal. Calcd (%) for $C_{65}H_{57}Cl_2OP_3Os$: C,

64.62; H, 4.76. Found: C, 64.88; H, 4.65. HRMS (ESI): *m/z* calcd for $[C_{65}H_{57}ClOP_3Os]^+$ 1173.2920 $[M - Cl]^+$, found 1173.2941.

Supplementary References

1. Richard, V. *et al.* Iron(II)-catalysed [2+2+2] cycloaddition for pyridine ring construction. *Chem. Commun.* **50,** 593–595 (2014).

2. Journet, M., Cai, D., DiMichele, L. M. & Larsen, R. D. Highly efficient synthesis of α,β-acetylenic aldehydes from terminal alkynes using DMF as the formylating reagent. *Tetrahedron Lett.* **39,** 6427–6428 (1998).

3. Iafe, R. G. *et al.* Increasing the efficiency of the transannular Diels–Alder strategy via palladium(II)-catalyzed macrocyclizations. *Org. Lett.* **15,** 582–585 (2013).

4. Takahashi, Y. *et al.* Mono- and dipalladium movement on the π-conjugated five-carbon chain. *Organometallics* **27,** 276–280 (2008).

5. Kumareswaran, R., Shin, S., Gallou, I. & RajanBabu, T. V. Silylstannylation of allenes and silylstannylation–cyclization of allenynes. Synthesis of highly functionalized allylstannanes and carbocyclic and heterocyclic compounds. *J. Org. Chem.* **69,** 7157–7170 (**2004**).

6. Luo, H. & Ma, S. CuI-catalyzed synthesis of functionalized terminal allenes from 1-alkynes. *Eur. J. Org. Chem.* 3041–3048 (2013).

7. Rigaku Oxford Diffraction, CrysAlisPro, Version 1.171.38.43; Rigaku Corporation, Oxford, UK, 2015.

8. Rigaku Oxford Diffraction, CrysAlisPro, Version 1.171.39.30t; Rigaku Corporation, Oxford, UK, 2017.

9. Dolomanov, O. V.; Bourhis, L. J.; Gildea, R. J; Howard, J. A. K. & Puschmann, H. OLEX2: a complete structure solution, refinement and analysis program. *J. Appl. Cryst*. **42,** 339–341 (2009).

10. Sheldrick, G. M. *Acta Cryst*. A short history of SHELX. **A64,** 112–122 (2008).

11. Sheldrick, G. M. *Acta Cryst*. SHELXT-Integrated space-group and crystal-structure determination. **A71,** 3–8 (2015).

12. Sheldrick, G. M. *Acta Cryst*. Crystal structure refinement with SHELXL. **C71,** 3–8 (2015).
