# Supplementary material for: Multiyne chains chelating osmium via three metal-carbon σ bonds
Source: Nat Commun. 2017 Dec 4;8:1912. doi: 10.1038/s41467-017-02120-z (PMC5714968; doi:10.1038/s41467-017-02120-z)
Supplement: Supplementary file 3 — Description of Additional Supplementary Files [file 41467_2017_2120_MOESM3_ESM.pdf]

Description of Additional Supplementary Files

File Name: Supplementary Data 1
Description: Cartesian coordinates-optimized structures for ASE, NICS and AICD calculations.

File Name: Supplementary Data 2
Description: Cartesian coordinates-optimized structures for mechanism studies.
